# Anticancer Ruthenium Complexes with HDAC Isoform Selectivity

**DOI:** 10.3390/molecules25102383

**Published:** 2020-05-21

**Authors:** Jasmine M. Cross, Tim R. Blower, Alexander D. H. Kingdon, Robert Pal, David M. Picton, James W. Walton

**Affiliations:** 1 Department of Chemistry, Durham University, Lower Mountjoy, South Road, Durham DH1 3LE, UK; jmcross92@gmail.com (J.M.C.); aleckingdon@gmail.com (A.D.H.K.); robert.pal@durham.ac.uk (R.P.); 2 Department of Biosciences, Durham University, Stockton Road, Durham DH1 3LE, UK; timothy.blower@durham.ac.uk (T.R.B.); david.m.picton@durham.ac.uk (D.M.P.)

**Keywords:** histone deacetylase inhibitors, ruthenium in medicine, selective enzyme inhibition

## Abstract

The histone deacetylase (HDAC) enzymes have emerged as an important class of molecular targets in cancer therapy, with five inhibitors in clinical use. Recently, it has been shown that a lack of selectivity between the 11 Zn-dependent HDAC isoforms may lead to unwanted side-effects. In this paper, we show that piano stool Ru complexes can act as HDAC inhibitors, and variation in the capping arene leads to differences in HDAC isoform selectivity.

## 1. Introduction

**Figure 1:**
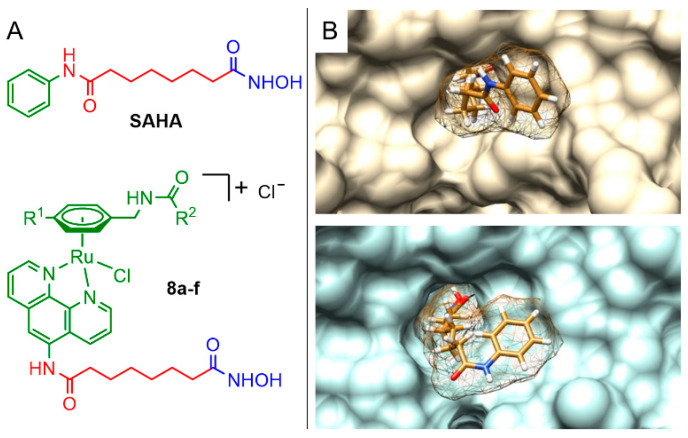
(**A**) HDAC pan inhibitor SAHA and Ru complexes **8a**–**f**, described in this work. (**B**) Docking models of SAHA with isoforms HDAC1 (upper) and HDAC6 (lower), showing the differences in active site cavity entrances.

The histone deacetylase (HDAC) enzymes control the extent of acetylation of ε-lysine residues within the histone core. Hypoacetylation leads to a charged histone core and a condensed chromatin structure, resulting in the suppression of gene expression [1,2,3]. HDACs are involved in the deacetylation of p53, a transcription factor involved in tumour suppression, which leads to its degradation and hence allows cancer cell progression [4]. HDACs are also associated with other functions, including angiogenesis, DNA damage repair and cell cycle control [5]. Five HDAC inhibitors have been approved for clinical use [6]. The archetypal inhibitor is suberanilohydroxamic acid (SAHA, Figure 1A) [7,8]. It incorporates a hydrophobic chain (red) that terminates with a hydroxamic acid (blue), which binds to a Zn(II) ion located at the bottom of a hydrophobic channel in the enzyme active site. A phenyl head group (green) sits in the cavity entrance of the active site (Figure 1B) [9]. SAHA, along with each of the clinically approved drugs, is a pan-inhibitor, acting on all 11 known Zn-dependent HDAC isoforms. However, it has recently been shown that pan-inhibition may lead to genotoxicity [10,11], and that targeting specific HDAC isoforms could be a better approach to target cancer progression [12,13,14]. Hence, the ability to inhibit isoforms selectively is at the forefront of research in this area, with several isoform-selective HDAC inhibitors in clinical trials [5]. As shown in Figure 1B, key differences in HDAC isoforms are present in the cavity entrance region, where the capping phenyl group of SAHA binds. As such, variation in the inhibitor head group has the potential to lead to isoform-selective inhibitors.

Transition metal complexes have emerged as promising candidates for selective enzyme inhibition [15,16]; they have more complex structural geometry than simple sp^2^/sp^3^-centred organic molecules, and their coordinated ligands can exchange with biological targets. Meggers has led the way in this field, with a series of PIM-1 kinase inhibitors, in which an organic heterocycle in staurosporine is replaced by a Ru complex, leading to an increase in selectivity towards PIM-1, a proto-oncogene that is implicated in multiple human cancers [17]. Other examples of metal-based inhibitors of carbonic anhydrase [18,19] and glutathione-S-transferase [20] have also been demonstrated.

Metal-based HDAC inhibitors have been reported, in which either the hydroxamic acid group acts as a ligand to the metal [21] or the phenyl capping group of SAHA is replaced by a metal complex (e.g., ferrocene [22,23,24], square planar Pt(II) [25,26], octahedral Ru(II) [27], Re(I) [28] and Ir(III) [29]). Examples of isoform-selectivity for metal-based HDAC inhibitors have appeared for ferrocene [22,24,30] and Ir complexes [31].

We recently showed that replacing the phenyl head group in SAHA for Ru piano stool complexes gives viable HDAC inhibitors [32]. We hypothesised that increasing the size of the capping arene group of the Ru complex could lead to improved selectivity towards HDAC6, which is seen to have a wider active site cavity entrance. In the work herein, we show that variation in the η^6^-coordinated arene of Ru piano stool complexes (Figure 1A) leads to modulation of isoform selectivity between HDAC1 and HDAC6, and we use computational docking experiments to rationalise these differences. We also show that this family of Ru complexes have potential as anticancer agents in vitro.

## 2. Results and Discussion

### 2.1. Synthesis and Characterisation

**Scheme 1:**
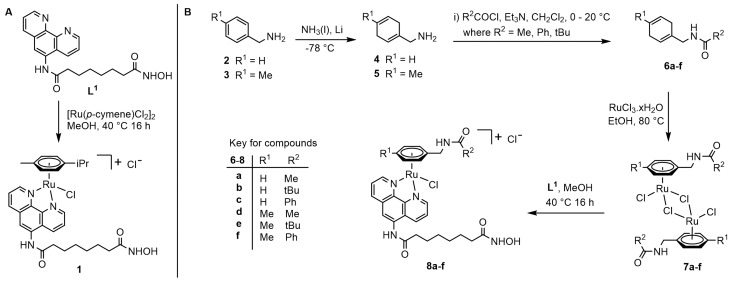
(**A**) Synthesis of complex 1 and (**B**) synthesis of complexes **8a**–**f**.

As previously described, complex **1** was synthesised through the reaction of ligand **L^1^** with the dimer [(*p*-cymene)RuCl_2_]_2_ (Scheme 1A) [32]. To introduce structural variation in the capping η^6^-coordinated arene, aryl precursors benzylamine and 4-methylbenzylamine were reacted via Birch reduction to give 1,4-cyclohexadienes **4** and **5**, respectively (Scheme 1B). Compounds **4** and **5** were coupled with acyl chlorides to give amides **6a**–**f**, which were reacted with RuCl_3_·xH_2_O to give the corresponding Ru metal dimers **7a**–**f**. Complexation with **L^1^** afforded complexes **8a**–**f**, which were purified by preparative reverse phase high performance liquid chromatography (HPLC). Formation and purity of the complexes were confirmed using ^1^H NMR spectroscopy, mass spectrometry, analytical HPLC and elemental analysis. The resulting complexes fall into two sets: **8a**–**c**, with a η^6^-phenyl capping group, and **8d**–**f**, with a η^6^-tolyl capping group. Within each set, the amide group in the capping ligand incorporates a methyl, *t*-butyl or phenyl group at position R^2^. The aqueous stability of complex **1** was monitored by ^1^H-NMR. After 1 h in D_2_O, the Ru-Cl bond remained fully intact, and after 96 h only around 10% hydrolysis was observed (Figure S2).

### 2.2. Enzyme Inhibition Assays

**Figure 2:**
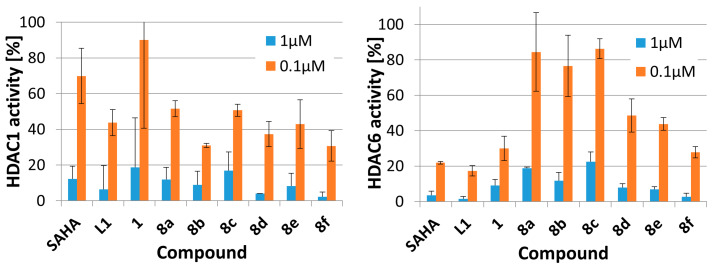
HDAC1 and HDAC6 inhibition assays of Ru complexes **1** and **8a**–**f**, and control compounds, measured using commercially available assay kits. All assays carried out in triplicate. See Supplementary Materials for details.

To begin to understand whether variation in the Ru capping ligand gives rise to HDAC isoform selectivity, we carried out fluorimetric assays of complexes **8a**–**f**, SAHA and **L^1^**, against HDAC1 (Class I) and HDAC6 (Class IIb) isoforms at two inhibitor concentrations. These isoforms were chosen as they provide contrasting binding topographies, and HDAC6 in particular is closely linked with cancer metastasis (Figure 2).

All new complexes **8a**–**f** demonstrated HDAC-inhibitory activity, reducing both HDAC1 and HDAC6 activity to less than 25% at 1 µM inhibitor. Against HDAC1, SAHA, **L^1^** and all complexes showed very similar activity at a given concentration. Against HDAC6, SAHA and **L^1^** showed high inhibitory potency. Within the series of Ru complexes, those with the η^6^-tolyl capping group (**8d**–**f**) show improved inhibitory activity compared to complexes with the η^6^-phenyl capping group (**8a**–**c**). The more bulky and more hydrophobic tolyl group likely provides more favourable interaction with the catalytic channel rim of HDAC6, which is known to be wider and shallower than other HDAC isoforms [33]. In a similar way, the selectivity of the known HDAC6-selective inhibitor tubastatin A originates from its ability to interact with the wide HDAC6 opening [34]. Of the new Ru complexes, **8f**, which incorporates a phenyl group at the R^2^ amide position, shows the highest activity against HDAC6, potentially due to aromatic π-stacking interactions between the R^2^ phenyl group and the aromatic amino acids found at the cavity entrance of HDAC6 (e.g., Phe199, Phe200, Figure S21).

For the candidates with the leading HDAC-inhibitory profiles, complexes **1** and **8f**, inhibitory concentration (IC_50_) values were measured against HDAC1 and HDAC6 (Table 1). Selectivity factors were calculated as the ratio of the IC_50_ values for HDAC1 and HDAC6. Data for **L^1^** and SAHA were also collected for comparison. Under the assay conditions, SAHA was more active against HDAC6, compared to HDAC1, with a selectivity factor of 2.5. Complexes **8f** and **L^1^** showed slightly reduced activity compared to SAHA, with selectivity close to that of the control compound. Of all compounds tested, complex **1** displayed the largest selectivity factor of 7.5, a three-fold improvement in selectivity compared to the ligand **L^1^** alone. For complex **1**, HDAC6 inhibition was very close to that of **8f** and **L^1^**, but HDAC1 activity was reduced, leading to the observed selectivity. HDAC1 has a narrower entrance to the catalytic channel, and the bulky *p*-cymene capping arene in complex **1** may restrict access to the channel. This result demonstrates how addition of the Ru metal complex can impart HDAC isoform selectivity compared to the purely organic ligand.

### 2.3. In Vitro Assays

To further investigate the biological activities of the complexes, studies were carried out into the in vitro cytotoxicity, the pathway of cell uptake, and cell uptake quantification. Cell viability assays were carried out in triplicate, using the MCF7 human breast adenocarcinoma cell line as a representative cancer cell line, and effective concentration (EC_50_) values were calculated for complexes **1** and **8a**–**f**, alongside control compounds **L^1^** and SAHA (Table 2).

The majority of the Ru complexes showed cytotoxicity equalling that of the clinically used inhibitor, SAHA. Variation of the capping arene in the Ru complexes does not appear to give any definitive trend in cytotoxicity. However, it can be noted that the EC_50_ value for **L^1^** is double that of SAHA, whereas the presence of the metal-arene in complexes **1**, **8b**, **8c** and **8d** slightly improves activity and provides EC_50_ values closer to the SAHA control. Furthermore, complex **1** shows variable behaviour depending upon cell line. Against MCF7 cells, complex **1** and SAHA have EC_50_ values of approximately 1.5 μM. Against H460 non-small lung carcinoma cells, complex **1** and SAHA returned EC_50_ values of 21 ± 6 μM and 1.5 ± 0.4 μM, respectively [32]. The origin of the selectivity of complex **1** is unclear, but the varying behaviour compared to SAHA could imply an alternative mechanism of cell uptake and/or mode of action. Indeed, Ru complexes are known to exert antiproliferative activity via several different modes of action [35,36,37].

Inductively coupled plasma mass spectrometry (ICP-MS) was employed to quantify intracellular Ru and discern any relationship between toxicity and cellular accumulation (Table 2).

The data show a clear trend between cellular uptake and toxicity, with the most potent Ru complexes (**8b**, **8c** and **8d**) showing the highest cellular uptake (18.9%, 13.8% and 13.1%, respectively). Conversely, complex **8a** with the lowest toxicity showed only 0.5% cellular uptake. It can be concluded that variation in toxicity between the complexes likely arises from the variation in cell uptake, although the reason for this variation is not clear.

Finally, through a cell assay with complex **1** at 4 °C [38,39], it was determined that an active transport mechanism of cell uptake is responsible for Ru complex ingress, as cells treated with **1** remained viable at this temperature, suggesting no Ru uptake (see Supplementary Materials for details).

### 2.4. Computational Docking Study

In an attempt to rationalise variation in HDAC-inhibitory activity, docking studies were carried out for SAHA, **L^1^**, **1**, **8c** and **8f** against HDAC1 (hHDAC1, 4bkx) and HDAC6-CD2 (hHDAC6-CD2, 5edu, see Supplementary Materials for details). Comparative analysis of the outputs was conducted, based on: (a) the docking ChemScore rankings (a prediction of total free energy change upon inhibitor binding to protein); (b) the number of docking poses (out of the 10 generated) showing zinc-hydroxamic acid coordination, and (c) the zinc-coordination mode (monodentate vs. bidentate). Previously solved crystal structures typically show hydroxamic acid-based HDAC inhibitors with bidentate coordination of the hydroxamic acid to zinc [33,40], with a few examples showing monodentate coordination with a secondary bridging water molecule coordination [41].

**Figure 3:**
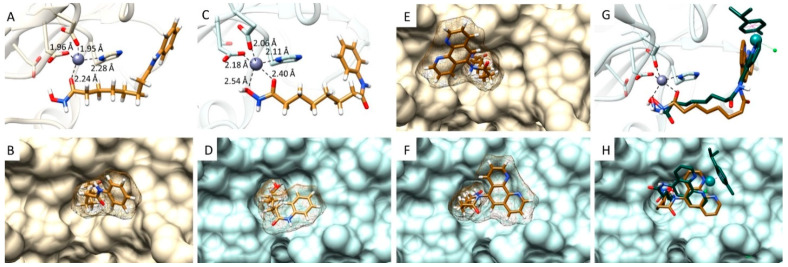
Computational docking studies showing (**A**) Zn(II) coordination view of SAHA with HDAC1; (**B**) active site cavity entrance surface view of SAHA with HDAC1; (**C**) Zn(II) coordination view of SAHA with HDAC6; (**D**) active site cavity entrance surface view of SAHA with HDAC6; (**E**) active site cavity entrance surface view of **L^1^** with HDAC1; (**F**) active site cavity entrance surface view of **L^1^** with HDAC6; (**G**) Zn(II) coordination view of **L^1^** (gold) and **1** (teal) overlapped with HDAC6; (**H**) active site cavity entrance surface view of **L^1^** and **1** overlapped with HDAC6.

From the 10 docking predictions made between SAHA and HDAC1, only 1/10 predicted zinc-hydroxamic acid binding (monodentate binding, Figure 3A,B), while docking between SAHA and HDAC6 showed 9/10 poses with Zn-hydroxamic acid binding, comprised of 2 monodentate and 7 bidentate (Figure 3C,D). ChemScore values of 17.1 and 23.7 were calculated against HDAC1 and HDAC6, respectively. This follows the experiment results, which showed a preference of SAHA to HDAC6 over HDAC1, validating the docking parameters.

Ligand **L^1^** also appeared to favour HDAC6 over HDAC1, with all 10 docking positions binding to the zinc in the former, compared to 8/10 for the latter. The ChemScore was also 25.9 vs. 23.7 for HDAC6 vs. HDAC1. The surface view of the docking shows that in HDAC1, the phenanthroline moiety extends out of the cavity entrance (Figure 3E), whereas for HDAC6 the phenanthroline moiety fits more comfortably in the wider cavity entrance (Figure 3F). Similarly, complex **1** returned a higher ChemScore against HDAC6, relative to HDAC1. Overlapping the highest scoring docking for **L^1^** and **1** shows very similar binding, with the Ru complex benefiting from additional interaction between the *p*-cymene capping arene and a hydrophobic cleft on the surface of HDAC6 (Figure 3G,H and Figure S22).

Experimental data suggests that the η^6^-tolyl capped complexes show increased HDAC6 activity, compared to the η^6^-phenyl capped complexes. In docking studies (Figures S18–S20), the ChemScore was higher for **8f** than **8c** (19.1 vs. 13.6) against HDAC6, which reflects the increase in binding affinity for **8f**. While docking studies are used with caution, this preliminary study does suggest that the wider cavity entrance of HDAC6 is able to accommodation the larger **L^1^** and Ru complexes better than the narrower HDAC1 cavity entrance. This is reflected in the IC_50_ inhibition assay experimental data, where HDAC6 inhibition is more efficient.

## 3. Conclusions

In conclusion, six novel Ru piano stool complexes have been synthesised as inhibitors of the HDAC enzymes. The novel complexes show excellent in vitro anticancer activity, which correlates with the extent of cell-uptake. All complexes are able to inhibit HDAC1 and HDAC6, and variation in inhibitory potency arises from variation in the capping arene of the piano stool complex. Varying the capping η^6^-arene from phenyl-derived (**8a**–**c**) to tolyl-derived (**8d**–**f**) leads to an increase in HDAC6 inhibition. Comparing the HDAC activity and selectivity of the ligand **L^1^** and the Ru complex **1** shows that, while addition of the transition metal complex does not increase overall potency, it does have a beneficial effect on selectivity between the two isoforms. Preliminary docking studies provide some evidence for this variation and will be used in future work as a guide for designing inhibitors with increased potency and selectivity. Overall, this proof of concept study highlights the potential for organometallic complexes to address the challenge of isoform-selective HDAC inhibitors.

## Figures and Tables

**Table 1:** IC_50_ values measured for selected compounds against HDAC1 and HDAC6. Selectivity factor defined as ratio of the two IC_50_ values. All assays carried out in triplicate. See Supplementary Materials for details.

Compound	HDAC1 IC_50_ (nM)	HDAC6 IC_50_ (nM)	Selectivity Factor
**1**	240 ± 30	32 ± 1	7.5
**8f**	80 ± 10	35 ± 6	2.3
**L^1^**	105 ± 15	41 ± 2	2.6
SAHA	30 ± 4	12 ± 1	2.5

**Table 2:** In vitro EC_50_ cell anticancer activity and cellular uptake measured against the MCF7 human breast adenocarcinoma cell line (96 h, using ChemoMetec Via1-Cassette assay). All assays carried out in triplicate. See Supplementary Materials for details.

Compound	EC_50_ (μM)	Cellular Uptake (%)	Compound	EC_50_ (μM)	Cellular Uptake (%)
SAHA	1.5 ± 0.2	-	**8d**	1.2 ± 0.3	13.1
**1**	1.5 ± 0.4	8.0	**8e**	5.1 ± 1.5	6.2
**8a**	32 ± 2	0.5	**8f**	2.1 ± 0.2	11.2
**8b**	1.4 ± 0.2	18.9	**L^1^**	3.0 ± 0.6	-
**8c**	1.7 ± 0.3	13.8			

## Supplementary Materials

The following are available online: synthetic procedures; hydrolysis studies; details on HPLC purification and analysis; enzyme inhibition assay; cell viability assays; cell uptake assays; computational docking studies; NMR spectra.
