# Supplementary material for: Anticancer Ruthenium Complexes with HDAC Isoform Selectivity
Source: Molecules. 2020 May 21;25(10):2383. doi: 10.3390/molecules25102383 (PMC7287671; doi:10.3390/molecules25102383)
Supplement: Supplementary file 1 [file molecules-25-02383-s001.pdf]

# Supplementary Material

Anticancer Ruthenium Complexes with HDAC Isoform Selectivity

Jasmine M. Cross, Tim R. Blower, Alexander D. H. Kingdon, Robert Pal, David M. Picton, James W. Walton*

## Contents

1. General Information....S1
2. Synthetic Procedures ....S2
3. Aqueous Stability Assay....S17
4. HPLC Purification and Analysis ....S18
5. Enzyme Inhibition Assay....S19
6. Cell Viability Assays ....S23
7. Cell Uptake Assay ....S24
8. Computational Docking Studies....S26
9. NMR Spectra ....S36
10. HPLC Traces....S54
11. References....S56

## 1. General Information

Chemicals were purchased from Sigma Aldrich UK, Fluorochem, Merck and Fisher UK. Solvents were laboratory grade or dried by the Durham University SPS service. Dried solvents were stored over activated 3 Å molecular sieves. Reactions requiring anhydrous conditions were carried out under an atmosphere of dry argon or nitrogen using Schlenk-line techniques. Where appropriate, solvents were sparged with argon as a degas method.

Thin-layer chromatography was carried out on silica plates (Merck 5554) and visualised under UV (254/365 nm) irradiation or by staining with iodine, vanillin or potassium permanganate stains. Preparative column chromatography was carried out using silica (Merck Silica Gel 60, 230400 mesh).

NMR spectra ($^1H$, $^{13}C$) were recorded on a Varian VXR-400 spectrometer ($^1H$ at 399.97 Hz, $^{13}C$ at 100.57 MHz) or a Varian VNMRS-700 spectrometer ($^1H$ at 699.73 MHz, $^{13}C$ at 175.95 MHz). Spectra were recorded at 295 K in commercially available deuterated solvents and referenced internally to the residual solvent resonances. The multiplicity of each signal is indicated by s (singlet); d (doublet); t (triplet); q (quartet); quin (quintet) or sept (septet). The number of protons (n) for a given resonance signal is indicated by nH. Coupling constants (*J*) are quoted in Hz and are recorded to the nearest 0.1 Hz. Identical proton coupling constants (*J*) are averaged in each spectrum and reported to the nearest 0.1 Hz. The coupling constants are determined by analysis using MestreNova software. Spectra were assigned using COSY, HSQC, HMBC and NOESY experiments as necessary.

Both electrospray and high-resolution mass spectrometry were performed on a Thermo-Finnigan LTQ FT system using methanol as the carrier solvent. *m/z* values are reported in Daltons with specific isotopes identified.

## 2. Synthetic Procedures

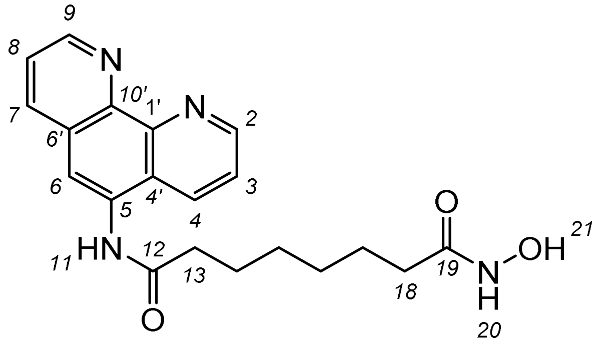


*$N^{20}$-Hydroxy-$N^{11}$-(1,10-phenanthrolin-5-yl)octanediamide* $\boldsymbol{L^1}$

1,10-Phenanthrolin-5-amine (0.100 g, 0.51 mmol), methyl 8-chloro-8-oxooctanoate (0.158 g, 0.76 mmol) and 4-dimethylaminopyridine (0.01 g, 0.051 mmol) were dissolved in DMF (7 mL) and stirred for 16 h at room temperature under an inert atmosphere. The volume of solution was reduced by 70% and added dropwise to a stirring solution of diethyl ether (10 mL). The filtrate was decanted and the precipitate dried under vacuum. The solid was suspended in MeOH : hydroxylamine solution 50% (5 mL) and 1M NaOH solution was added (0.5 mL) with stirring for 30 min. The solution was neutralised to pH 7 and a precipitate formed on standing. The filtrate was decanted and the resulting solid dried under vacuum to give a beige solid (0.061 g, 32%). $\boldsymbol{\delta_H}$ (DMSO-$d_6$) 10.3 (1H, s, $H^{21}$), 10.1 (1H, s, $H^{11}$), 9.09 (1H, dd, $^3J_{HH}$ 4.3 Hz $^4J_{HH}$ 1.4 Hz, $H^2$), 9.00 (1H, dd, $^3J_{HH}$ 4.1 Hz $^4J_{HH}$ 1.1 Hz, $H^9$), 8.68 (1H, s, $H^{20}$), 8.56 (1H, dd, $^3J_{HH}$ 8.0 Hz $^4J_{HH}$ 1.4 Hz, $H^4$), 8.41 (1H, dd, $^3J_{HH}$ 7.7 Hz $^4J_{HH}$ 1.1Hz, $H^7$), 8.13 (1H, s, $H^6$), 7.80 (1H, dd, $^3J_{HH}$ 8.0 Hz $^3J_{HH}$ 4.3 Hz, $H^3$), 7.70 (1H, dd, $^3J_{HH}$ 7.7 Hz $^3J_{HH}$ 4.5 Hz, $H^8$), 2.47 (2H, t, $^3J_{HH}$ 7.5 Hz, $H^{13}$), 1.93 (2H, t, $^3J_{HH}$ 7.5 Hz, $H^{18}$), 1.65 (2H, quin, $^3J_{HH}$ 7.5 Hz, $H^{14}$), 1.50 (2H, quin, $^3J_{HH}$ 7.5

Hz, $H^{17}$), 1.35 (2H, m, $H^{15}$), 1.29 (2H, m, $H^{16}$); **δ**$_C$ (DMSO-$d_6$) 172.8 ($C^{12}$), 169.5 ($C^{19}$), 150.2 ($C^2$), 149.7 ($C^9$), 146.3 ($C^{1'}$), 144.2 ($C^{10'}$), 136.2 ($C^4$), 132.2 ($C^5$), 132.0 ($C^7$), 128.5 ($C^{6'}$), 125.1 ($C^{4'}$), 123.9 ($C^8$), 123.2 ($C^3$), 120.5 ($C^6$), 36.4 ($C^{13}$), 32.7 ($C^{18}$), 28.9 ($C^{15}$), 28.8 ($C^{16}$), 25.6 ($C^{14}$), 25.5 ($C^{17}$); *m/z* (HRMS$^+$) 367.1764 $[M + H]^+$ ($C_{20}H_{23}N_4O_3$ requires 367.1770).

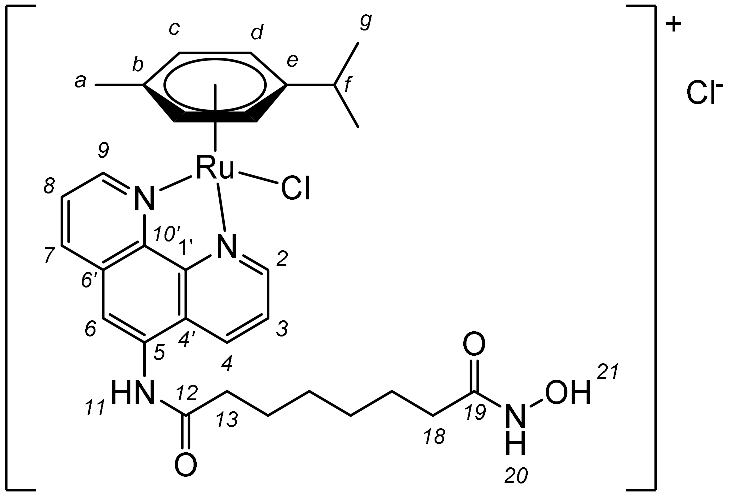


*Chloro($\eta^6$-p-cymene)(**$L^1$**) ruthenium(II) chloride* **1**

***$L^1$*** (0.031 g, 0.084 mmol) and dichloro(*p*-cymene) ruthenium(II) dimer (0.025 g, 0.042 mmol) were suspended in anhydrous MeOH (5 mL) and stirred for 16 h at 40 °C under an inert atmosphere. The solvent volume was reduced to 1 mL and dropped into cold diethyl ether (3 mL). The resulting precipitate was filtered to give the title compounds as a yellow solid (0.032 g, 58%). **δ**$_H$ (DMSO-$d_6$) 10.50 (1H, s, $H^{11}$), 10.30 (1H, s, $H^{21}$), 9.94 (1H, d, $^3J_{HH}$ 5.0 Hz, $H^2$), 9.81 (1H, d, $^3J_{HH}$ 5.0 Hz, $H^9$), 9.05 (1H, d, $^3J_{HH}$ 8.6 Hz, $H^4$), 8.86 (1H, d, $^3J_{HH}$ 6.5 Hz, $H^7$), 8.63 (1H, s, $H^{20}$), 8.50 (1H, s, $H^6$), 8.18 (1H, dd, $^3J_{HH}$ 8.6 $^3J_{HH}$ 5.0 Hz, $H^3$), 8.08 (1H, dd, $^3J_{HH}$ 8.0 Hz $^3J_{HH}$ 5.0 Hz, $H^8$), 6.34 (1H, d, $^3J_{HH}$ 5.5 Hz, $H^d$), 6.31 (1H, d, $^3J_{HH}$ 5.5 Hz, $H^{d'}$), 6.11 (1H, d, $^3J_{HH}$ 5.5 Hz, $H^c$), 6.08 (1H, d, $^3J_{HH}$ 5.5 Hz, $H^{c'}$), 2.59 (1H, septet, $^3J_{HH}$ 6.7 Hz, $H^f$), 2.56 (2H, t, $^3J_{HH}$ 7.5 Hz, $H^{13}$), 2.15 (3H, s, $H^a$), 1.94 (2H, t, $^3J_{HH}$ 7.5 Hz, $H^{18}$), 1.66 (2H, quin, $^3J_{HH}$ 7.5 Hz, $H^{14}$), 1.50 (2H, quin, $^3J_{HH}$ 7.5 Hz, $H^{17}$), 1.36-1.28 (4H, m, $H^{15}$,$H^{16}$), 0.89 (3H, d, $^3J_{HH}$ 5.5 Hz, $H^g$), 0.88 (3H, d, $^3J_{HH}$ 5.5 Hz, $H^{g'}$); **δ**$_C$ (DMSO-$d_6$) 173.2 ($C^{12}$), 169.5 ($C^{19}$), 156.5 ($C^2$), 155.2 ($C^9$), 145.8 ($C^{1'}$), 143.2 ($C^{10'}$), 138.6 ($C^7$), 135.3 ($C^4$), 133.8 ($C^5$), 130.1 ($C^{6'}$), 126.8 ($C^8$), 126.5 ($C^{4'}$), 126.1 ($C^3$), 119.1 ($C^6$), 104.5 ($C^e$), 103.1 ($C^b$), 86.5 ($C^{d'}$), 86.3 ($C^d$), 84.4 ($C^{c'}$), 84.2 ($C^c$), 36.4 ($C^{13}$), 32.7 ($C^{18}$), 30.8 ($C^f$), 28.9 ($C^{15}$), 28.8 ($C^{16}$), 25.5 ($C^{17}$), 25.4 ($C^{14}$), 22.1 ($C^g$), 22.0 ($C^{g'}$), 18.6 ($C^a$); *m/z* (HRMS$^+$) 612.1581 $[M\text{-}2Cl\text{+}OH]^+$ ($C_{30}H_{36}N_4O_4{}^{96}Ru$ requires 612.1603). Anal. Calcd for $C_{30}H_{36}Cl_2N_4O_3Ru.(H_2O)2.5$ : C, 50.20; H, 5.05; N, 7.80. Found: C, 50.07; H, 5.11; N, 7.69.

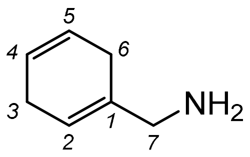


*(Cyclohexa-1,4-dienyl)methylamine* ***4*** ***reference: 10.1039/J39710002580***

Benzylamine (3.00 g, 27.9 mmol) was added to liquid ammonia (15 ml), precooled to –78 °C. Sodium (1.60 g, 69.9 mmol) was added in small portions and the mixture stirred for 2 h. The reaction was quenched by the addition of methanol (10 ml) before the mixture was warmed to room temperature to remove excess ammonia. The volume of the reaction mixture was reduced to around 2 ml, before water (15 ml) was added and the mixture extracted with $Et_2O$ (3 × 15 ml). The organic layers were combined, dried over $MgSO_4$, filtered and the solvent removed under reduced pressure to give the title compound as a crude yellow oil, which was used without further purification (1.60 g, 53%). $\delta_H$ ($CDCl_3$) 5.79 – 5.69 (2H, m, $H^4$ and $H^5$), 5.63 – 5.68 (1H, m, $H^2$), 3.20 (2H, s, $H^7$), 2.77 – 2.62 (4H, m, $H^3$ and $H^6$).

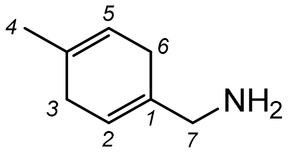


*(4-methylcyclohexa-1,4-dien-1-yl)methanamine* ***5***

*p*-Toluidine (2.00 g, 16.2 mmol) was added to liquid ammonia (15 ml), precooled to –78 °C. Sodium (1.30 g, 56.8 mmol) was added in small portions and the mixture stirred for 2 h. The reaction was quenched by the addition of methanol (7 ml) before the mixture was warmed to room temperature to remove excess ammonia. The volume of the reaction mixture was reduced to around 2 ml, before water (15 ml) was added and the mixture extracted with $Et_2O$ (3 × 15 ml). The organic layers were combined, dried over $MgSO_4$, filtered and the solvent removed under reduced pressure to give the title compound as a crude yellow oil, which was used without further purification (1.37 g, 45%). $\delta_H$ ($CDCl_3$) 5.63 – 5.59 (1H, m, $H^2$), 5.48 – 5.44 (1H, m, $H^5$), 3.21 (2H, s, $H^7$), 2.70 – 2.58 (4H, m, $H^3$ and $H^6$), 1.40 (3H, s, $H^4$).

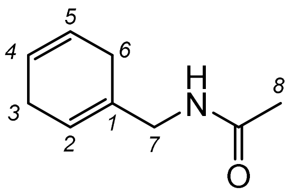

*(Cyclohexa-1,4-dien-1-ylmethyl)acetamide* ***6a***

(Cyclohexa-1,4-dienyl)methylamine (100 mg, 0.917 mmol) and acetyl chloride (86.0 mg, 1.10 mmol) were added to anhydrous $CH_2Cl_2$ (5 ml). Triethylamine (185 mg, 1.83 mmol) was added and the mixture stirred at 0 °C for 1 h, before the reaction was allowed to reach room temperature and stirred for a further 16 h. Additional $CH_2Cl_2$ was added (10 ml) and the mixture was washed with aqueous ammonium chloride solution (5% w/w, 3 × 10 ml) and brine (3 × 10 ml). The organic layer was dried over $Na_2SO_4$, filtered and the solvent removed under reduced pressure to the title compound as a crude yellow oil, which was used without further purification (132 mg, 95%). $\boldsymbol{\delta_H}$ ($CDCl_3$) 5.77 – 5.67 (2H, m, $H^4$ and $H^5$), 5.64 – 5.59 (1H, m, $H^2$), 3.80 (2H, d, $^3J_{HH}$ 5.0 Hz, $H^7$), 2.79 – 2.58 (4H, m, $H^3$ and $H^6$), 2.03 (3H, s, $H^8$).

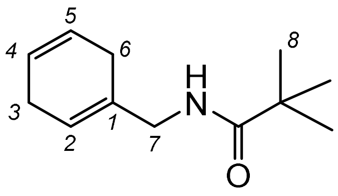

*N-[(Cyclohexa-1,4-dien-1-yl)methyl]-2,2-dimethylpropanamide* ***6b***

(Cyclohexa-1,4-dienyl)methylamine (200 mg, 1.83 mmol) and 2,2-dimethylpropanoyl chloride (265 mg, 2.20 mmol) were added to anhydrous $CH_2Cl_2$ (10 ml). Triethylamine (370 mg, 3.66 mmol) was added and the mixture stirred at 0 °C for 1 h, before the reaction was allowed to reach room temperature and stirred for a further 16 h. Additional $CH_2Cl_2$ was added (15 ml) and the mixture was washed with aqueous ammonium chloride solution (5% w/w, 3 × 15 ml) and brine (3 × 15 ml). The organic layer was dried over $Na_2SO_4$, filtered and the solvent removed under reduced pressure to the title compound as a crude yellow oil, which was used without further purification (110 mg, 31%). $\boldsymbol{\delta_H}$ ($CDCl_3$) 5.77 – 5.68 (2H, m, $H^4$ and $H^5$), 5.61 – 5.56 (1H, m, $H^2$), 3.82 (2H, d, $^3J_{HH}$ 5.0 Hz, $H^7$), 2.77 – 2.57 (4H, m, $H^3$ and $H^6$), 1.24 (9H, s, $H^8$).

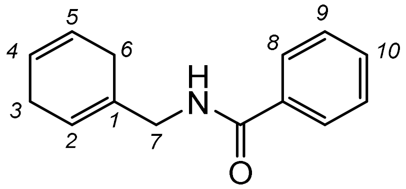


*N-[(Cyclohexa-1,4-dien-1-yl)methyl]benzamide* ***6c***

(Cyclohexa-1,4-dienyl)methylamine (250 mg, 2.29 mmol) and benzoyl chloride (483 mg, 3.44 mmol) were added to anhydrous $CH_2Cl_2$ (8 ml). Triethylamine (463 mg, 4.58 mmol) was added and the mixture stirred at 0 °C for 1 h, before the reaction was allowed to reach room temperature and stirred for a further 16 h. Additional $CH_2Cl_2$ was added (15 ml) and the mixture was washed with aqueous ammonium chloride solution (5% w/w, 3 × 15 ml) and brine (3 × 15 ml). The organic layer was dried over $Na_2SO_4$, filtered and the solvent removed under reduced pressure to the title compound as a crude yellow oil, which was used without further purification (181 mg, 37%). $\boldsymbol{\delta}_{\mathbf{H}}$ ($CDCl_3$) 8.15 – 8.11 (2H, m, $H^8$), 7.66 – 7.60 (1H, m, $H^{10}$), 7.53 – 7.49 (2H, m, $H^9$), 5.79 – 5.72 (2H, m, $H^4$ and $H^5$), 5.72 – 5.67 (1H, m, $H^2$), 4.04 (2H, d, $^3J_{HH}$ 5.0 Hz, $H^7$), 2.80 – 2.66 (4H, m, $H^3$ and $H^6$).

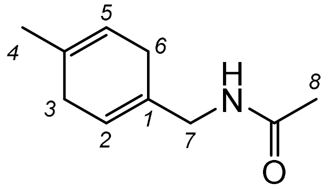


*N-[(4-Methylcyclohexa-1,4-dien-1-yl)methyl]acetamide* ***6d***

(4-Methylcyclohexa-1,4-dien-1-yl)methanamine (250 mg, 2.01 mmol) and acetyl chloride (190 mg, 2.42 mmol) were added to anhydrous $CH_2Cl_2$ (15 ml). Triethylamine (407 mg, 4.03 mmol) was added and the mixture stirred at 0 °C for 1 h, before the reaction was allowed to reach room temperature and stirred for a further 16 h. Additional $CH_2Cl_2$ was added (10 ml) and the mixture was washed with aqueous ammonium chloride solution (5% w/w, 3 × 15 ml) and brine (3 × 15 ml). The organic layer was dried over $Na_2SO_4$, filtered and the solvent removed under reduced pressure to the title compound as a crude yellow oil, which was used without further purification (145 mg, 44%). $\boldsymbol{\delta}_{\mathbf{H}}$ ($CDCl_3$) 5.62 – 5.58 (1H, m, $H^2$), 5.44 – 5.39 (1H, m, $H^5$), 3.79 (2H, d, $^3J_{HH}$ 5.0 Hz, $H^7$), 2.63 – 2.57 (4H, m, $H^3$ and $H^6$), 2.00 (3H, s, $H^8$), 1.68 (3H, s, $H^4$).

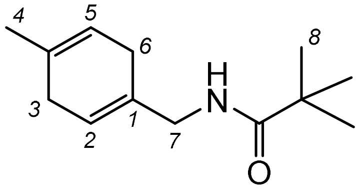


*2,2-Dimethyl-N-[4-methyl(cyclohexa-1,4-dien-1-yl)methyl]propanamide* ***6e***

(4-Methylcyclohexa-1,4-dien-1-yl)methanamine (250 mg, 2.01 mmol) and 2,2-dimethylpropanoyl chloride (292 mg, 2.42 mmol) were added to anhydrous $CH_2Cl_2$ (15 ml). Triethylamine (407 mg, 4.03 mmol) was added and the mixture stirred at 0 °C for 1 h, before the reaction was allowed to reach room temperature and stirred for a further 16 h. Additional $CH_2Cl_2$ was added (10 ml) and the mixture was washed with aqueous ammonium chloride solution (5% w/w, 3 × 15 ml) and brine (3 × 15 ml). The organic layer was dried over $Na_2SO_4$, filtered and the solvent removed under reduced pressure to the title compound as a crude yellow oil, which was used without further purification (87 mg, 21%). $\delta_H$ ($CDCl_3$) 5.61 – 5.57 (1H, m, $H^2$), 5.46 – 5.39 (1H, m, $H^5$), 3.83 (2H, d, $^3J_{HH}$ 5.0 Hz, $H^7$), 2.66 – 2.58 (4H, m, $H^3$ and $H^6$), 1.70 (3H, s, $H^4$), 1.23 (9H, s, $H^8$).

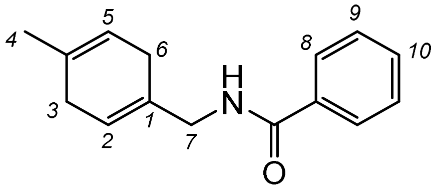


*N-[(4-Methylcyclohexa-1.4-dien-1-yl)methyl]benzamide* ***6f***

(4-Methylcyclohexa-1,4-dien-1-yl)methanamine (200 mg, 1.62 mmol) and benzoyl chloride (411 mg, 2.92 mmol) were added to anhydrous $CH_2Cl_2$ (10 ml). Triethylamine (328 mg, 3.25 mmol) was added and the mixture stirred at 0 °C for 1 h, before the reaction was allowed to reach room temperature and stirred for a further 16 h. Additional $CH_2Cl_2$ was added (10 ml) and the mixture was washed with aqueous ammonium chloride solution (5% w/w, 3 × 15 ml) and brine (3 × 15 ml). The organic layer was dried over $Na_2SO_4$, filtered and the solvent removed under reduced pressure to the title compound as a crude yellow oil, which was used without further purification (87 mg, 24%). $\delta_H$ ($CDCl_3$) 8.21 – 8.17 (2H, m, $H^8$), 7.74 – 7.68 (1H, m, $H^{10}$), 7.59 – 7.52 (2H, m, $H^9$), 5.72 – 5.67 (1H, m, $H^2$), 5.49 – 5.43 (1H, m, $H^5$), 4.04 (2H, d, $^3J_{HH}$ 5.0 Hz, $H^7$), 2.76 – 2.59 (4H, m, $H^3$ and $H^6$), 1.70 (3H, s, $H^4$).

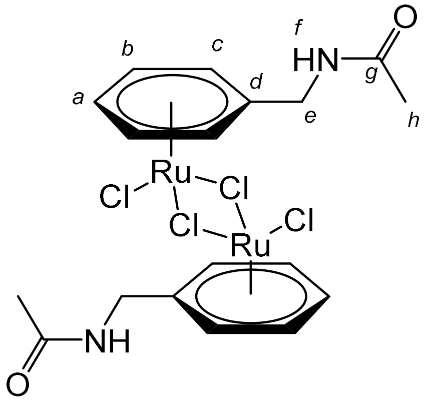


*Dichloro(η^6-N-acetylbenzylamide) ruthenium(II) dimer* **7a**

*N-[(Cyclohexa-1,4-dien-1-yl)methyl]acetamide* (0.149 g, 0.986 mmol) and ruthenium(III) chloride hydrate (0.128 g, 0.493mmol) were suspended in degassed EtOH (10 mL) and heated to 80 °C for 16 h under an argon atmosphere. The solution was then cooled in an ice bath for 2 h. The resulting precipitate was filtered and washed with cold EtOH (5 mL), diethylether (5 mL) and dried under vacuum to provide the *title compound* as a yellow-brown precipitate (0.070 g, 17%). $\boldsymbol{\delta}_{\mathbf{H}}$ (DMSO-d$_6$) 8.31 (2H, t, $^3J_{HH}$ 5.8 Hz, H$^f$), 6.02 (4H, t, $^3J_{HH}$ 5.8 Hz, H$^b$), 5.78 (4H, d, $^3J_{HH}$ 5.8 Hz, H$^c$), 5.75 (2H, t, $^3J_{HH}$ 5.8 Hz, H$^a$), 4.08 (4H, d, $^3J_{HH}$ 5.8 Hz, H$^e$), 1.84 (6H, s, H$^h$); $\boldsymbol{\delta}_{\mathbf{C}}$ (DMSO-d$_6$) 170.3 (2C, C$^g$), 103.4 (2C, C$^d$), 89.0 (4C, C$^b$), 85.4 (4C, C$^c$), 84.4 (2C, C$^a$), 40.5 (2C, C$^e$), 22.8 (2C, C$^h$); *m/z* (ASAP)$^+$ 612.8804 [M-Cl]$^+$ ([$C_{18}H_{22}N_2O_2{}^{35}Cl^{37}Cl_2{}^{104}Ru^{102}Ru$]$^+$ requires 612.8807).

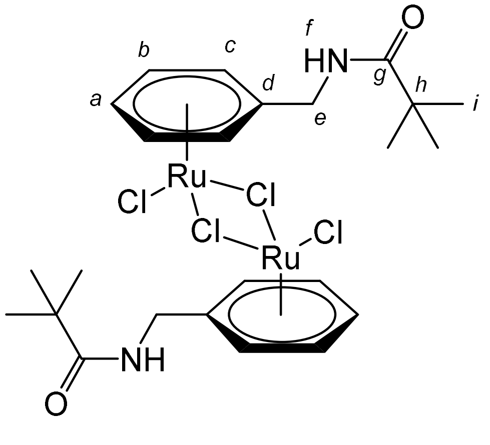


*Dichloro(η^6-N-trimethylacetylbenzylamide) ruthenium(II) dimer* **7b**

*N-[(Cyclohexa-1,4-dien-1-yl)methyl]-2,2-dimethylpropanamide* (0.110 g, 0.569 mmol) and ruthenium(III) chloride hydrate (0.074 g, 0.284 mmol) were suspended in degassed EtOH (10 mL) and heated to 80 °C for 16 h under an argon atmosphere. The solution was then cooled in an ice bath for 2 h. The resulting precipitate was filtered and washed with cold EtOH (5 mL), diethylether (5 mL) and

dried under vacuum to provide the *title compound* as a brown precipitate (0.064 g, 25%). **δ$_H$** (DMSO-$d_6$) 7.93 (2H, t, $^3J_{HH}$ 5.4 Hz, $H^f$), 6.04 (4H, t, $^3J_{HH}$ 6.9 Hz, $H^b$), 5.79-5.77 (6H, m, $H^a$ $H^c$), 4.12 (2H, d, $^3J_{HH}$ 5.4 Hz, $H^e$), 1.09 (18H, s, $H^i$); **δ$_C$** (DMSO-$d_6$) 178.4 (2C, $C^g$), 103.8 (2C, $C^d$), 88.8 (4C, $C^b$), 85.5 (4C, $C^c$), 84.7 (2C, $C^a$), 40.8 (2C, $C^e$), 38.55 (2C, $C^h$), 27.7 (6C, $C^i$); *m/z* (ASAP)$^+$ 690.9734 [M-Cl]$^+$ ([$C_{24}H_{34}N_2O_2{}^{35}Cl_3{}^{102}Ru_2$]$^+$ requires 690.9767).

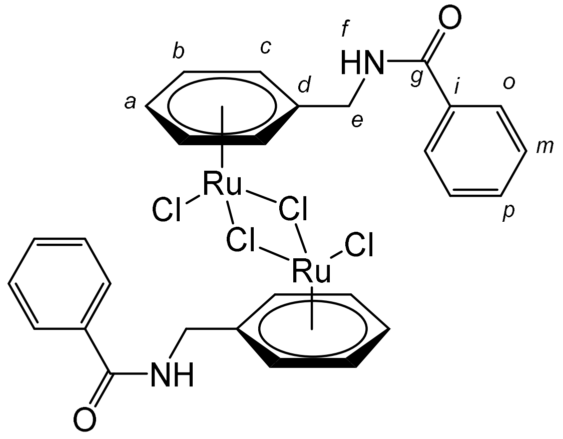


*Dichloro(N-benzoyl-η$^6$-benzylamide) ruthenium(II) dimer* **7c**

*N-[(Cyclohexa-1,4-dien-1-yl)methyl]benzamide* (0.181 g, 0.848 mmol) and ruthenium(III) chloride hydrate (0.110 g, 0.424 mmol) were suspended in degassed EtOH (10 mL) and heated to 80 °C for 16 h under an argon atmosphere. The solution was then cooled in an ice bath for 2 h. The resulting precipitate was filtered and washed with cold EtOH (5 mL), diethylether (5 mL) and dried under vacuum to provide the *title compound* as an orange-brown precipitate (0.128 g, 38%). **δ$_H$** (DMSO-$d_6$) 8.97 (2H, t, $^3J_{HH}$ 6.0 Hz, $H^f$), 7.85 (4H, dd, $^3J_{HH}$ 7.0 Hz $^4J_{HH}$ 1.3 Hz, $H^o$), 7.52 (2H, t, $^3J_{HH}$ 7.0 Hz, $H^p$), 7.45 (4H, t, $^3J_{HH}$ 7.0 Hz, $H^m$), 6.03 (4H, t, $^3J_{HH}$ 5.6 Hz, $H^b$), 5.91 (4H, d, $^3J_{HH}$ 5.6 Hz, $H^c$), 5.80 (2H, t, $^3J_{HH}$ 5.6 Hz, $H^a$), 4.31 (4H, d, $^3J_{HH}$ 6.0 Hz, $H^e$); **δ$_C$** (DMSO-$d_6$) 166.9 (2C, $C^g$), 134.0 (2C, $C^i$), 132.0 (2C, $C^p$), 128.8 (4C, $C^m$), 127.7 (4C, $C^o$), 102.7 (2C, $C^d$), 88.6 (4C, $C^b$), 86.4 (4C, $C^c$), 85.0 (2C, $C^a$), 41.4 (2C, $C^e$); *m/z* (ASAP)$^+$ 729.0135 [M-Cl]$^+$ ([$C_{28}H_{26}{}^{35}Cl_3N_2O_2{}^{101}Ru_2$]$^+$ requires 729.9156).

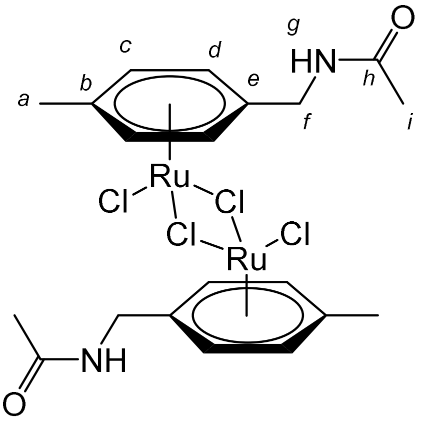


*Dichloro(η[6]-4-methyl-N-acetylbenzylamide) ruthenium(II) dimer* **7d**

*N-[(4-Methylcyclohexa-1,4-dien-1-yl)methyl]acetamide* (0.085 g, 0.514 mmol) and ruthenium(III) chloride hydrate (0.066 g, 0.257 mmol) were suspended in degassed EtOH (10 mL) and heated to 80 °C for 16 h under an argon atmosphere. The homogenous solution was cooled in an ice bath for 2 h. The resulting precipitate was filtered, and washed with cold EtOH (5 mL), diethylether (5 mL) and dried under vacuum to give the *title compound* (0.050 g, 29%) as a dark yellow solid; $\delta_H$ (DMSO-d$_6$) 8.19 (2H, t, $^3J_{HH}$ 5.6 Hz, H$^g$), 5.85 (4H, d, $^3J_{HH}$ 5.9 Hz, H$^d$), 5.78 (4H, d, $^3J_{HH}$ 5.9 Hz, H$^c$), 4.00 (4H, d, $^3J_{HH}$ 5.6 Hz, H$^f$), 2.06 (6H, s, H$^a$), 1.83 (6H, s, H$^i$); $\delta_C$ (DMSO-d$_6$) 170.2 (2C, C$^h$), 101.7 (2C, C$^b$), 96.8 (2C, C$^e$), 88.1 (4C, C$^d$), 86.5 (4C, C$^c$), 40.1 (2C, C$^f$), 22.8 (2C, C$^i$), 18.5 (2C, C$^a$); *m/z* (ASAP)$^+$ 634.9144 [M-Cl]$^+$ ([$C_{20}H_{26}{}^{37}Cl_3N_2O_2{}^{96}Ru{}^{101}Ru$]$^+$ requires 634.9145).

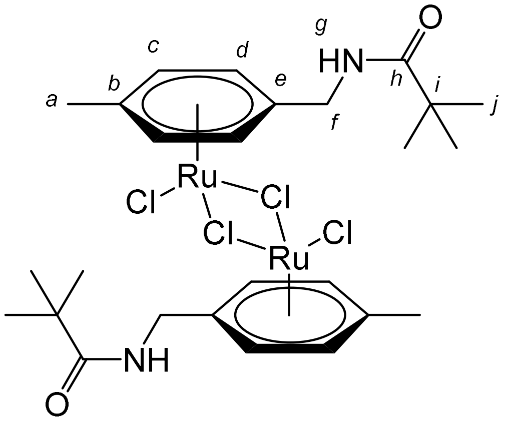


*Dichloro(η[6]-4-methyl-N-trimethylacetylbenzylamide) ruthenium(II) dimer* **7e**

*2,2-Dimethyl-N-[(4-methylcyclohexa-1,4-dien-1-yl)methyl]propanamide* (0.186 g, 0.895 mmol) and ruthenium(III) chloride hydrate (0.116 g, 0.447 mmol) were suspended in degassed EtOH (10 mL) and the reaction mixture was heated to 80 °C for 16 h under an argon atmosphere. The reaction was then cooled in an ice bath for 2 h. The resulting precipitate was filtered and washed with cold EtOH (5 mL), diethyl ether (5 mL) and dried under vacuum to give the *title compound* (0.092 g, 27%) as a dark orange

solid; $\delta_H$ (DMSO-d$_6$) 7.82 (2H, t, $^3J_{HH}$ 5.7 Hz, H$^g$), 5.84 (4H, d, $^3J_{HH}$ 6.0 Hz, H$^d$), 5.81 (4H, d, $^3J_{HH}$ 6.0 Hz, H$^c$), 4.05 (4H, d, $^3J_{HH}$ 5.7 Hz, H$^f$), 2.07 (6H, s, H$^a$), 1.08 (18H, s, H$^j$); $\delta_C$ (DMSO-d$_6$) 178.3 (2C, C$^h$), 102.0 (2C, C$^b$), 97.1 (2C, C$^e$), 88.1 (4C, C$^d$), 86.4 (4C, C$^c$), 40.3 (2C, C$^f$), 38.5 (2C, C$^i$), 27.7 (6C, C$^j$), 18.5 (2C, C$^a$); *m/z* (ASAP)$^+$ 719.0052 [M-Cl]$^+$ ([$C_{26}H_{38}{}^{35}Cl_3N_2O_2{}^{102}Ru_2$]$^+$ requires 719.0086).

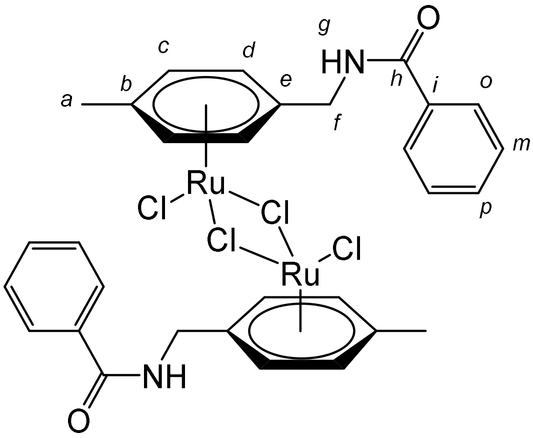


*Dichloro(η$^6$-4-methyl-N-benzoylbenzylamide) ruthenium(II) dimer* **7f**

*N-[(4-Methylcyclohexa-1.4-dien-1-yl)methyl]benzamide* (0.087 g, 0.383 mmol) and ruthenium(III) chloride hydrate (0.049 g, 0.191 mmol) were suspended in degassed EtOH (7 mL) and the reaction mixture was heated to 80 °C for 16 h under an argon atmosphere. The reaction was then cooled in an ice bath for 2 h. The resulting precipitate was filtered and washed with cold EtOH (5 mL), diethylether (5 mL) and dried under vacuum to give the *title compound* (0.068 g, 45%) as an orange solid; $\delta_H$ (DMSO-d$_6$) 8.87 (2H, t, $^3J_{HH}$ 5.6 Hz, H$^g$), 7.84 (4H, dd, $^3J_{HH}$ 7.7 Hz $^4J_{HH}$ 1.4 Hz, H$^o$), 7.52 (2H, t, $^3J_{HH}$ 7.7 Hz, H$^p$), 7.44 (4H, t, $^3J_{HH}$ 7.7 Hz, H$^m$), 5.98 (4H, d, $^3J_{HH}$ 6.2 Hz, H$^d$), 5.80 (4H, d, $^3J_{HH}$ 6.2 Hz, H$^c$), 4.24 (4H, d, $^3J_{HH}$ 5.6 Hz, H$^f$), 2.06 (6H, s, H$^a$); $\delta_C$ (DMSO-d$_6$) 166.9 (2C, C$^h$), 134.0 (2C, C$^i$), 132.0 (2C, C$^p$), 128.8 (4C, C$^m$), 127.7 (4C, C$^o$), 102.4 (2C, C$^b$), 95.9 (2C, C$^e$), 89.0 (4C, C$^d$), 86.1 (4C, C$^c$), 40.9 (2C, C$^f$), 18.5 (2C, C$^a$); *m/z* (HRMS)$^+$ 761.0339 [M-Cl]$^+$ ([$C_{30}H_{30}{}^{35}Cl_2{}^{37}ClN_2O_2{}^{99}Ru^{104}Ru$ requires 760.9457).

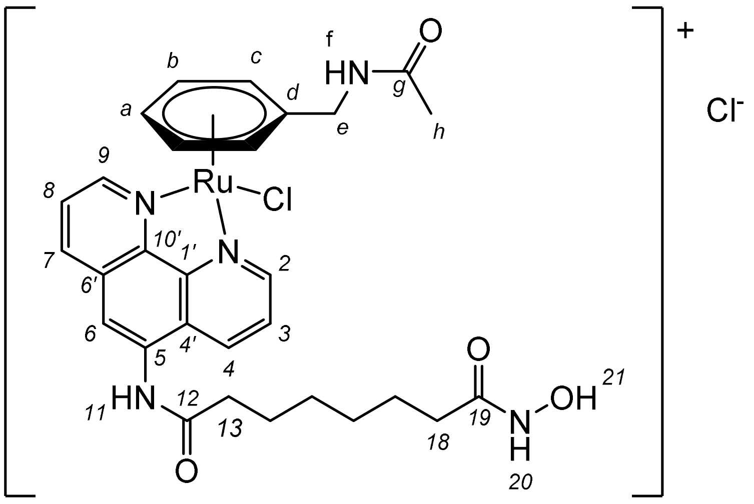


*Chloro($\eta^6$-N-acetylbenzylamide)($\boldsymbol{L^1}$) ruthenium(II) chloride* **8a**

$\boldsymbol{L^1}$ (0.025 g, 0.068 mmol) and **7a** (0.022 g, 0.034 mmol) were suspended in anhydrous MeOH (10 mL) and the solution was heated to 40 °C under an argon atmosphere, for 16 h. The volume of the solution was reduced to 1.5 mL and purified by reverse phase HPLC ($H_2O$ : MeOH) to yield the *title compound* (0.018 g, 76%) as a yellow solid; $\boldsymbol{\delta_H}$ (DMSO-$d_6$) 10.6 (1H, s, $H^{11}$), 10.4 (1H, s, $H^{21}$), 9.99 (1H, d, $^3J_{HH}$ 5.0 Hz, $H^2$), 9.86 (1H, d, $^3J_{HH}$ 5.5 Hz, $H^9$), 9.07 (1H, dd, $^3J_{HH}$ 8.4 Hz $^4J_{HH}$ 3.3 Hz, $H^4$), 8.83 (1H, d, $^3J_{HH}$ 8.1 Hz, $H^7$), 8.65 (1H, s, $H^{20}$), 8.52 (1H, t, $^3J_{HH}$ 5.8 Hz, $H^f$), 8.47 (1H, s, $H^6$), 8.16 (1H, dd, $^3J_{HH}$ 8.4 Hz $^3J_{HH}$ 5.0 Hz $H^3$), 8.06 (1H, dd, $^3J_{HH}$ 8.1 Hz $^3J_{HH}$ 5.5 Hz, $H^8$), 6.39 (1H, d, $^3J_{HH}$ 5.8 Hz, $H^c$), 6.38 (1H, d, $^3J_{HH}$ 6.0 Hz, $H^{c'}$) 6.26 (2H, t, $^3J_{HH}$ 5.9 Hz, $H^b$ $H^{b'}$), 5.96 (1H, t, $^3J_{HH}$ 5.9 Hz, $H^a$), 4.19 (2H, d, $^3J_{HH}$ 5.8 Hz, $H^e$), 2.57 (2H, t, $^3J_{HH}$ 6.5 Hz, $H^{13}$), 1.94 (2H, t, $^3J_{HH}$ 6.5 Hz, $H^{18}$), 1.75 (3H, s, $H^h$), 1.65 (2H, bm, $H^{14}$), 1.49 (2H, bm, $H^{17}$), 1.32-1.26 (4H, bm, $H^{15}$ $H^{16}$); $\boldsymbol{\delta_C}$ (DMSO-$d_6$) 173.2 ($C^{12}$), 170.2 ($C^g$), 169.5 ($C^{19}$), 156.6 ($C^2$), 155.4 ($C^9$), 146.1 ($C^{1'}$), 143.5 ($C^{10'}$), 138.5 ($C^7$), 135.4 ($C^4$), 133.8 ($C^{4'}$), 130.0 ($C^{6'}$), 126.7 ($C^8$), 126.6 ($C^5$), 125.9 ($C^3$), 119.0 ($C^6$), 102.7 ($C^d$), 87.6 (2C, $C^b$), 84.6 (2C, $C^c$), 84.4 ($C^a$), 40.4 ($C^e$), 36.4 ($C^{13}$), 32.7 ($C^{18}$), 28.9-28.8 ($C^{15}$ $C^{16}$), 25.5 ($C^{17}$), 25.4 ($C^{14}$), 22.7 ($C^h$); *m/z* ($HRMS^+$) 646.1320 $[M-Cl]^+$ ($C_{29}H_{33}N_5O_4{}^{35}Cl^{96}Ru$ requires 646.1297).

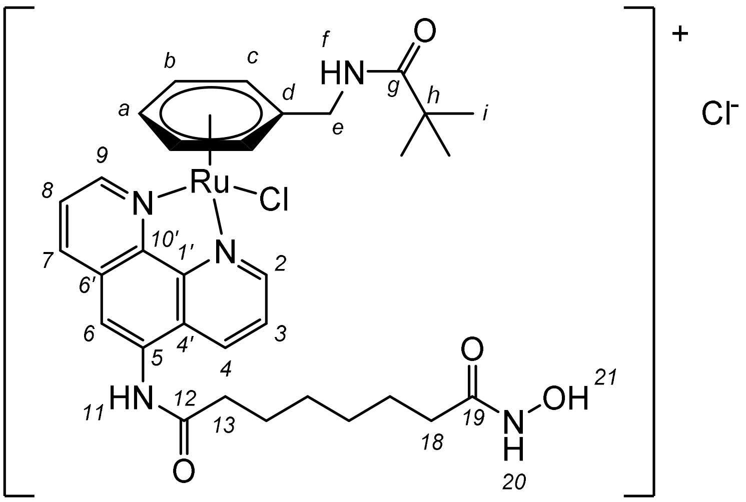


*Chloro(η$^6$-N-trimethylacetylbenzylamide)(**L$^1$**) ruthenium(II) chloride* **8b**

**7b** (0.035 g, 0.048 mmol) and ***L$^1$*** (0.035 g, 0.096 mmol) were suspended in anhydrous MeOH (10 mL) and the solution was heated to 40 °C under an argon atmosphere, for 16 h. The volume of the solution was reduced to 1.5 mL and purified by reverse phase HPLC ($H_2O$ : MeOH) to yield the *title compound* (0.046 g, 66%) as a dark yellow solid; $\delta_H$ (DMSO-d$_6$) 10.6 (1H, s, H$^{11}$), 10.4 (1H, s, H$^{21}$), 9.99 (1H, d, $^3J_{HH}$ 4.9 Hz, H$^2$), 9.85 (1H, d, $^3J_{HH}$ 5.0 Hz, H$^9$), 9.07 (1H, d, $^3J_{HH}$ 8.5 Hz, H$^4$), 8.82 (1H, d, $^3J_{HH}$ 8.3 Hz, H$^7$), 8.64 (1H, s, H$^{20}$), 8.47 (1H, s, H$^6$), 8.17-8.14 (2H, bm, H$^3$ H$^f$), 8.05 (1H, dd, $^3J_{HH}$ 8.3 Hz $^3J_{HH}$ 5.0 Hz, H$^8$), 6.39 (2H, t, $^3J_{HH}$ 5.8 Hz, H$^b$), 6.22 (2H, d, $^3J_{HH}$ 5.8 Hz, H$^c$), 5.95 (1H, t, $^3J_{HH}$ 5.8 Hz, H$^a$), 4.18 (2H, d, $^3J_{HH}$ 6.0 Hz, H$^e$), 2.57 (2H, t, $^3J_{HH}$ 7.3 Hz, H$^{13}$), 1.94 (2H, t, $^3J_{HH}$ 7.3 Hz, H$^{18}$), 1.65 (2H, quin, $^3J_{HH}$ 7.3 Hz, H$^{14}$), 1.49 (2H, quin, $^3J_{HH}$ 7.3 Hz, H$^{17}$), 1.36-1.33 (2H, bm, H$^{15}$), 1.30-1.26 (2H, bm, H$^{16}$), 0.98 (9H, s, H$^i$); $\delta_C$ (DMSO-d$_6$) 178.2 (C$^g$), 173.2 (C$^{12}$), 169.5 (C$^{19}$), 156.6 (C$^2$), 155.3 (C$^9$), 146.2 (C$^{1'}$), 143.5 (C$^{10'}$), 138.5 (C$^7$), 135.4 (C$^4$), 133.8 (C$^5$), 130.2 (C$^{6'}$), 126.6 (C$^8$), 126.5 (C$^{4'}$), 125.9 (C$^3$), 119.0 (C$^6$), 103.1 (C$^d$), 87.7-87.6 (C$^b$ C$^{b'}$), 84.5 (C$^a$), 84.4-84.3 (C$^c$ C$^{c'}$), 40.6 (C$^e$), 38.4 (C$^h$), 36.4 (C$^{13}$), 32.7 (C$^{18}$), 28.9 (C$^{15}$), 28.8 (C$^{16}$), 27.6 (3C, C$^i$), 25.5 (C$^{17}$), 25.4 (C$^{14}$); *m/z* (HRMS$^+$) 688.1774 [M-Cl]$^+$ ($C_{32}H_{39}N_5O_4{}^{35}Cl^{96}Ru$ requires 688.1767).

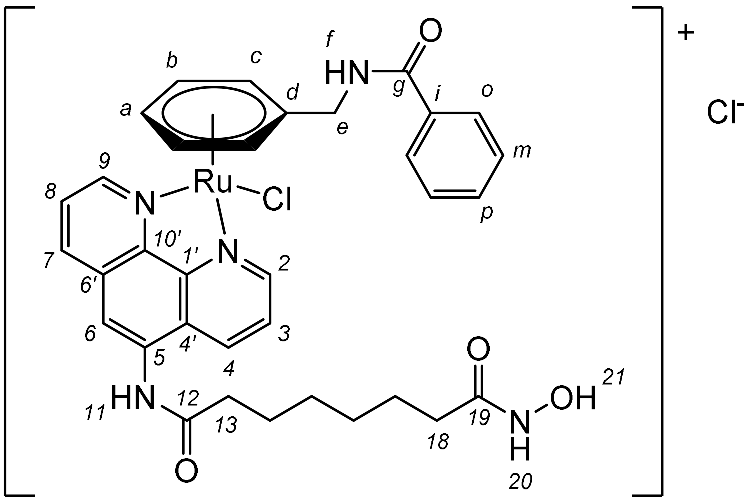


*Chloro(N-benzoyl-η^6^-benzylamide)(**L^1^**) ruthenium(II) chloride* **8c**

**7c** (0.040 g, 0.052 mmol) and ***L^1^*** (0.038 g, 0.104 mmol) were suspended in anhydrous MeOH (10 mL) and the solution was heated to 40 °C under an argon atmosphere, for 16 h. The volume of the solution was reduced to 1.5 mL and purified by reverse phase HPLC ($H_2O$ : MeOH) to yield the *title compound* (0.012 g, 31%) as a dark yellow solid; $\delta_H$ (DMSO-$d_6$) 10.6 (1H, s, $H^{11}$), 10.4 (1H, s, $H^{21}$), 10.0 (1H, d, $^3J_{HH}$ 5.0 Hz, $H^2$), 9.84 (1H, d, $^3J_{HH}$ 5.1 Hz, $H^9$), 9.15 (1H, t, $^3J_{HH}$ 5.7 Hz, $H^f$), 9.00 (1H, d, $^3J_{HH}$ 8.5 Hz, $H^4$), 8.75 (1H, d, $^3J_{HH}$ 8.3 Hz, $H^7$), 8.65 (1H, s, $H^{20}$), 8.43 (1H, s, $H^6$), 8.10 (1H, dd, $^3J_{HH}$ 8.5 Hz $^3J_{HH}$ 5.0 Hz, $H^3$), 8.00 (1H, dd, $^3J_{HH}$ 8.3 Hz $^3J_{HH}$ 5.1 Hz, $H^8$), 7.64 (2H, d, $^3J_{HH}$ 6.9 Hz, $H^o$), 7.40 (1H, t, $^3J_{HH}$ 6.9 Hz, $H^p$), 7.37 (2H, t, $^3J_{HH}$ 6.9 Hz, $H^m$), 6.43 (2H, d, $^3J_{HH}$ 5.8 Hz, $H^c$), 6.37 (2H, t, $^3J_{HH}$ 5.8 Hz, $H^b$), 6.05 (1H, t, $^3J_{HH}$ 5.8 Hz, $H^a$), 4.35 (2H, d, $^3J_{HH}$ 5.7 Hz, $H^e$), 2.57 (2H, t, $^3J_{HH}$ 7.1 Hz, $H^{13}$), 1.95 (2H, t, $^3J_{HH}$ 7.1 Hz, $H^{18}$), 1.68-1.64 (2H, bm, $H^{14}$), 1.51-1.47 (2H, bm, $H^{17}$), 1.38-1.34 (2H, bm, $H^{15}$), 1.31-1.27 (2H, bm, $H^{16}$); $\delta_C$ (DMSO-$d_6$) 173.2 ($C^{12}$), 169.5 ($C^{19}$), 166.5 ($C^g$), 156.5 ($C^2$), 155.2 ($C^9$), 146.1 ($C^{1'}$), 143.5 ($C^{10'}$), 138.4 ($C^7$), 135.2 ($C^4$), 133.8 ($C^{6'}$), 133.5 ($C^i$), 131.9 ($C^p$), 130.1 ($C^{4'}$), 128.6 (2C, $C^m$), 127.6 (2C, $C^o$), 126.6 ($C^8$), 126.5 ($C^5$), 125.8 ($C^3$), 118.9 ($C^6$), 86.6 ($C^a$), 86.2 (2C, $C^b$), 86.1 (2C, $C^c$), 86.0 ($C^d$), 40.9 ($C^e$), 36.4 ($C^{13}$), 32.7 ($C^{18}$), 28.9 ($C^{16}$), 28.8 ($C^{15}$), 25.5 ($C^{14}$), 25.4 ($C^{17}$); *m/z* ($HRMS^+$) 708.1465 $[M-Cl]^+$ ($C_{34}H_{35}N_5O_4{}^{35}Cl^{96}Ru$ requires 708.1454).

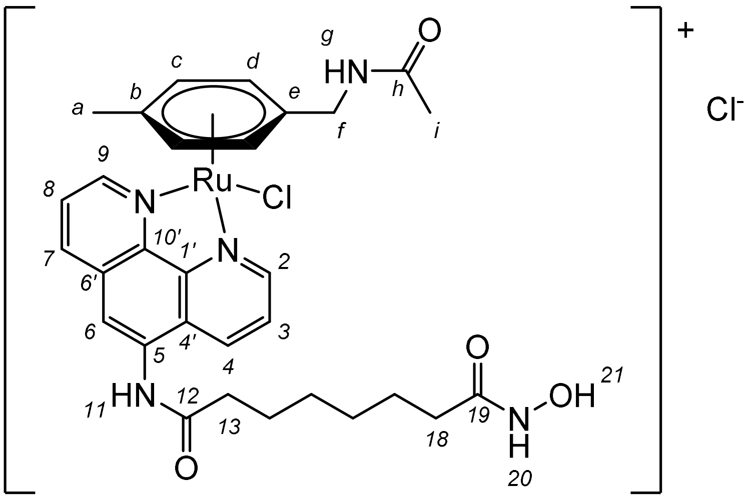

*Chloro(η$^6$-4-methyl-N-acetylbenzylamide)(**L$^1$**) ruthenium(II) chloride* **8d**

**7e** (0.038 g, 0.056 mmol) and ***L$^1$*** (0.041 g, 0.113 mmol) were dissolved in anhydrous MeOH (10 mL) and the reaction heated to 40 °C under an argon atmosphere for 16 h. The volume of the resulting solution was reduced to 1.5 mL and purified by reverse phase HPLC ($H_2O$ : MeOH) to afford the *title compound* (0.053 g, 85%) as a yellow solid; **$\delta_H$** (DMSO-d$_6$) 10.6 (1H, s, H$^{11}$), 10.4 (1H, s, H$^{21}$), 9.92 (1H, d, $^3J_{HH}$ 5.0 Hz, H$^2$), 9.79 (1H, d, $^3J_{HH}$ 5.0 Hz, H$^9$), 9.06 (1H, d, $^3J_{HH}$ 8.7 Hz, H$^4$), 8.81 (1H, d, $^3J_{HH}$ 8.2 Hz, H$^7$), 8.64 (1H, s, H$^{20}$), 8.47 (1H, s, H$^6$), 8.31 (1H, t, $^3J_{HH}$ 5.7 Hz, H$^g$), 8.15 (1H, dd, $^3J_{HH}$ 8.7 Hz $^3J_{HH}$ 5.0 Hz, H$^3$), 8.04 (1H, dd, $^3J_{HH}$ 8.2 Hz $^3J_{HH}$ 5.0 Hz, H$^8$), 6.36 (2H, m, H$^d$), 6.07 (1H, d, $^3J_{HH}$ 5.7 Hz, H$^c$), 6.06 (1H, d, $^3J_{HH}$ 5.7 Hz, H$^{c'}$), 3.43 (2H, d, $^3J_{HH}$ 5.7 Hz, H$^f$), 2.56 (2H, t, $^3J_{HH}$ 7.4 Hz, H$^{13}$), 2.13 (3H, s, H$^a$), 1.93 (2H, t, $^3J_{HH}$ 7.4 Hz, H$^{18}$), 1.65 (2H, quin, $^3J_{HH}$ 7.4 Hz, H$^{14}$), 1.49 (2H, quin, $^3J_{HH}$ 7.4 Hz, H$^{17}$), 1.46 (3H, s, H$^i$), 1.37-1.32 (2H, m, H$^{15}$), 1.30-1.26 (2H, m, H$^{17}$); **$\delta_C$** (DMSO-d$_6$) 173.2 (C$^{12}$), 169.6 (C$^h$), 169.5 (C$^{19}$), 156.3 (C$^2$), 155.1 (C$^9$), 146.2 (C$^{1'}$), 143.6 (C$^{10'}$), 138.4 (C$^7$), 135.2 (C$^4$), 133.8 (C$^5$), 130.1 (C$^{6'}$), 127.6 (C$^{4'}$), 126.6 (C$^8$), 125.8 (C$^3$), 119.0 (C$^6$), 104.8 (C$^b$), 95.4 (C$^e$), 88.5-88.4 (C$^d$, C$^{d'}$), 82.9-82.7 (C$^c$, C$^{c'}$), 39.3 (C$^f$), 36.4 (C$^{13}$), 32.7 (C$^{18}$), 28.9 (C$^{16}$), 28.8 (C$^{15}$), 25.5 (C$^{17}$), 25.4 (C$^{14}$), 22.4 (C$^i$), 18.8 (C$^a$); *m/z* (HRMS$^+$) 660.1476 [M-Cl]$^+$ ($C_{30}H_{35}N_5O_4{}^{35}Cl^{96}Ru$ requires 660.1454).

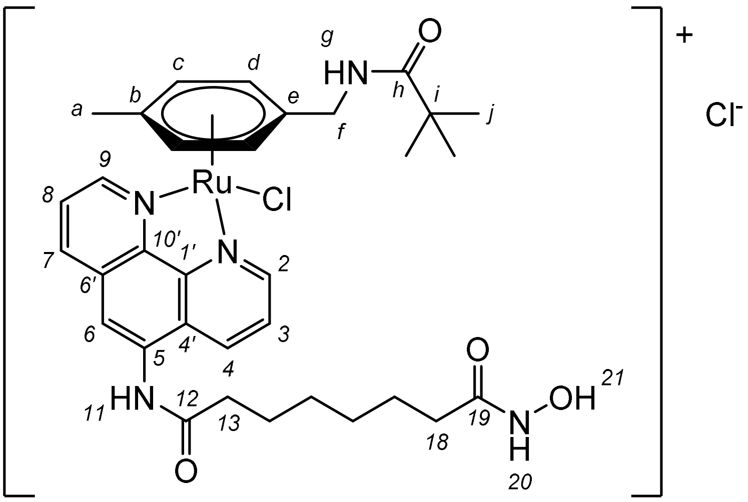


*Chloro($\eta^6$-4-methyl-N-trimethylacetylbenzylamide)($L^1$) ruthenium(II) chloride* **8e**

**7f** (0.037 g, 0.049 mmol) and $\boldsymbol{L^1}$ (0.035 g, 0.098 mmol) were dissolved in anhydrous MeOH (10 mL) and the reaction heated to 40 °C under an argon atmosphere for 16 h. The volume of the resulting solution was reduced to 1.5 mL and purified by reverse phase HPLC ($H_2O$ : MeOH) to afford the *title compound* (0.027 g, 75%) as a yellow solid; $\boldsymbol{\delta_H}$ (DMSO-$d_6$) 10.56 (1H, s, $H^{11}$), 10.36 (1H, s, $H^{21}$), 9.94 (1H, d, $^3J_{HH}$ 5.3 Hz, $H^2$), 9.80 (1H, d, $^3J_{HH}$ 5.5 Hz, $H^9$), 9.05 (1H, d, $^3J_{HH}$ 8.2 Hz, $H^4$), 8.82 (1H, d, $^3J_{HH}$ 8.1 Hz, $H^7$), 8.64 (1H, s, $H^{20}$), 8.48 (1H, s, $H^6$), 8.16 (1H, dd, $^3J_{HH}$ 8.2 Hz $^3J_{HH}$ 5.3 Hz, $H^3$), 8.05 (1H, dd, $^3J_{HH}$ 8.1 Hz $^3J_{HH}$ 5.5 Hz, $H^8$), 7.94 (1H, t, $^3J_{HH}$ 5.8 Hz, $H^g$), 6.29 (1H, d, $^3J_{HH}$ 6.0 Hz, $H^d$), 6.28 (1H, d, $^3J_{HH}$ 6.0 Hz, $H^{d'}$), 6.07 (2H, bm, $H^c H^{c'}$), 3.94 (2H, d, $^3J_{HH}$ 5.8 Hz, $H^f$), 2.56 (2H, t, $^3J_{HH}$ 7.2 Hz, $H^{13}$), 2.15 (3H, s, $H^a$), 1.94 (2H, t, $^3J_{HH}$ 7.2 Hz, $H^{18}$), 1.66 (2H, 2H, quin, $^3J_{HH}$ 7.2 Hz, $H^{14}$), 1.49 (2H, quin, $^3J_{HH}$ 7.2 Hz, $H^{17}$), 1.38-1.33 (2H, m, $H^{15}$), 1.31-1.27 (2H, m, $H^{16}$), 0.80 (9H, s, $H^j$); $\boldsymbol{\delta_C}$ (DMSO-$d_6$) 177.8 ($C^h$), 173.1 ($C^{12}$), 169.5 ($C^{19}$), 156.2 ($C^2$), 154.9 ($C^9$), 146.4 ($C^{1'}$), 143.7 ($C^{10'}$), 138.4 ($C^7$), 135.2 ($C^4$), 133.7 ($C^{4'}$), 130.2 ($C^{6'}$), 126.6 ($C^8$), 126.5 ($C^5$), 125.8 ($C^3$), 118.9 ($C^6$), 104.4 ($C^b$), 95.8 ($C^e$), 87.8-87.7 ($C^d$, $C^{d'}$), 83.3-83.1 ($C^c$, $C^{c'}$), 39.5 ($C^f$), 38.3 ($C^i$), 36.4 ($C^{13}$), 32.7 ($C^{18}$), 28.8 ($C^{15}$, $C^{16}$), 27.5 ($C^j$), 25.4 ($C^{14}$, $C^{17}$), 18.9 ($C^a$); *m/z* (HRMS$^+$) 702.1948 $[M\text{-}Cl]^+$ ($C_{33}H_{41}N_5O_4{}^{35}Cl^{96}Ru$ requires 702.1923).

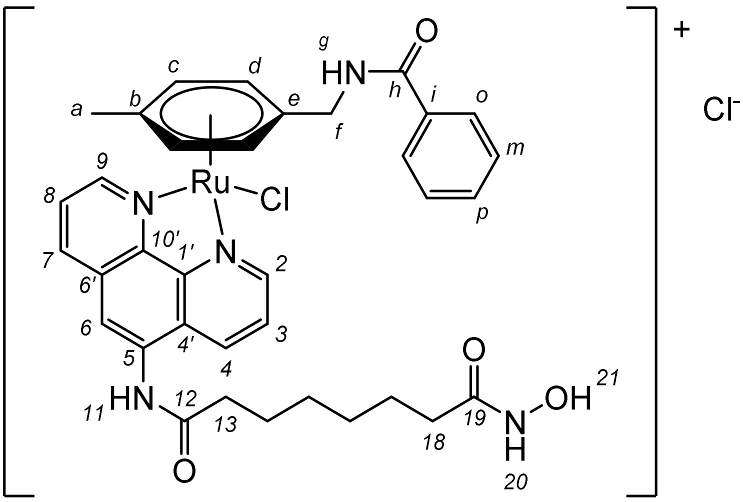

*Chloro(4-methyl-N-benzoyl-$\eta^6$-benzylamide)($\boldsymbol{L^1}$) ruthenium(II) chloride* **8f**

**7g** (0.040 g, 0.050 mmol) and $\boldsymbol{L^1}$ (0.036 g, 0.100 mmol) were dissolved in anhydrous MeOH (10 mL) and the reaction heated to 40 °C under an argon atmosphere for 16 h. The volume of the resulting solution was reduced to 1.5 mL and purified by reverse phase HPLC ($H_2O$ : MeOH) to afford the *title compound* (0.050 g, 67%) as a yellow solid; $\boldsymbol{\delta_H}$ (DMSO-$d_6$) 10.53 (1H, s, $H^{11}$), 10.38 (1H, s, $H^{21}$), 9.92 (1H, d, $^3J_{HH}$ 5.0 Hz, $H^2$), 9.78 (1H, d, $^3J_{HH}$ 4.8 Hz, $H^9$), 8.96-8.94 (2H, m, $H^{4'}$, $H^g$), 8.68 (1H, d, $^3J_{HH}$ 8.0 Hz, $H^7$), 8.65 (1H, s, $H^{20}$), 8.40 (1H, s, $H^6$), 8.04 (1H, dd, $^3J_{HH}$ 8.1 Hz $^3J_{HH}$ 5.0 Hz, $H^3$), 7.93 (1H, dd, $^3J_{HH}$ 8.0 Hz $^3J_{HH}$ 4.8 Hz, $H^8$), 7.44 (1H, t, $^3J_{HH}$ 7.0 Hz, $H^p$), 7.36 (2H, d, $^3J_{HH}$ 7.0 Hz, $H^o$), 7.29 (2H, t, $^3J_{HH}$ 7.0 Hz, $H^m$), 6.55 (2H, bm, $H^d H^{d'}$), 6.05 (1H, d, $^3J_{HH}$ 7.0 Hz, $H^c$), 6.03 (1H, d, $^3J_{HH}$ 7.0 Hz, $H^{c'}$), 4.12 (2H, d, $^3J_{HH}$ 6.0 Hz, $H^f$), 2.59 (2H, t, $^3J_{HH}$ 7.0 Hz, $H^{13}$), 2.18 (3H, s, $H^a$), 1.96 (2H, t, $^3J_{HH}$ 7.0 Hz, $H^{18}$), 1.68 (2H, quin, $^3J_{HH}$ 7.0 Hz, $H^{14}$), 1.52 (2H, quin, $^3J_{HH}$ 7.0 Hz, $H^{17}$), 1.39-1.36 (2H, m, $H^{15}$), 1.34-1.31 (2H, m, $H^{16}$); $\boldsymbol{\delta_C}$ (DMSO-$d_6$) 173.1 ($C^{12}$), 169.5 ($C^{19}$), 165.7 ($C^h$), 156.1 ($C^2$), 154.9 ($C^9$), 146.3 ($C^{1'}$), 143.6 ($C^{10'}$), 138.2 ($C^7$), 134.9 ($C^4$), 133.6 ($C^{4'}$), 132.9 ($C^i$), 131.8 ($C^p$), 130.2 ($C^{6'}$), 128.4 (2C, $C^m$), 127.7 ($C^5$), 127.4 (2C, $C^o$), 126.4 ($C^8$), 125.6 ($C^3$), 118.7 ($C^6$), 107.4 ($C^b$), 94.6 ($C^e$), 89.8-89.7 ($C^d$, $C^{d'}$), 81.5-81.2 ($C^c$, $C^{c'}$), 39.6 ($C^f$), 36.4 ($C^{13}$), 32.7 ($C^{18}$), 28.8 ($C^{15}$, $C^{16}$), 25.5 ($C^{14}$, $C^{17}$), 19.3 ($C^a$); *m/z* (HRMS$^+$) 722.1605 $[M-Cl]^+$ ($C_{35}H_{37}N_5O_4{}^{35}Cl^{96}Ru$ requires 722.1610).

## 3. Aqueous Stability Assay

To assess stability with respect to hydrolysis of the Ru-Cl bond, $^1$H-NMR ($D_2O$, 298 K, 400 MHz) studies were carried out, monitoring the resonances of the capping *p*-cymene group. To distinguish the Ru-Cl complex (**1**) and the Ru-$OD_2$ complex (**1a**), NMR spectra were run in 100 mM NaCl solution (Fig S2 iii) and $AgNO_3$ (Fig S2 iv), respectively. Complex **1** was dissolved in $D_2O$ and hydrolysis of the Ru-Cl bond was monitored over the course of 96 h. As shown in Fig S2, after 1 h, the complex

remains intact as the Ru-Cl complex. After 96 h, approximately 90% of the complex remains as the Ru-Cl complex, with 10% hydrolysis occurring.

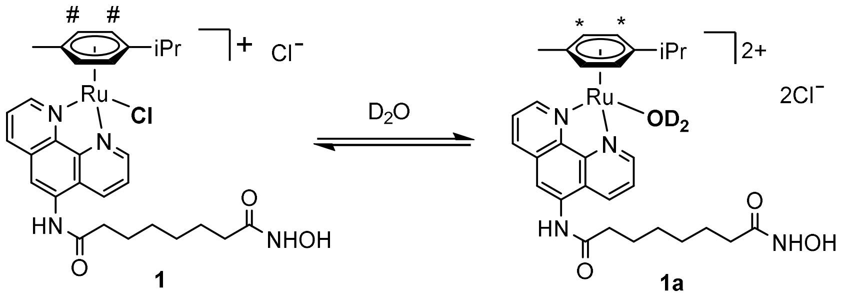


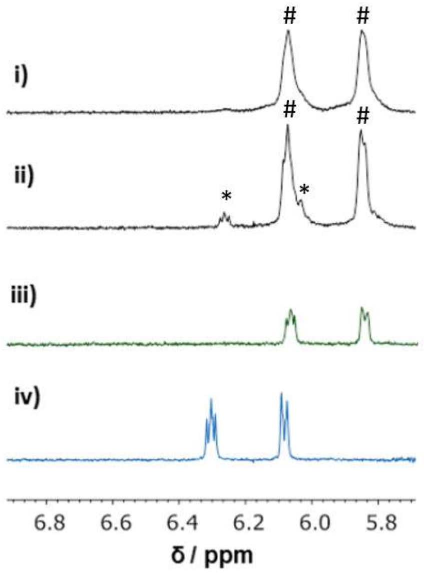


Figure S2. $^{1}$H-NMR spectra ($D_2O$, 298 K, 400 MHz) of the *p*-cymene proton region of complex **1**, under the following conditions: i) $D_2O$, 1 h, showing majority intact Ru-Cl bond; ii) $D_2O$, 96 h, showing majority Ru-Cl bond intact with around 10% Ru-$OD_2$ complex; iii) 100 mM NaCl, showing Ru-Cl complex only; iv) $AgNO_3$, 24 h, showing Ru-$OD_2$ complex only. Symbol # shows peaks for Ru-Cl complex, symbol * shows peaks for Ru-$OD_2$ complex.

## 4. HPLC Purification and Analysis

Reverse phase HPLC analysis was performed at 298 K on a Interchim PuriFlash 4250 system, Waters XBridge Prep-C18 –19x50 mm (5 µm) column was used for complexes **1** and **8a-f**, times varying from 2 –5 min. A solvent system of $H_2O$ / MeOH (gradient elution) was used. The UV/Vis and fluorescence detectors were set at appropriate wavelengths according to the species being analysed. Channel 1: UV600 SCAN – 225-600. Channel 2: UV600: SIG1 – 254 nm.

# 5. Enzyme Inhibition Assay

HDAC enzyme inhibition assays were carried out for as stated using commercially available assay kits (*BPS Bioscience*) and undertaken in a 96 well plate. All measurements were carried out in triplicate and standard deviation errors are included in the plots. Human recombinant enzymes HDAC1 FLAG-tag His-tag and HDAC6 GST-tag were used. Potential inhibitors were dosed at the required concentration (either 1 µM/0.1µM for the two concentration point assays or 10 to 0.004 µM for $IC_{50}$ measurements) from original DMSO stock (10 mM) and diluted using assay buffer (25 mM Tris/Cl, pH 8.0, 137 mM NaCl, 2.7 mM KCl, 1 mM $MgCl_2$). A master mixture was made using assay buffer, acetylated substrate specific to HDAC enzyme (200 µM) and BSA (1 mg/mL) in a ratio of buffer:substrate:BSA (6:1:1) and 40 µL added to each well plate. Buffer (10 µL) was added to all blank wells and inhibitor (5 µL) was added to all potential inhibitor wells. HDAC enzymes were diluted based on their stated activity and 5 µL was added to all wells except blanks and the plate incubated at 37 °C for 30 min. Developer was added and the mixture incubated at room temperature for a further 15 min. Fluorescence was measured using a Synergy H4 microplate reader (λex = 360 nm, λem = 460 nm). Percentage HDAC activity is determined by quantification of fluorescence relative to control, with no added inhibitor. Data from the two point assays with errors from standard deviation are shown in Table S1 (and shown graphically in Figure 2). $IC_{50}$ values are calculated using least squares regression fitting to equation S2, where $Y$ = calculated HDAC activity at given concentration, $X$ = concentration of inhibitor, $a$ = gradient scaling factor and $SP$ = saturation point (i.e. 100% activity). Errors calculated using the formula for standard error.

$$Y = \frac{\exp(a \times (X - I\ _{50}))}{1 + \exp(a \times (X - IC_{50}))} \times SP \qquad \text{(S2)}$$

**Table S1**. two concentration point HDAC1 and HDAC6 inhibition assays of Ru complexes **1** and **8a-f**, and control compounds, measured using commercially available assay kits. All assays carried out in triplicate.

| | HDAC1 | | HDAC6 | |
|---|---|---|---|---|
| | 1 uM | 0.1 uM | 1 uM | 0.1 uM |
| **SAHA** | 12 ± 7 | 70 ± 15 | 4 ± 2 | 22 ± 1 |
| **L¹** | 6 ± 13 | 44 ± 7 | 1.5 ± 1 | 17 ± 3 |
| **1** | 19 ± 25 | 90 ± 50 | 9 ± 3 | 30 ± 7 |
| **8a** | 12 ± 7 | 52 ± 5 | 19 ± 1 | 85 ± 20 |
| **8b** | 9 ± 8 | 31 ± 1 | 12 ± 5 | 80 ± 20 |
| **8c** | 17 ± 10 | 50 ± 3 | 23 ± 6 | 85 ± 5 |
| **8d** | 4.0 ± 0.1 | 51 ± 5 | 8 ± 2 | 49 ± 9 |
| **8e** | 8 ± 7 | 43 ± 7 | 7 ± 2 | 44 ± 4 |
| **8f** | 2.5 ± 2 | 31 ± 9 | 2.6 ± 2 | 28 ± 3 |

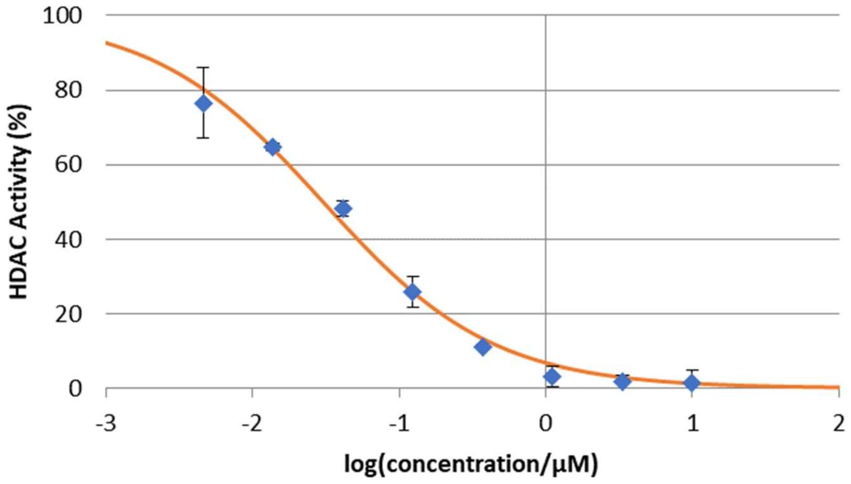


Fig. S3. $IC_{50}$ determination for SAHA against HDAC1. $IC_{50}$ = 30 ± 4 nM.

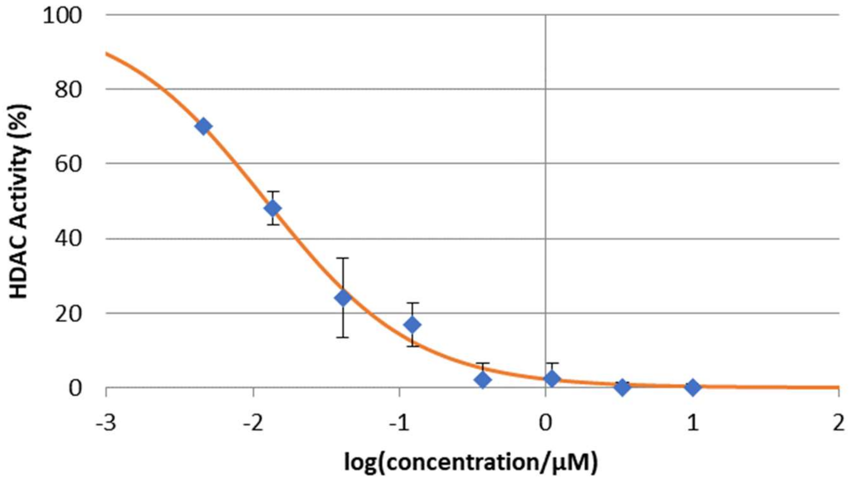


Fig S4. $IC_{50}$ determination for SAHA against HDAC6. $IC_{50}$ = 12 ± 1 nM.

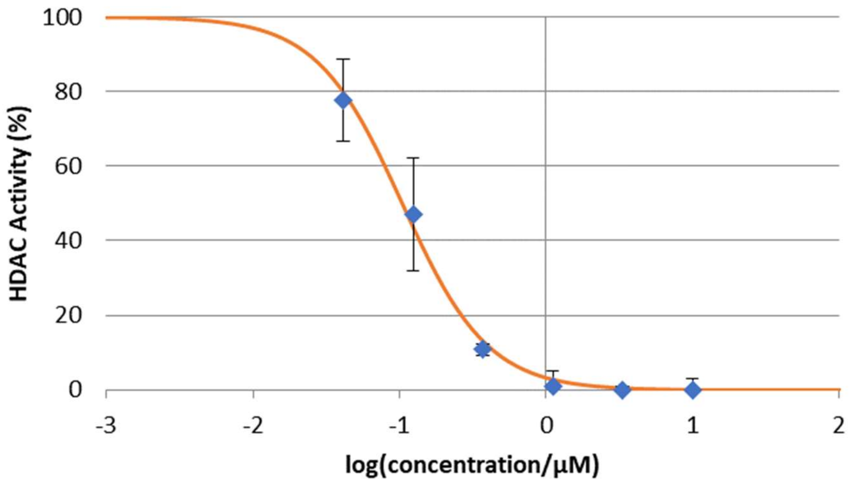


Fig S5. $IC_{50}$ determination for ligand $\mathbf{L^1}$ against HDAC1. $IC_{50}$ = 105 ± 15 nM.

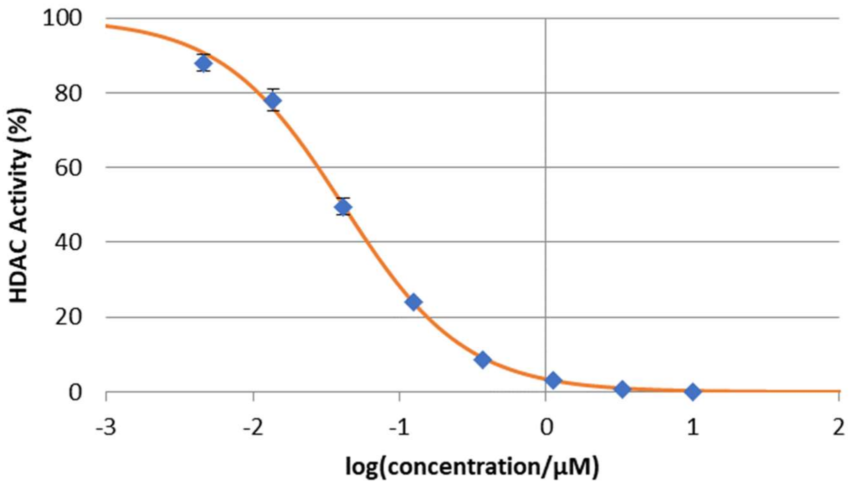


Fig S6. $IC_{50}$ determination for ligand $\mathbf{L^1}$ against HDAC6. $IC_{50}$ = 41 ± 2 nM.

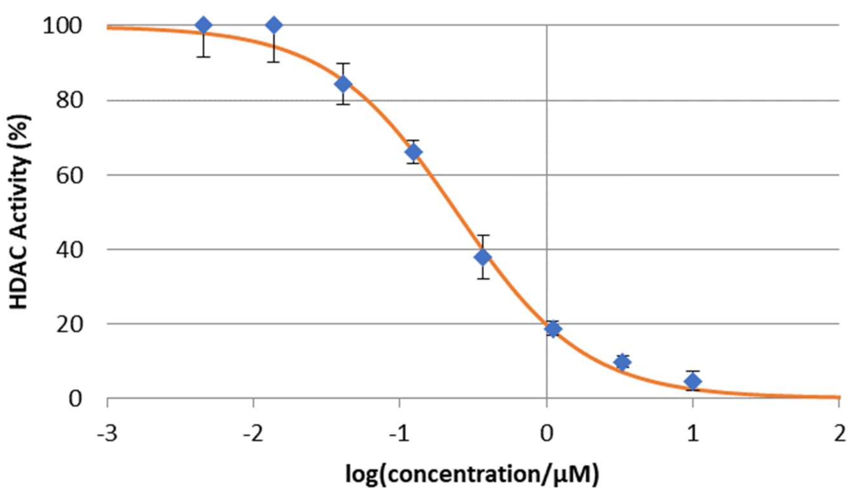


Fig S7. $IC_{50}$ determination for complex **1** against HDAC1. $IC_{50}$ = 240 ± 30 nM.

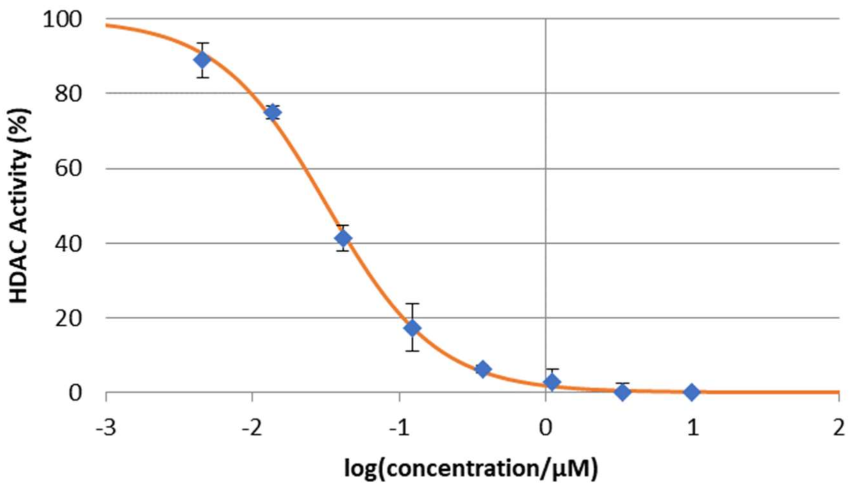


Fig S8. $IC_{50}$ determination for complex **1** against HDAC6. $IC_{50}$ = 32 ± 1 nM.

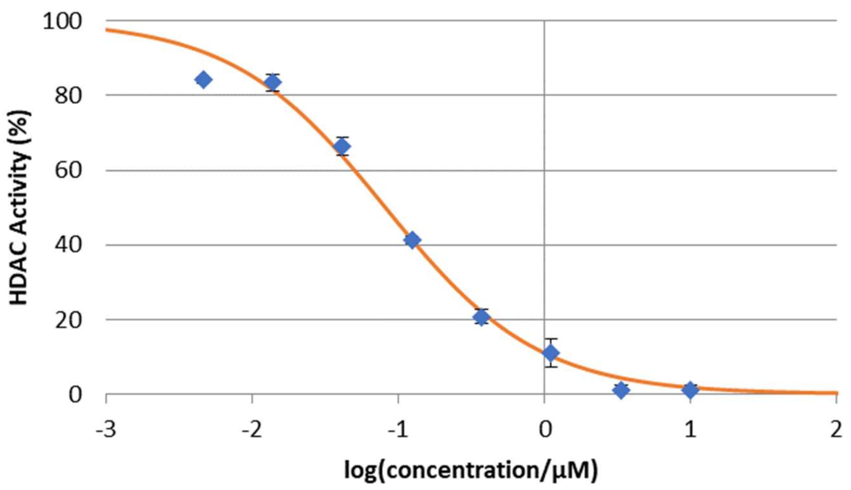


Fig S9. $IC_{50}$ determination for complex **8f** against HDAC1. $IC_{50}$ = 80 ± 10 nM.

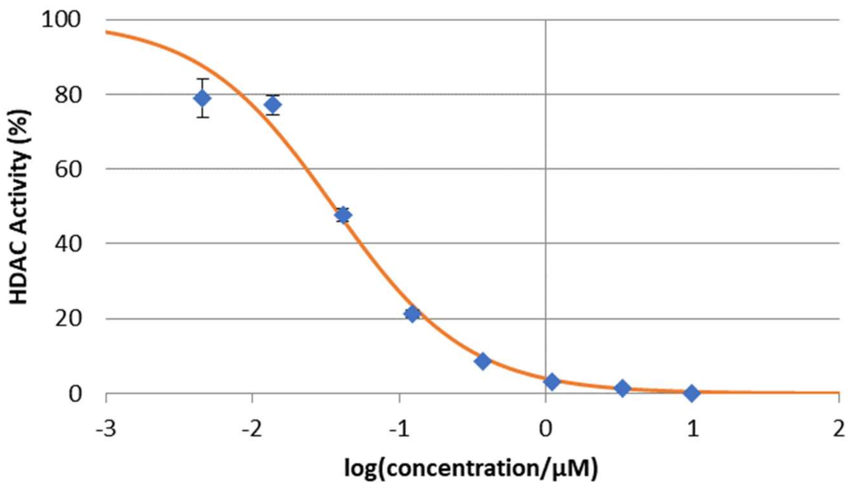


Fig S10. $IC_{50}$ determination for complex **8f** against HDAC6. $IC_{50}$ = 35 ± 6 nM.

# 6. Cell Viability Assays

Cellular behaviour of complexes **1**, **8a**-**8f**, SAHA and **L**[1] were conducted using MCF7 human breast adenocarcinoma cells using fluorescence and laser scanning confocal microscopy. Cells were maintained in exponential growth as monolayers in F-12/DMEM (Dulbecco's Modified Eagle Medium)

1:1 that was supplemented with 10% foetal bovine serum (FBS). Cells were grown in 75 $cm^2$ plastic culture flasks, with no prior surface treatment. Cultures were incubated at 37 °C, 20% average humidity and 5% (v/v) $CO_2$. Cells were harvested by treatment with 0.25% (v/v) trypsin solution for 5 min at 37 ºC. Cell suspensions were pelleted by centrifugation at 1000 rpm for 3 min and were re-suspended by repeated aspiration with a sterile plastic pipette. Microscopy cells were seeded in 12-well plates on 13mm 0.17mm thick standard glass cover-slips or un-treated iBibi 100 uL live cell channels and allowed to grow to 40% – 60% confluence, at 37 ºC in 5% $CO_2$. At this stage, the medium was replaced and cells were treated with compounds in varying concentrations (0.80 - 100 µM dissolved in cell culture media) and co-stains as appropriate.

After 96 h incubation, cell toxicity measurements were conducted using a ChemoMetec A/S NucleoCounter3000-Flexicyte instrument with Via1-cassette cell viability cartridge. The cellular stain Acridine Orange was used for cell detection and the nucleic acid stain DAPI was used to detect non-viable cells. The experiments were carried out in triplicate. Cell viability was corrected using a proliferation factor (*Equation S1*) where: $vib^I$ = corrected viability; $n_x$ = number of cells after incubation; $n_c$ = number of cells on loading; $vib_x$ = measured viability.

$$vib^I = \left(\frac{n_x}{n_c}\right) vib_x \tag{S1}$$

To investigate cell uptake mechanism, MCF7 cells were plated using the same method described in *Cell Viability Assay* and exposed to complexes **1** at the measured $IC_{50}$ concentrations (1.53 µM). Cells were allowed to incubate at room temperature for 1 h before being cooled to 4 °C for 24 h. After this time, cell number viability was 98% compared to the initial loading, suggesting no accumulation of complexes in cells at this temperature and, therefore, an uptake mechanism driven by active transport.

## 7. Cell Uptake Assay

In cellular uptake studies, cells were seeded in 6-well plates and allowed to grow to 80% – 100% confluence, at 37 ºC in 5% $CO_2$. At this stage, the media was replaced with media containing complexes **1** and **8a-8f** at their $EC_{50}$ concentration and total cellular ruthenium was determined using inductively coupled plasma mass spectrometry (ICP-MS).

Cells used for ICP-MS studies were prepared as follows.[1] Cells were cultured in a 6- well plate to 90% confluence. Cells were then counted ($10^7$ cells based on a cell volume of 4000 $\mu m^3$) and incubated with medium containing the complex and promoter/inhibitor for 96 h before being washed three times with phosphate-buffered saline (PBS). The cells were then treated with trypsin and harvested and diluted to 1 mL with PBS. Concentrated nitric acid (0.6 mL) was added and the samples were digested for 24 h at 37 °C. These digested samples were submitted for ICP-MS measurements. The samples were run against a series of Ru standards, and the measured concentration was back calculated to find the total Ru concentration present in the original counted cells. In Fig. S11 cell uptake is compared to the inverse of the cell viability $EC_{50}$ value for all Ru complexes, showing the clear trend between the two values.

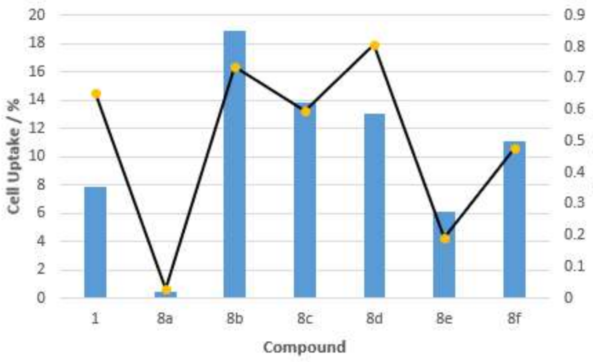


Fig. S11 Cell uptake (*blue bars*) and inverse of cell viability $EC_{50}$ (*yellow circles with black line*).

## 8. Computational Docking Studies

DFT optimisation of complex **1** was carried out using B3LYP hybrid functionals. For atoms H, C, N and O, the 6-31G* basis set was used, for Ru, the SDD basis set was required. Optimization calculations were carried out in the gas phase. The optimised structure was validated to be the lowest energy structure through vibrational frequencies calculations. The NMR spectra were also calculated and compared to the experimental spectra. All calculations were carried out with Gaussian09. The remaining structures (**L$^1$**, complexes **8c** and **8f**) were adapted from this optimized compound using Avogadro and bond length data.[2] The 3D model of SAHA was obtained from the ZINC compound database.[3]

Genetic Optimisation of Ligand Docking[4] (GOLD) was used to perform molecular docking of SAHA, L$^1$ and complexes **1**, **8c** and **8f** to the human HDAC1 and HDAC6 proteins (PDB: 4BKX, 5EDU). The Hermes Visualiser was used to prepare the ligands and proteins before docking was undertaken, the ruthenium ion was treated as a dummy atom by GOLD and all water/ligand molecules removed. The region of interest was specified as within 10 Å of the zinc ion present in the binding site. The complex was subjected to 10 genetic algorithm runs, looking for diverse solutions using the ChemScore fitness function. These were then analysed using the ChemScore components and modes of predicted binding. The docking poses were evaluated, and figures produced, using UCSF Chimera.[5]

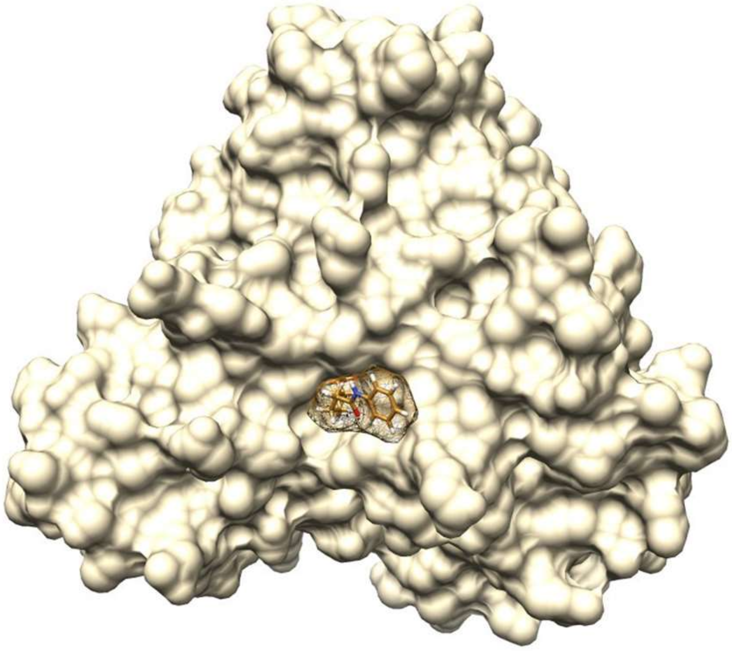

Fig S12. Computational docking study showing surface view of whole HDAC1 enzyme with SAHA bound in the active site.

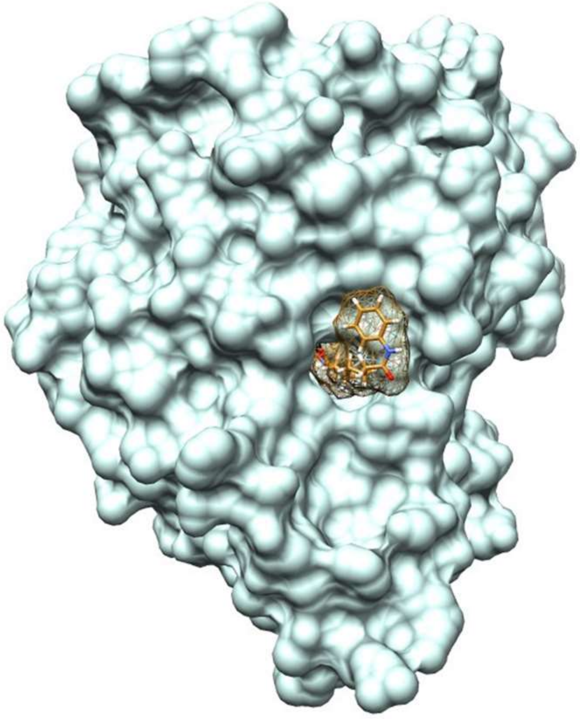

Fig S13. Computational docking study showing surface view of catalytic domain 2 of the HDAC6 enzyme with SAHA bound in the active site.

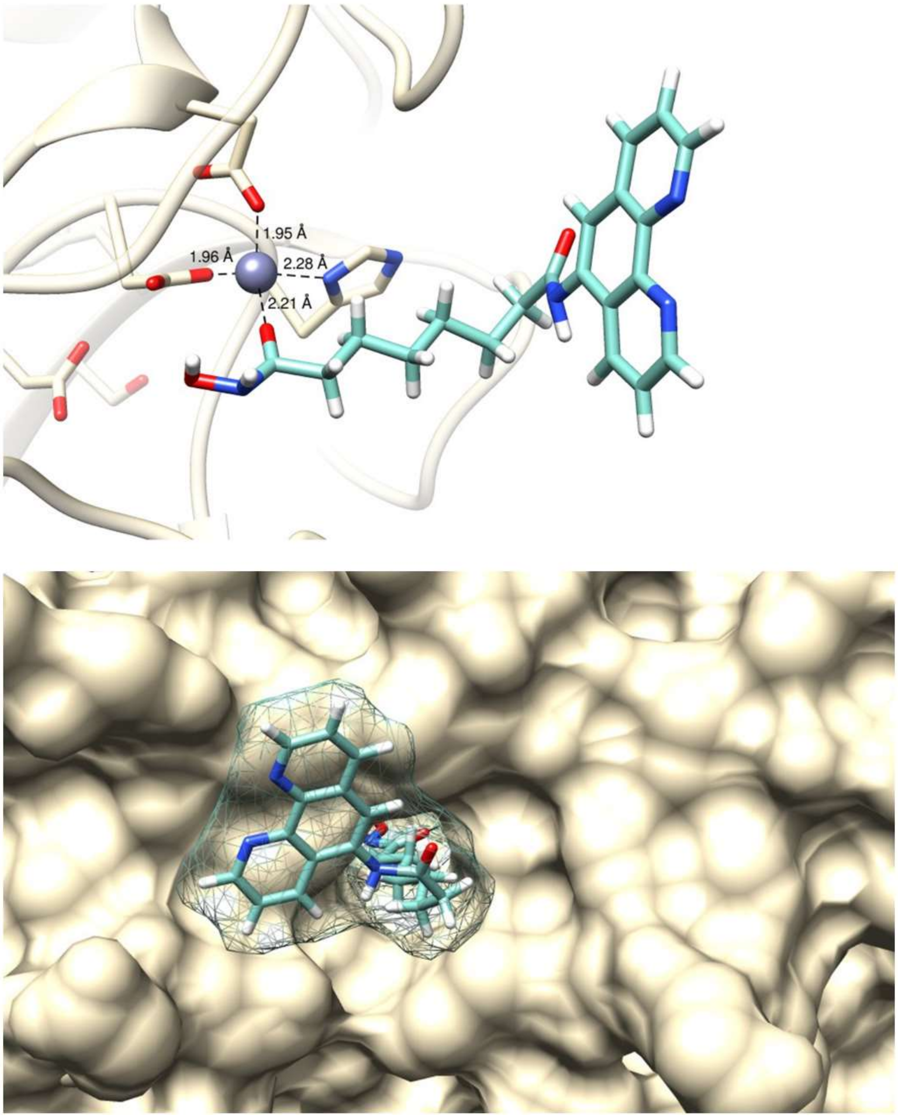


Fig S14. Computational docking study showing *upper*: Zn(II) coordination view of ligand $\mathbf{L^1}$ with HDAC1; *lower*: active site cavity entrance surface view of of ligand $\mathbf{L^1}$ with HDAC1.

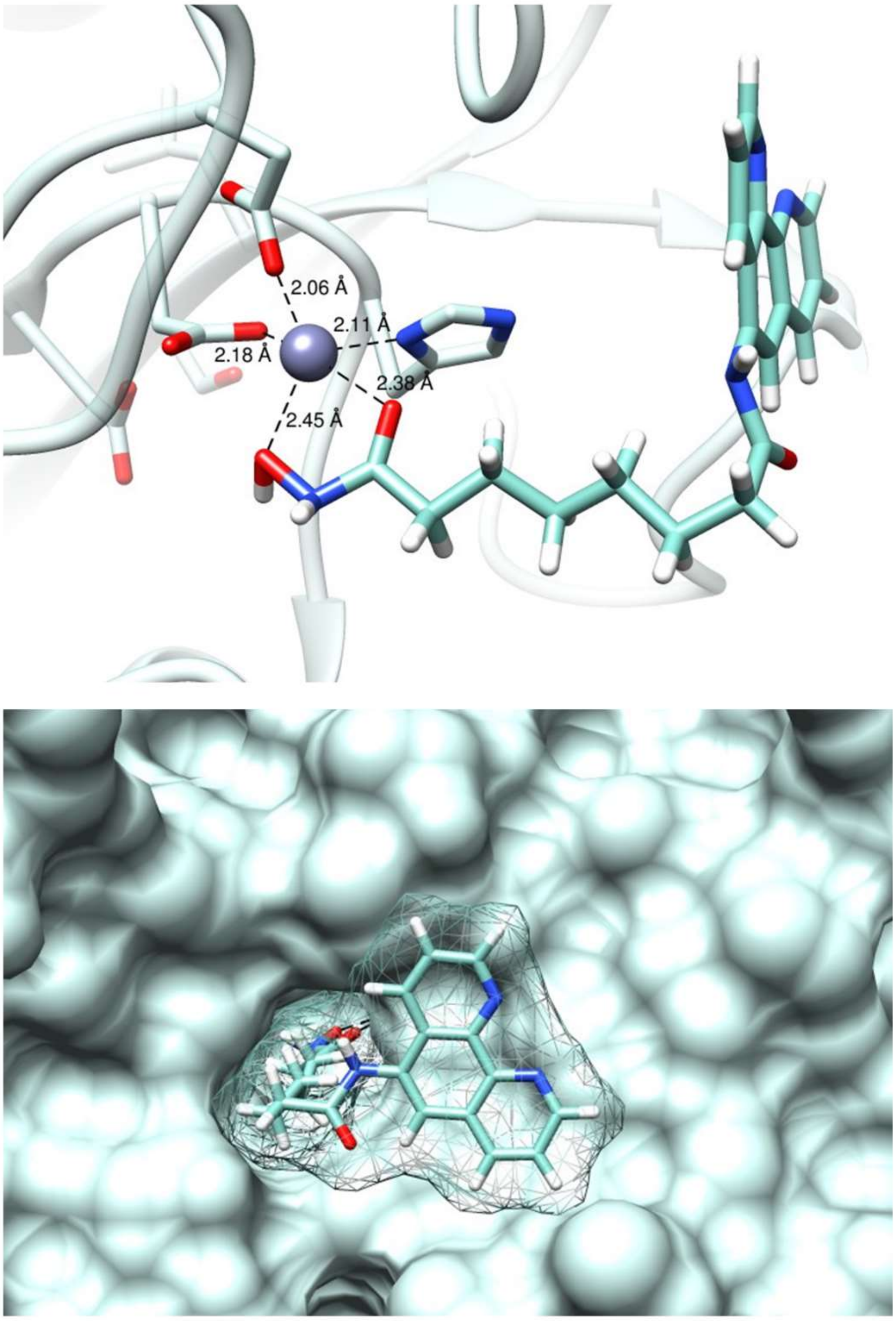


Fig S15. Computational docking study showing *upper*: Zn(II) coordination view of ligand **L$^1$** with HDAC6; *lower*: active site cavity entrance surface view of of ligand **L$^1$** with HDAC6.

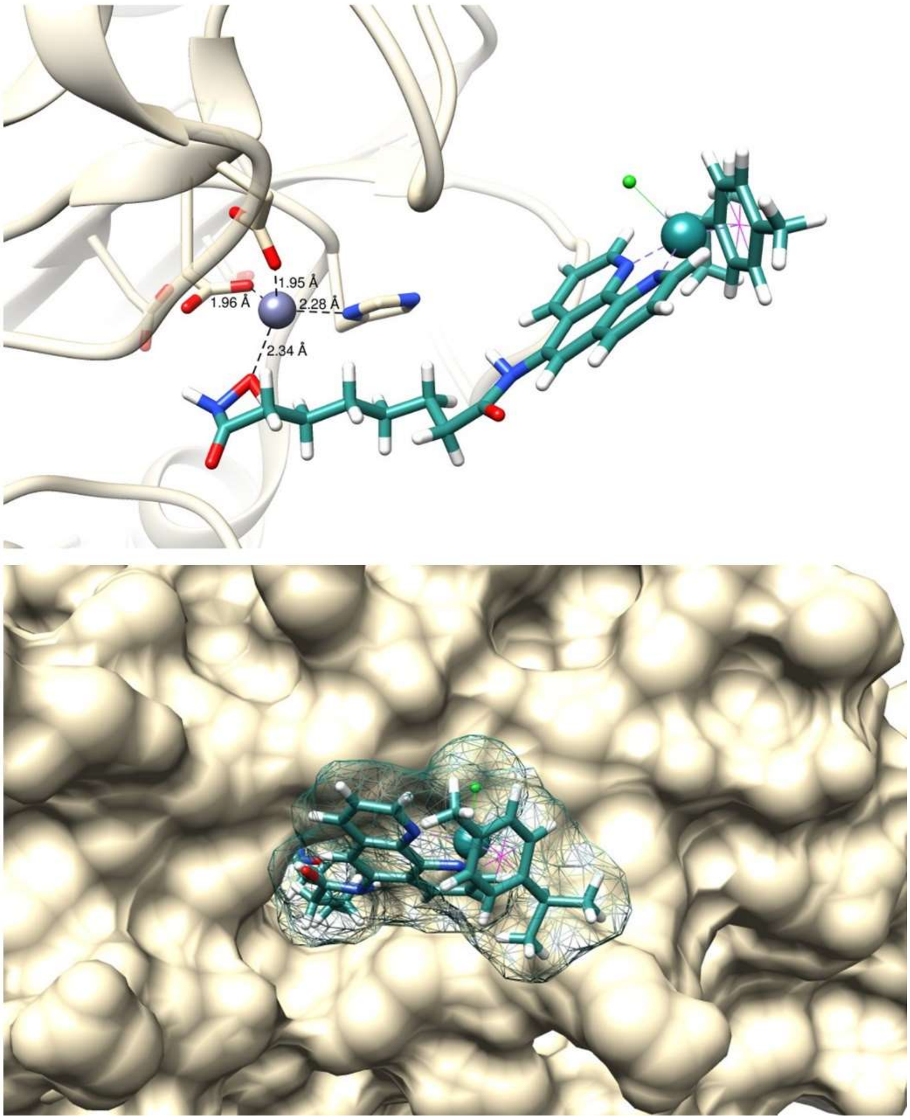


Fig S16. Computational docking study showing *upper*: Zn(II) coordination view of complex **1** with HDAC1; *lower*: active site cavity entrance surface view of complex **1** with HDAC1.

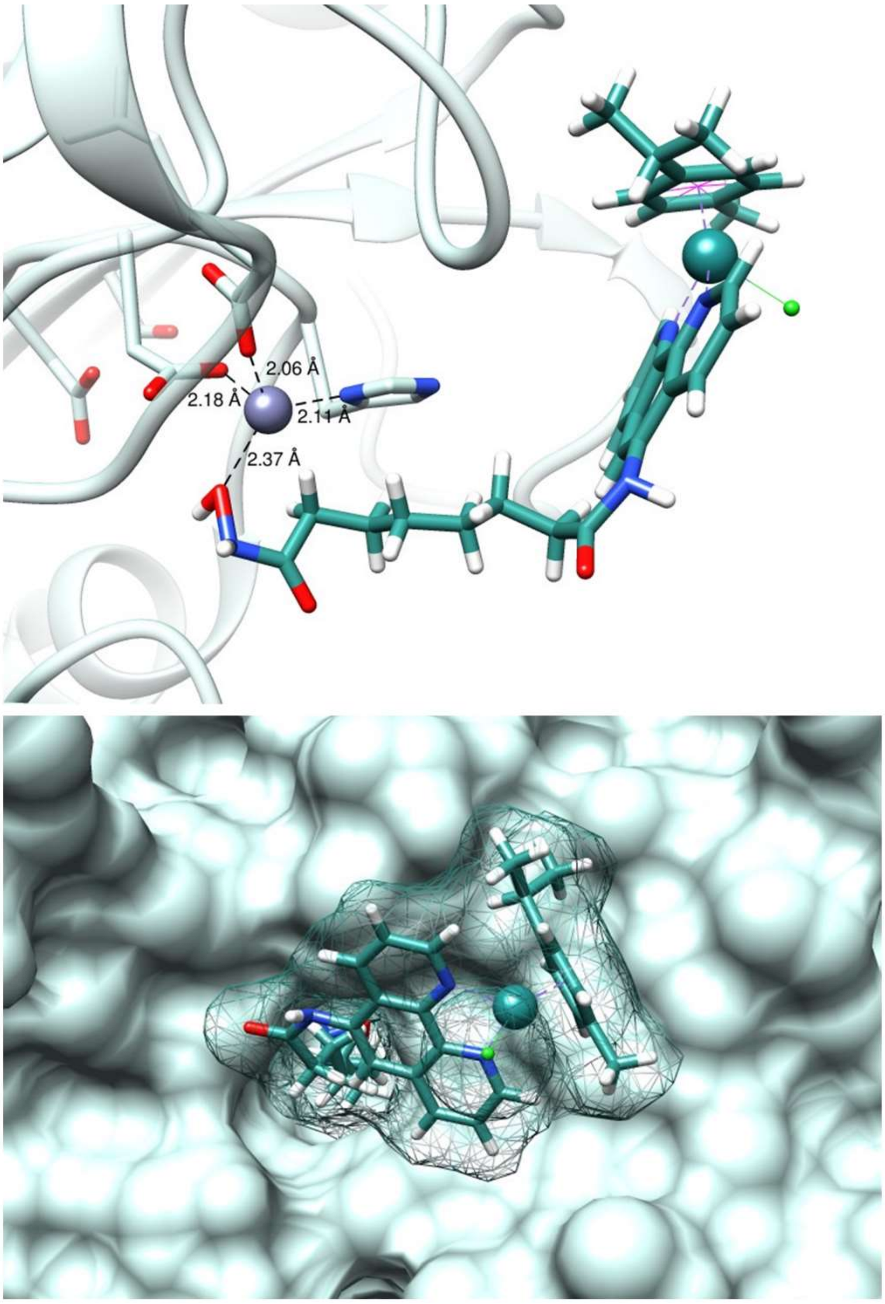


Fig S17. Computational docking study showing *upper*: Zn(II) coordination view of complex **1** with HDAC6; *lower*: active site cavity entrance surface view of complex **1** with HDAC6.

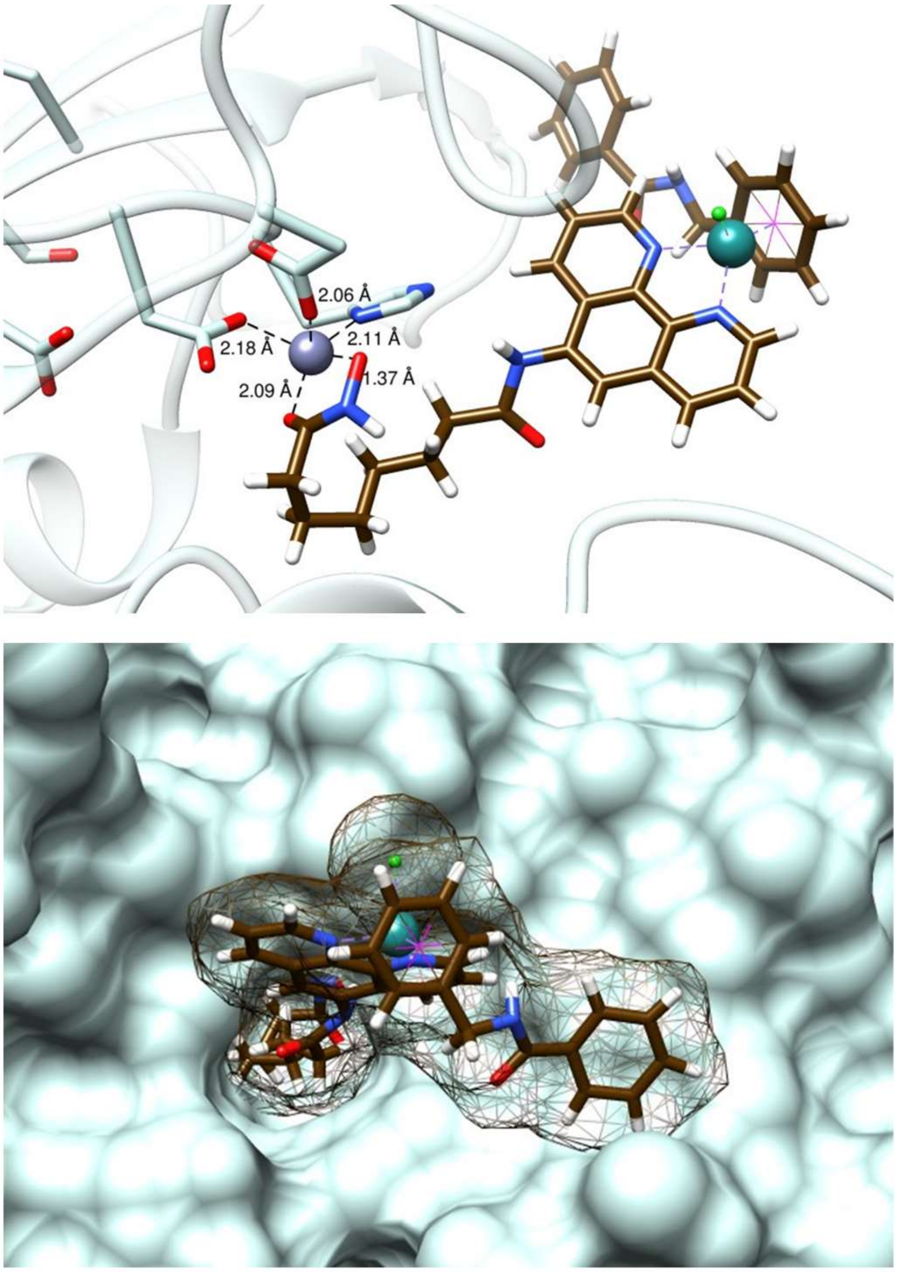


Fig S18. Computational docking study showing *upper*: Zn(II) coordination view of complex **8c** with HDAC6; *lower*: active site cavity entrance surface view of complex **8c** with HDAC6.

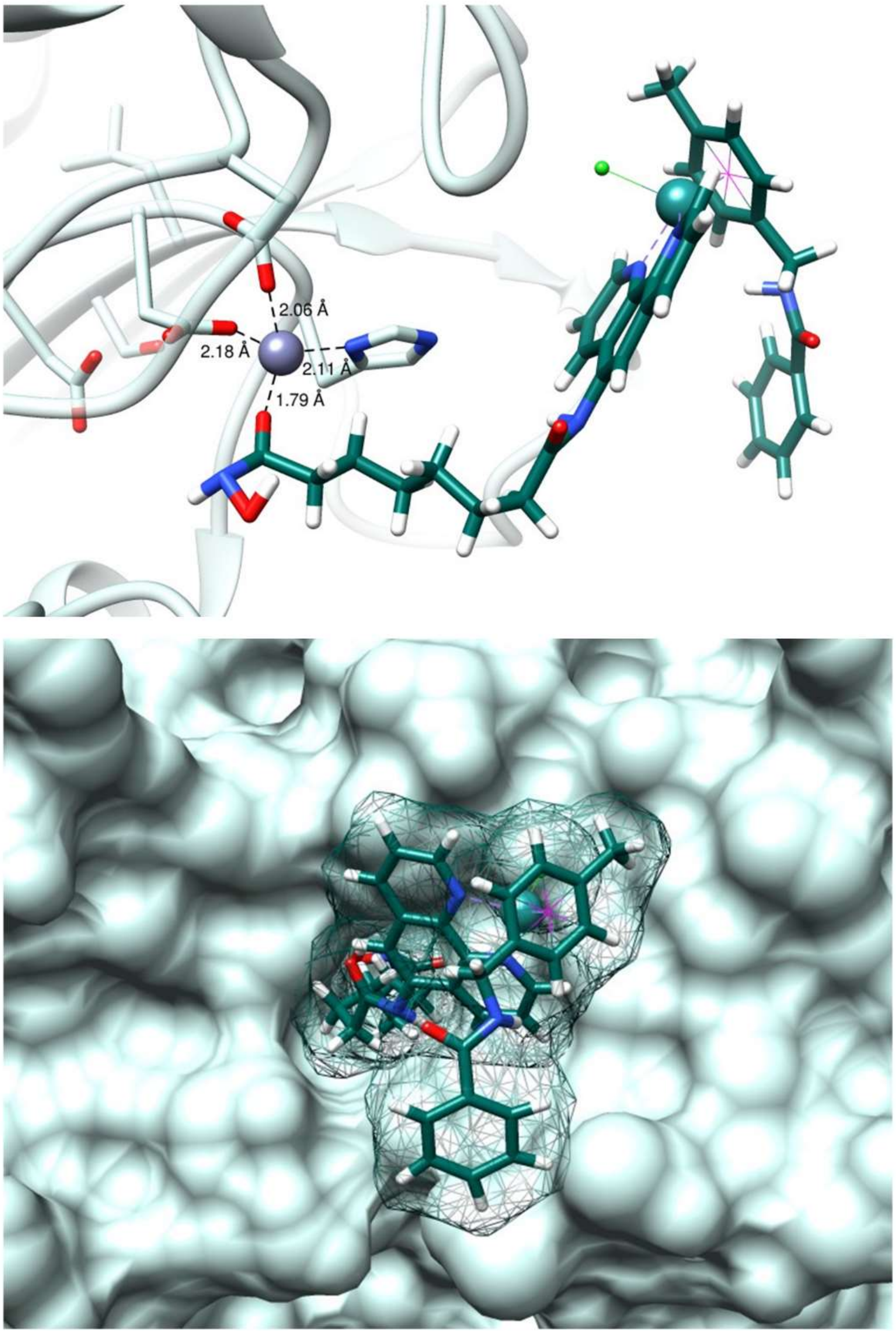


Fig S19. Computational docking study showing *upper*: Zn(II) coordination view of complex **8f** with HDAC6; *lower*: active site cavity entrance surface view of complex **8f** with HDAC6.

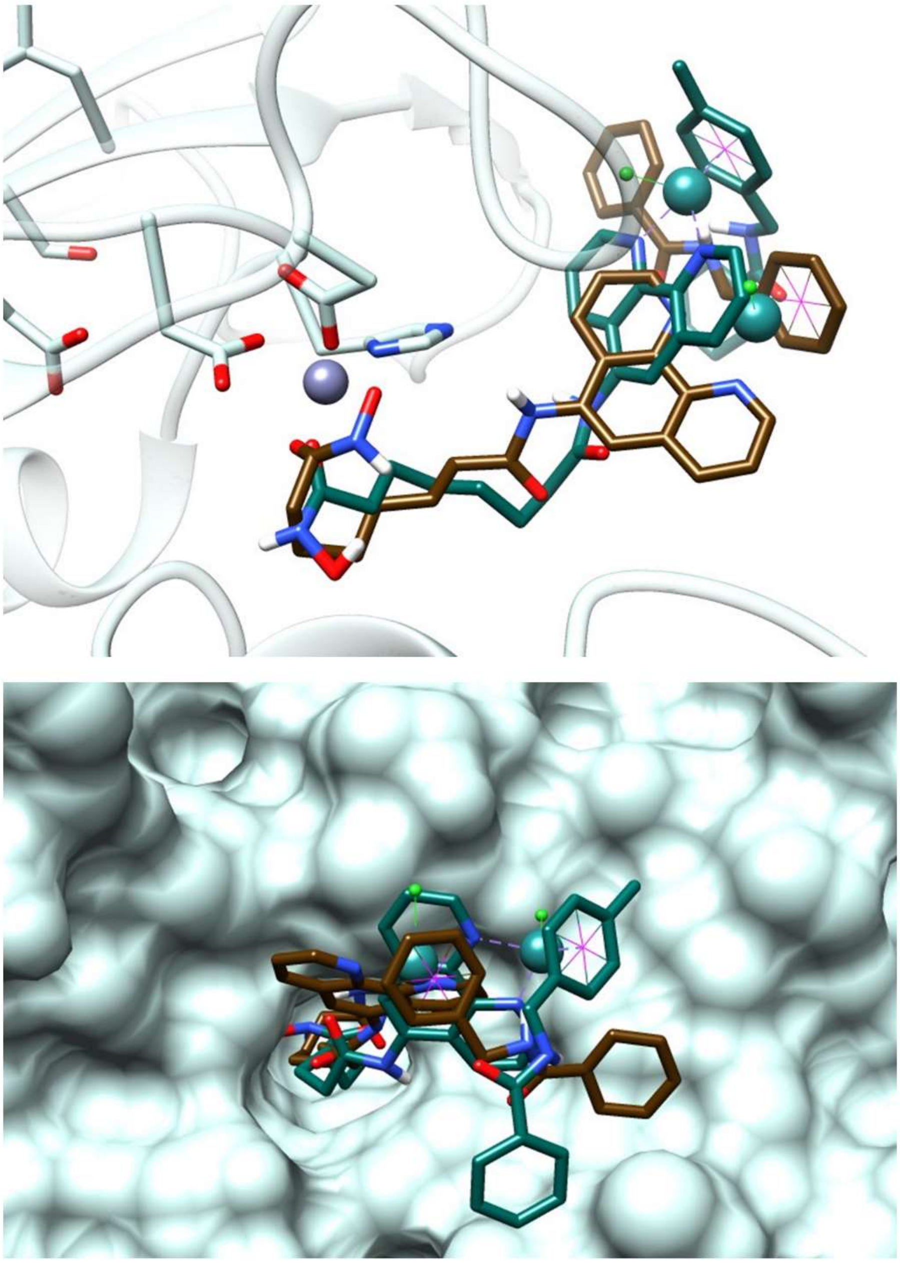

Fig S20. Computational docking study showing *upper* Zn(II) coordination view of **8c** and **8f** overlapped with HDAC6; *lower*: active site cavity entrance surface view of **8c** and **8f** overlapped with HDAC6.

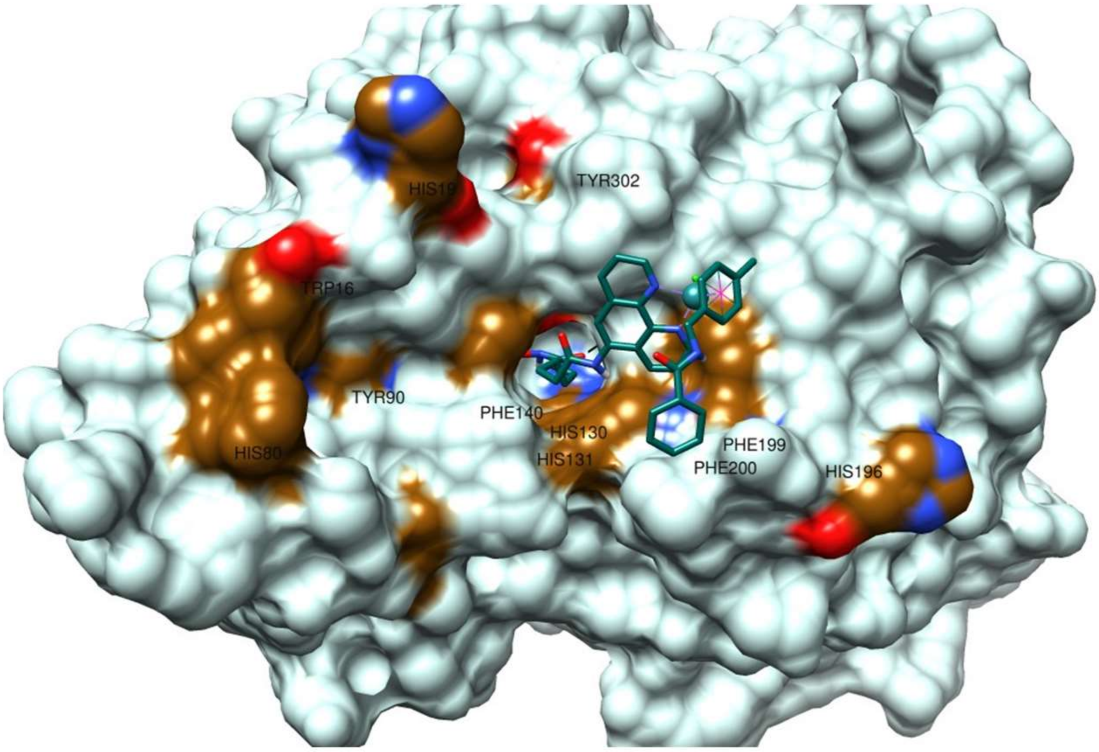


Fig S21. Computational docking study showing surface view of HDAC6 with complex **8f**. Highlighted gold are aromatic amino acids that have the potential to interact with the phenyl group of complex **8f**.

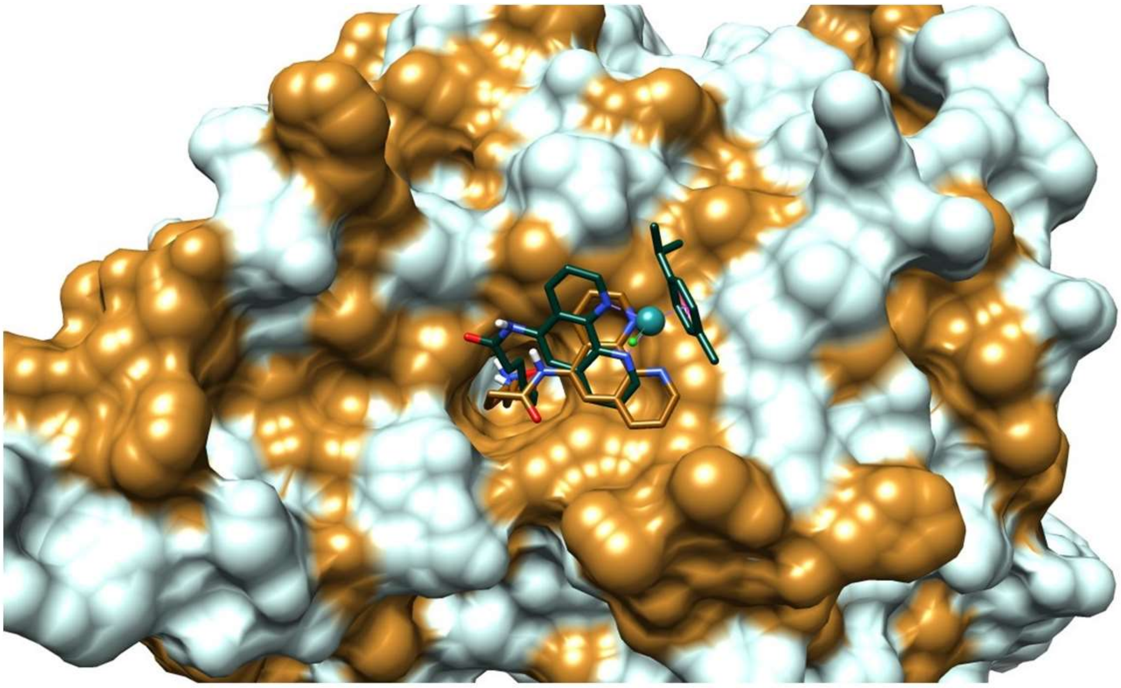

Fig S22. Computational docking study showing surface view of HDAC6 with ligand **L**[1] and complex **1**. Highlighted in gold are the hydrophobic region of the surface, showing the potential for the capping *p*-cymene group of **1** to interact in these regions.

## 9. NMR Spectra

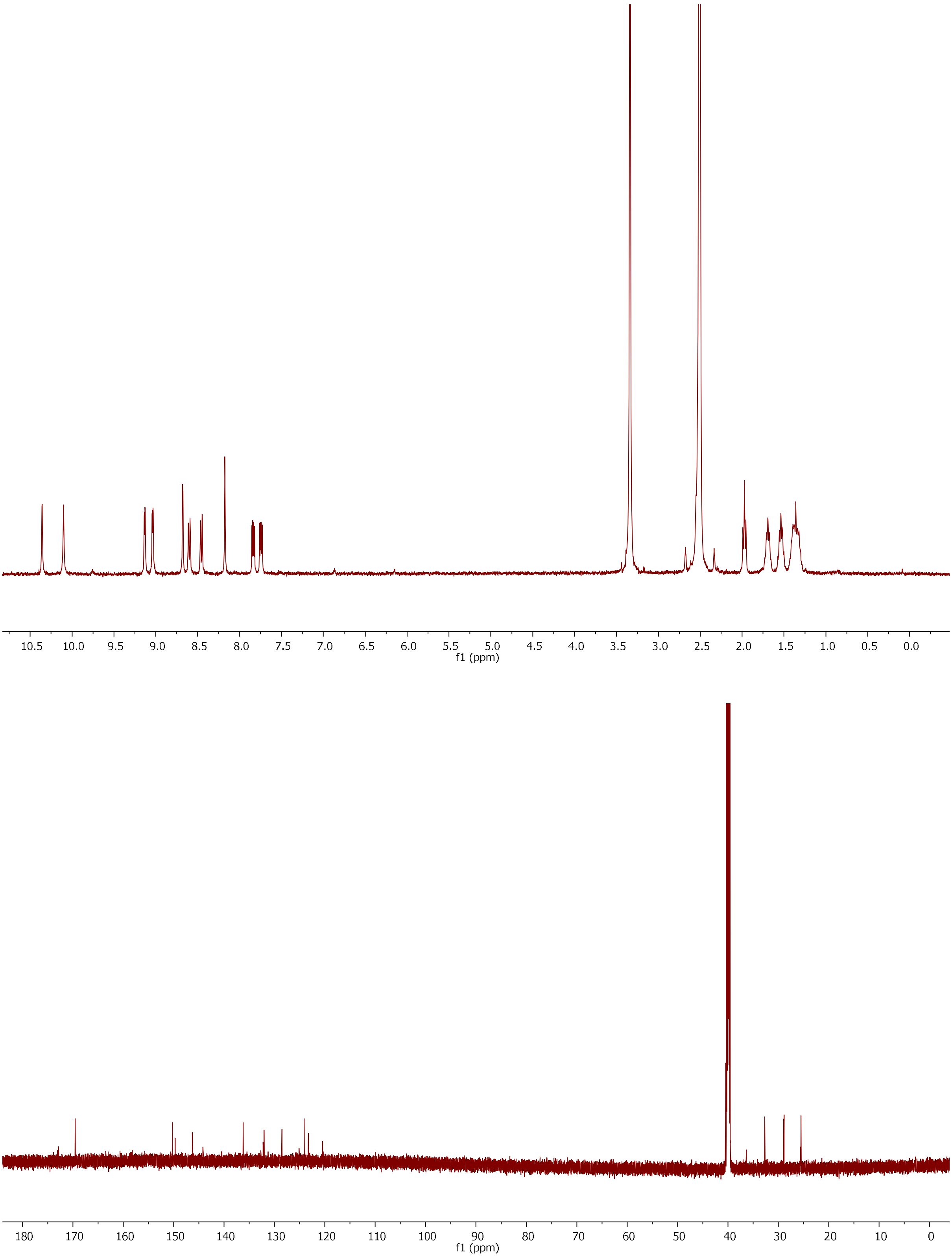


Fig S23. $^{1}$H-NMR spectrum (*upper*, DMSO-d$_6$, 700 MHz, 298 K) and $^{13}$C-NMR spectrum (*lower*, DMSO-d$_6$, 175 MHz, 298 K) of ligand **L$^1$**.

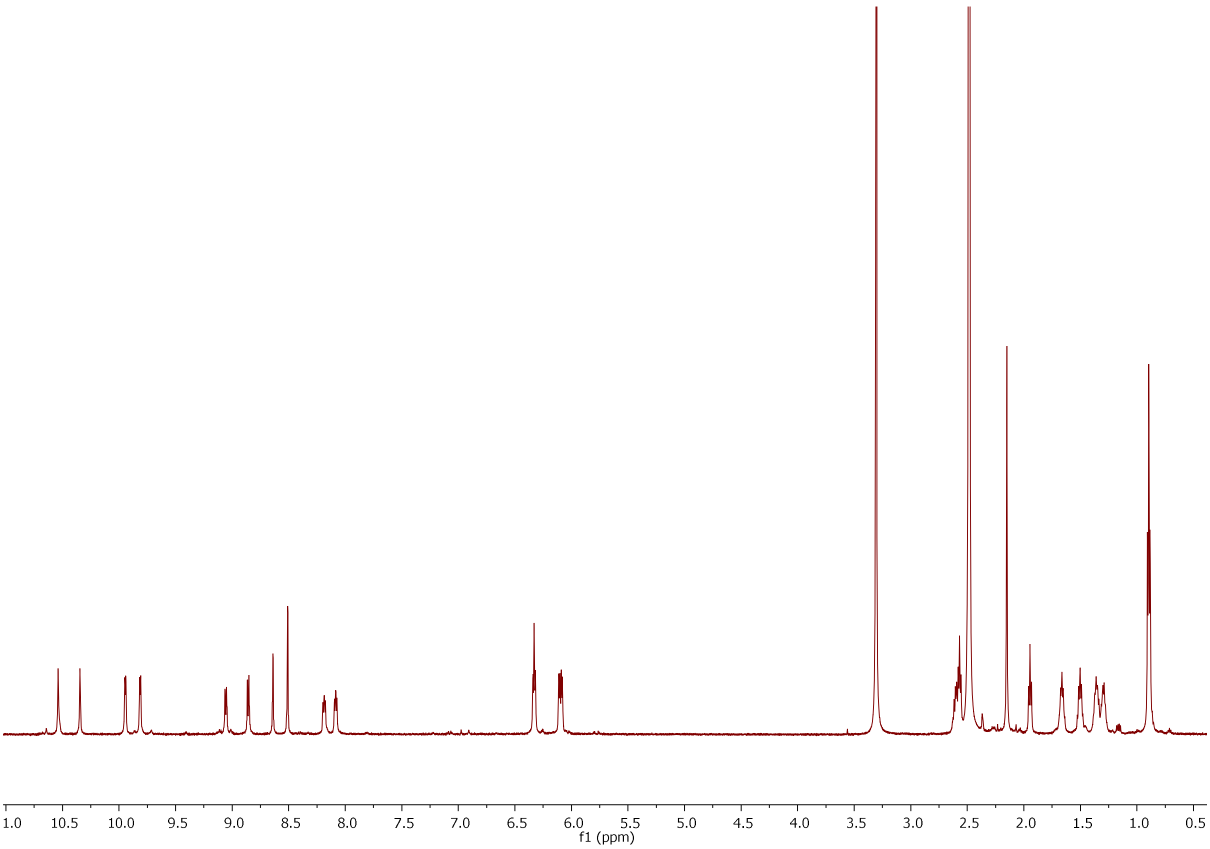


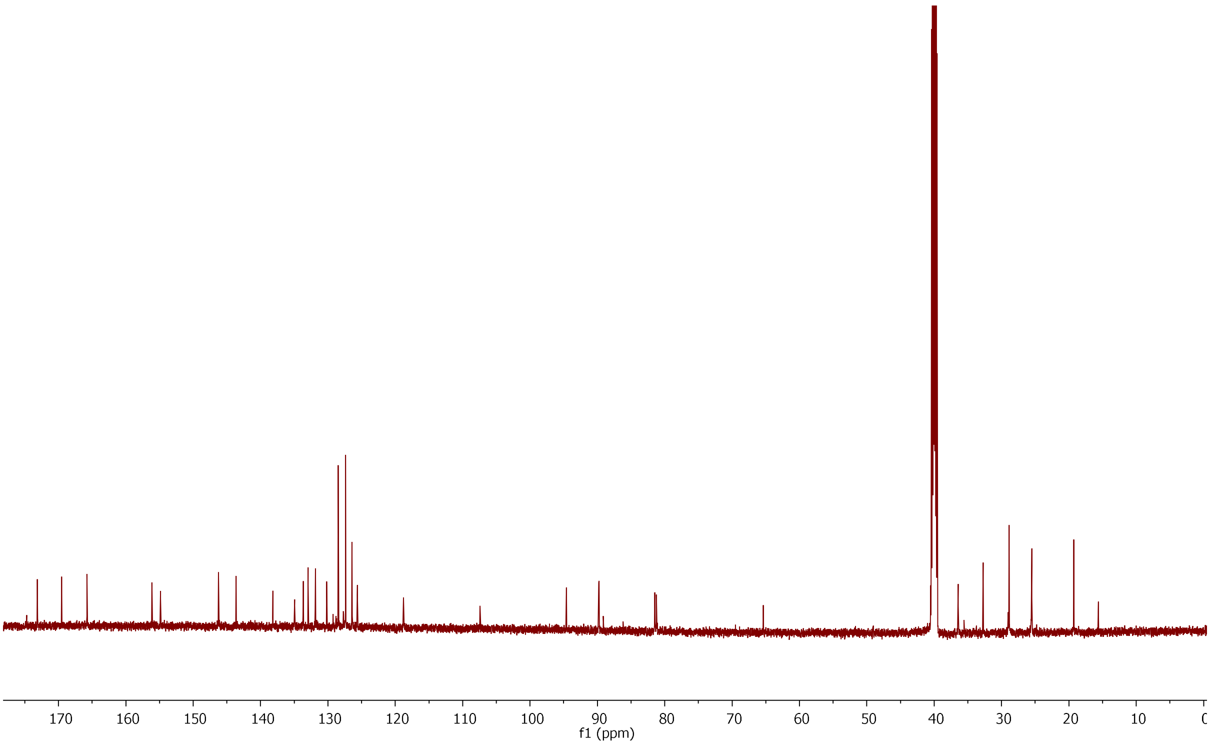


Fig S24. $^{1}$H-NMR spectrum (*upper*, DMSO-d$_6$, 700 MHz, 298 K) and $^{13}$C-NMR spectrum (*lower*, DMSO-d$_6$, 175 MHz, 298 K) of complex **1**.

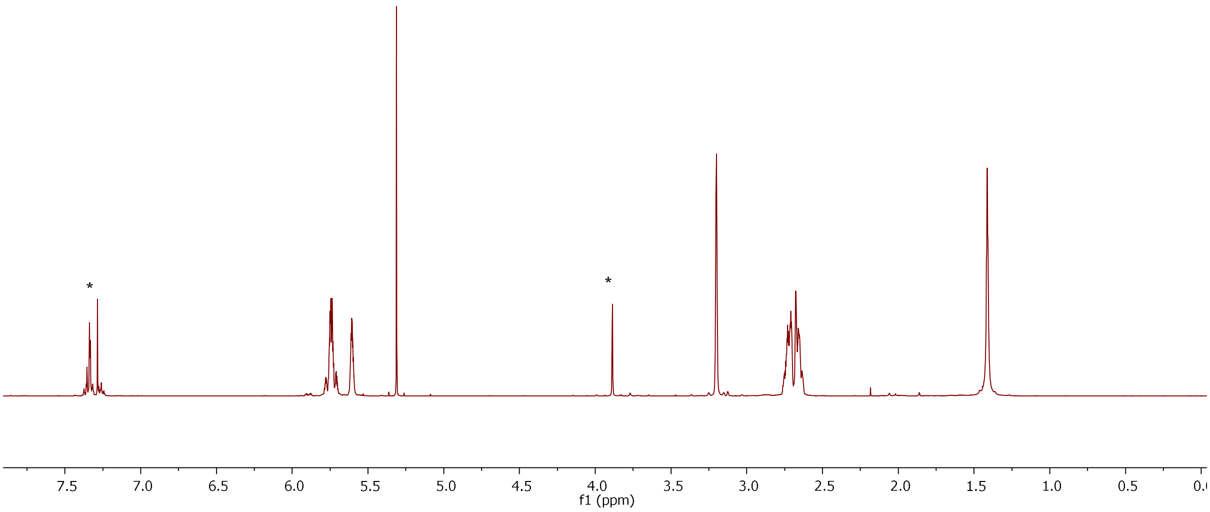


Fig S25. $^{1}$H-NMR spectrum ($CDCl_3$, 400 MHz, 298 K) of diene **4**. *denotes impurities from unreduced starting material.

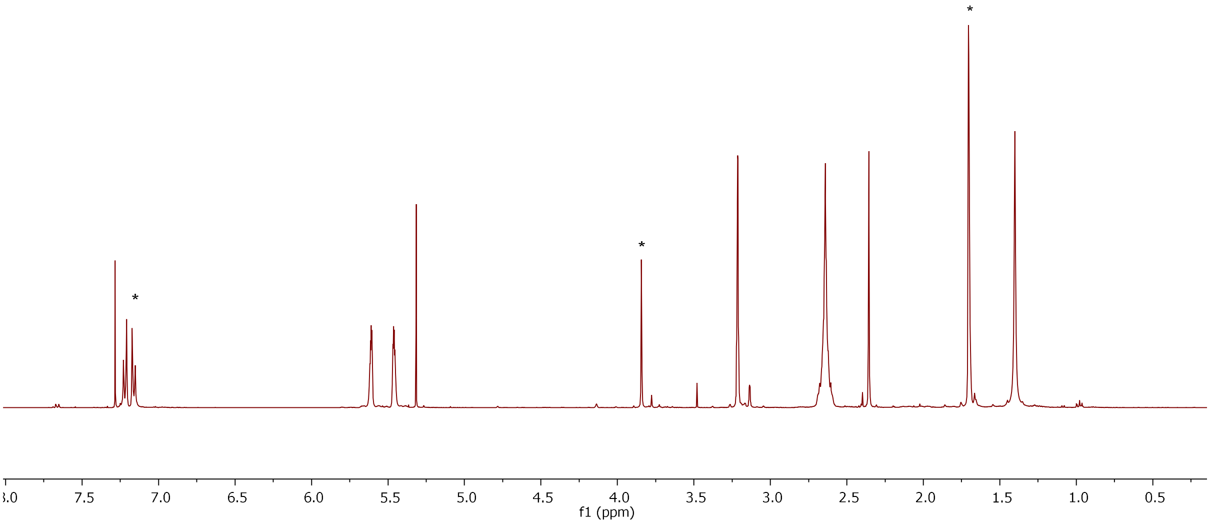


Fig S26. $^{1}$H-NMR spectrum ($CDCl_3$, 400 MHz, 298 K) of **5**. *denotes impurities from unreduced starting material

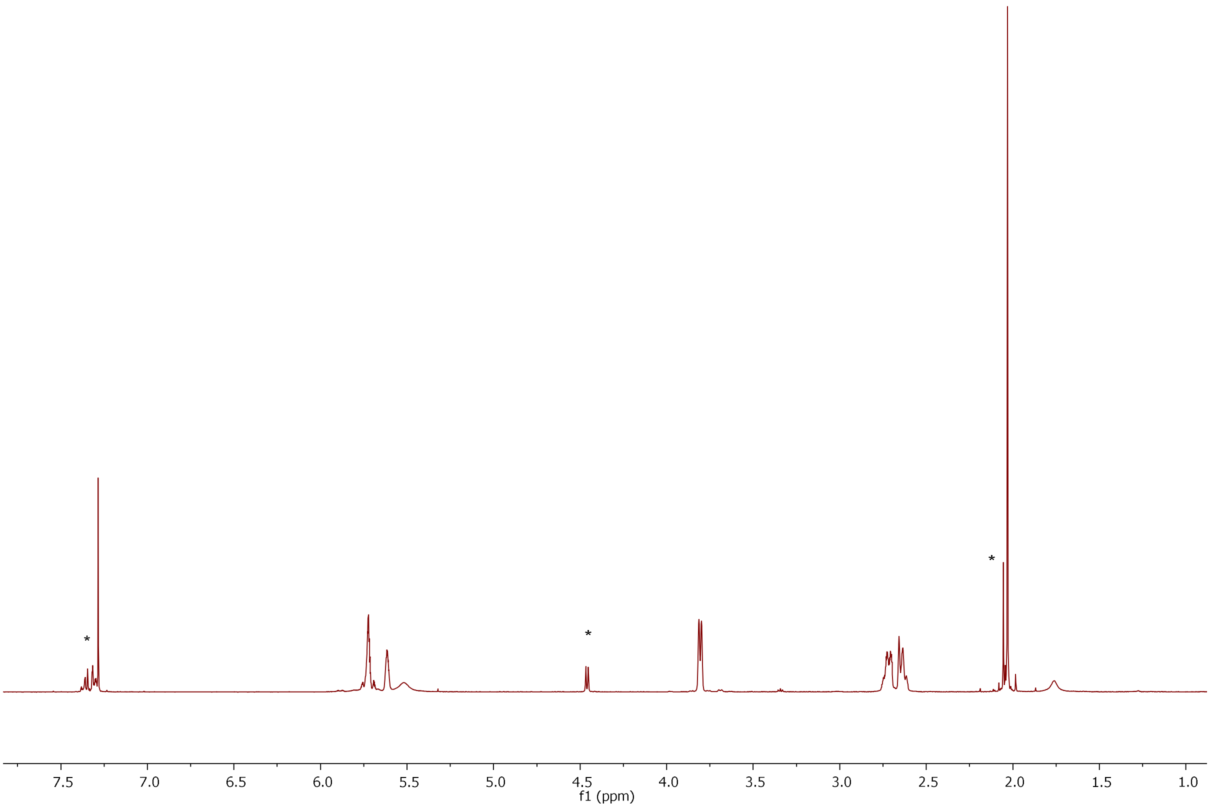


Fig S27. $^{1}$H-NMR spectrum ($CDCl_3$, 400 MHz, 298 K) of crude **6a**, used without purification.

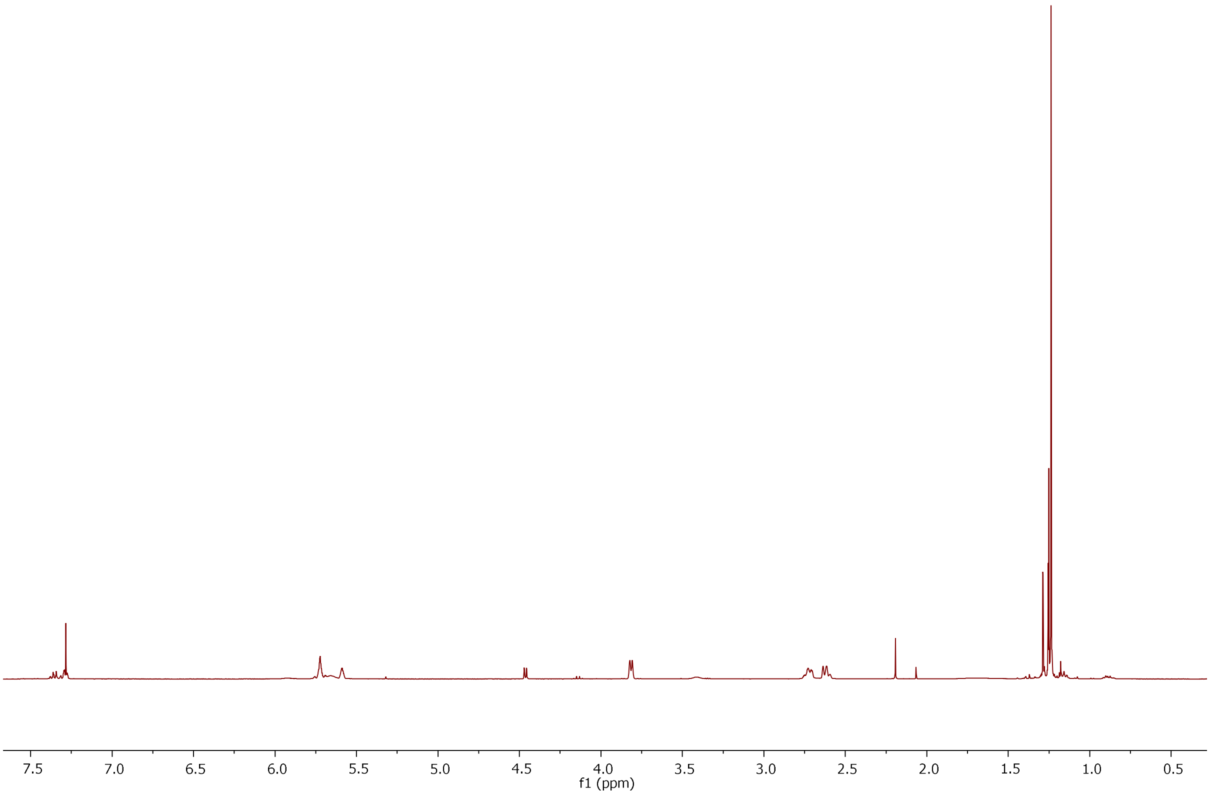


Fig S28. $^{1}$H-NMR spectrum ($CDCl_3$, 400 MHz, 298 K) of crude **6b**, used without purification.

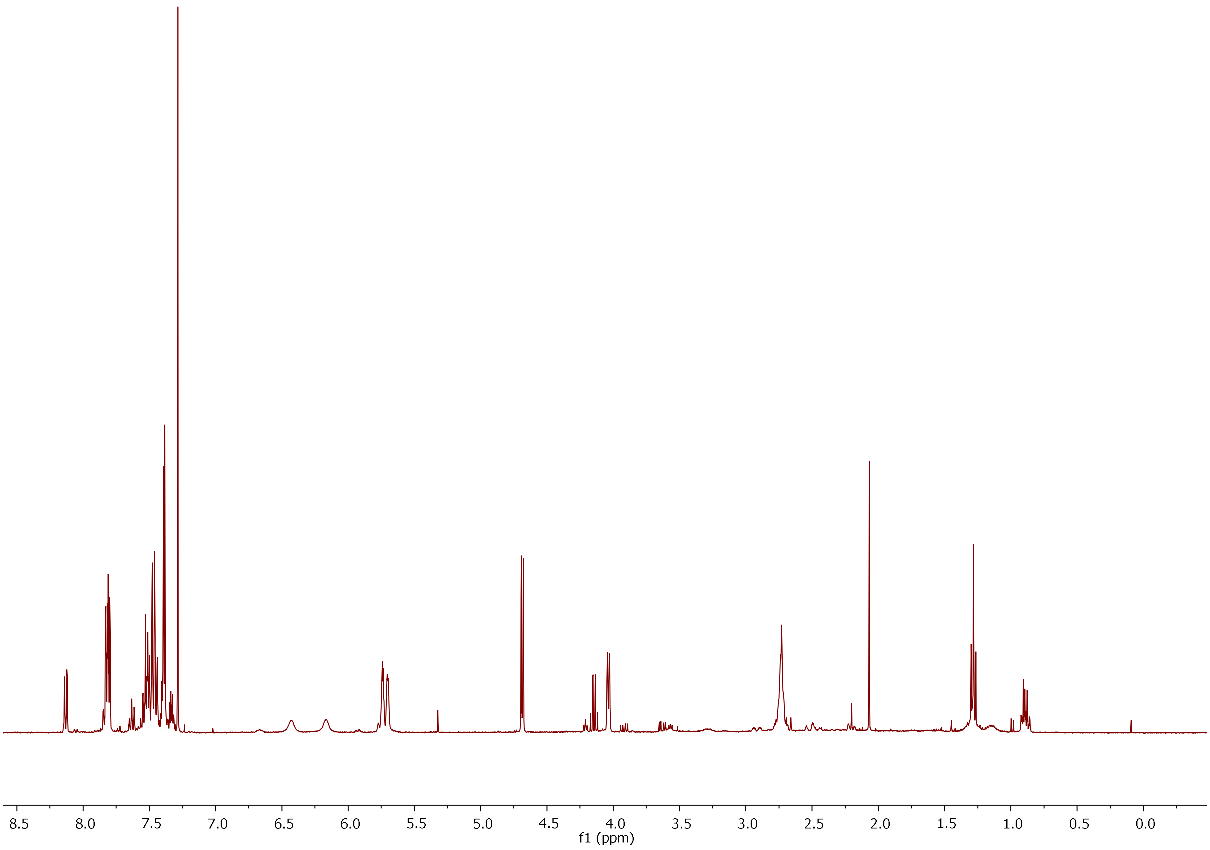


Fig S29. $^{1}$H-NMR spectrum ($CDCl_3$, 400 MHz, 298 K) of crude **6c**, used without purification.

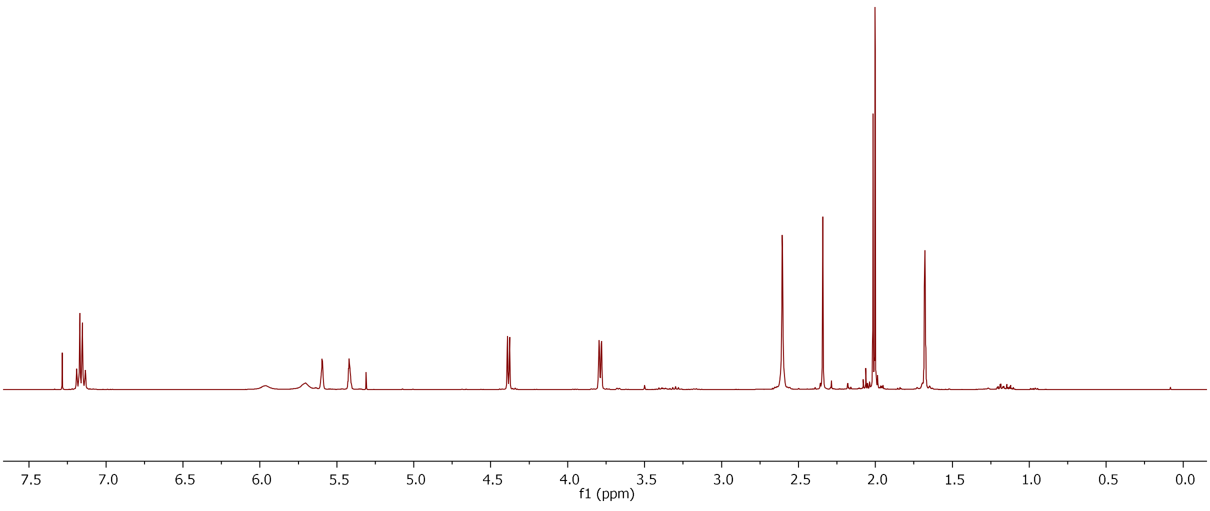


Fig S30. $^{1}$H-NMR spectrum ($CDCl_3$, 400 MHz, 298 K) of crude **6d**, used without purification.

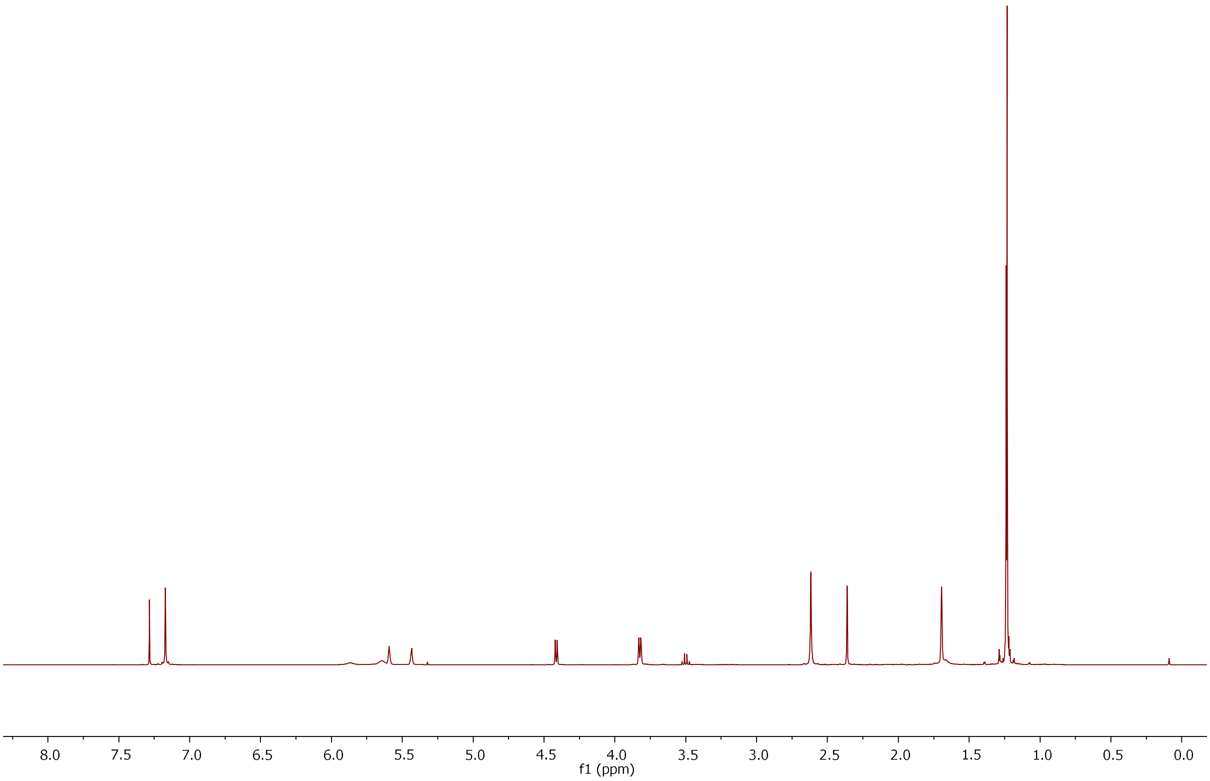


Fig S31. $^{1}$H-NMR spectrum ($CDCl_3$, 400 MHz, 298 K) of crude **6e**, used without purification.

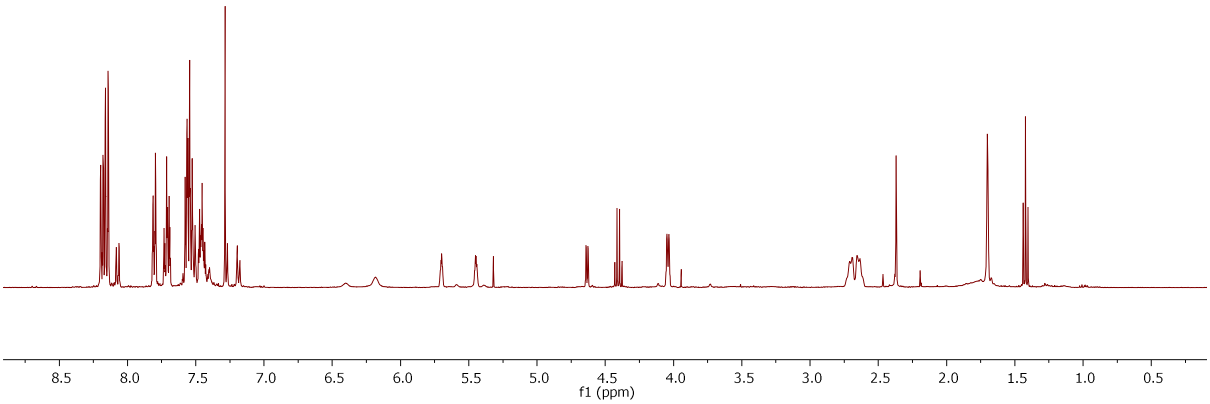


Fig S32. $^{1}$H-NMR spectrum ($CDCl_3$, 400 MHz, 298 K) of crude **6f**, used without purification.

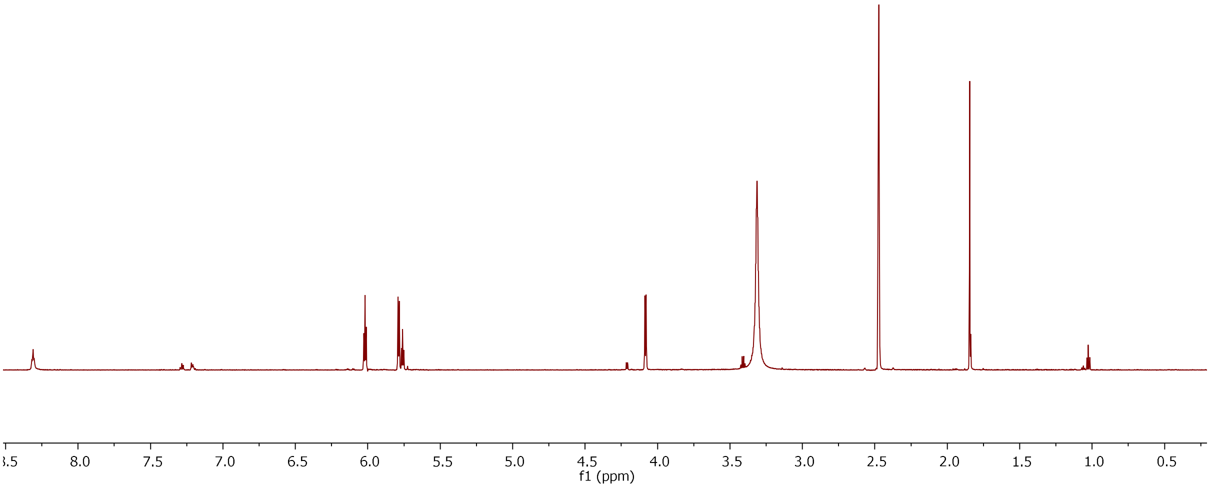


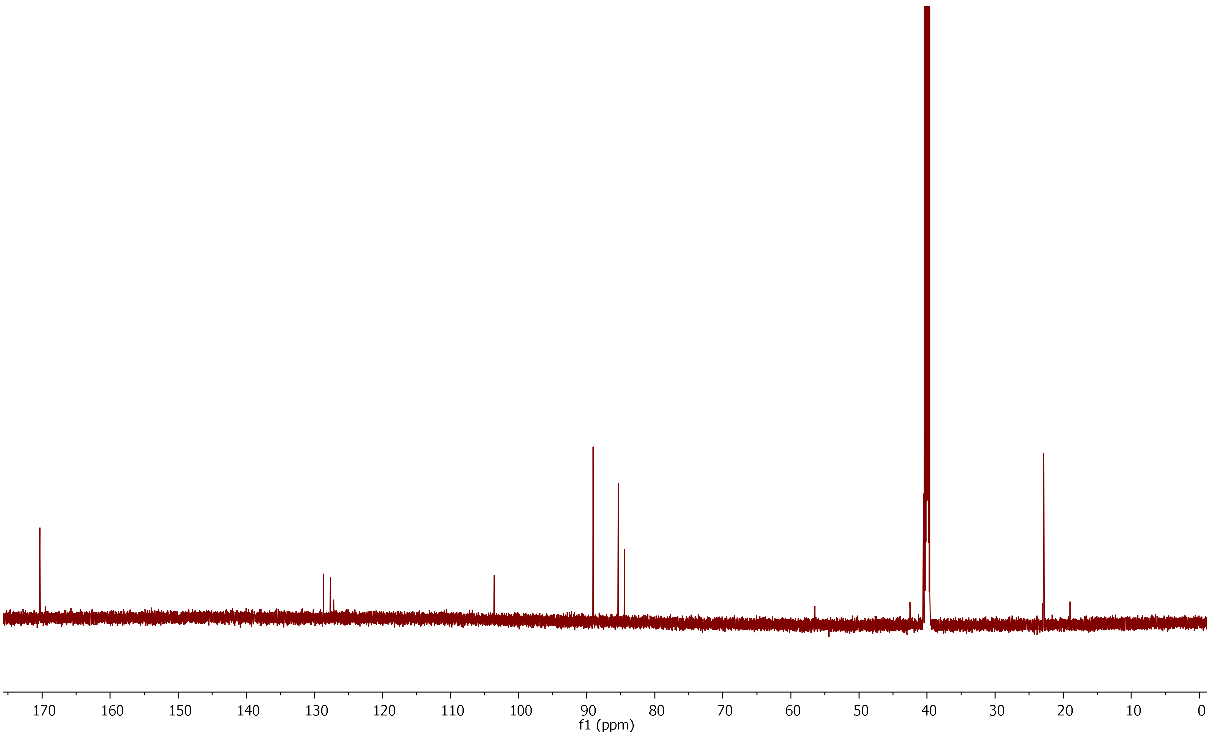


Fig S33. $^{1}$H-NMR spectrum (*upper*, DMSO-$d_6$, 700 MHz, 298 K) and $^{13}$C-NMR spectrum (*lower*, DMSO-$d_6$, 175 MHz, 298 K) of dimer **7a**

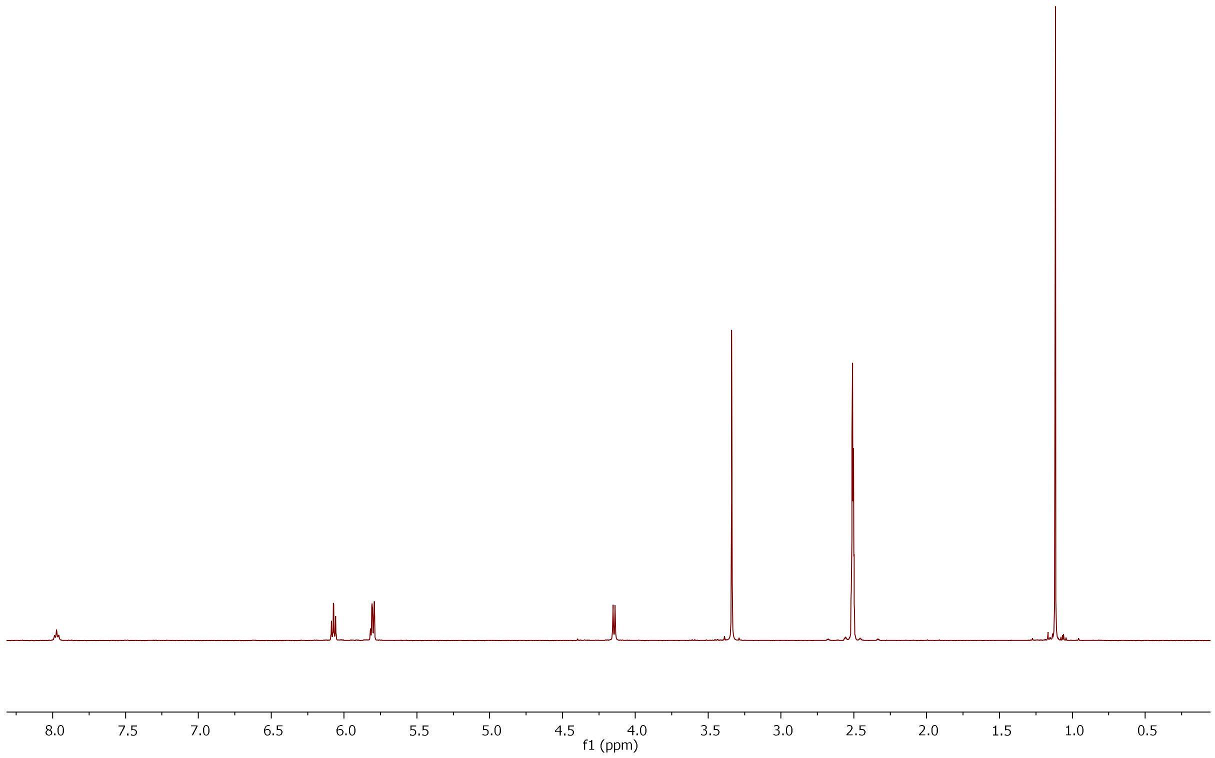


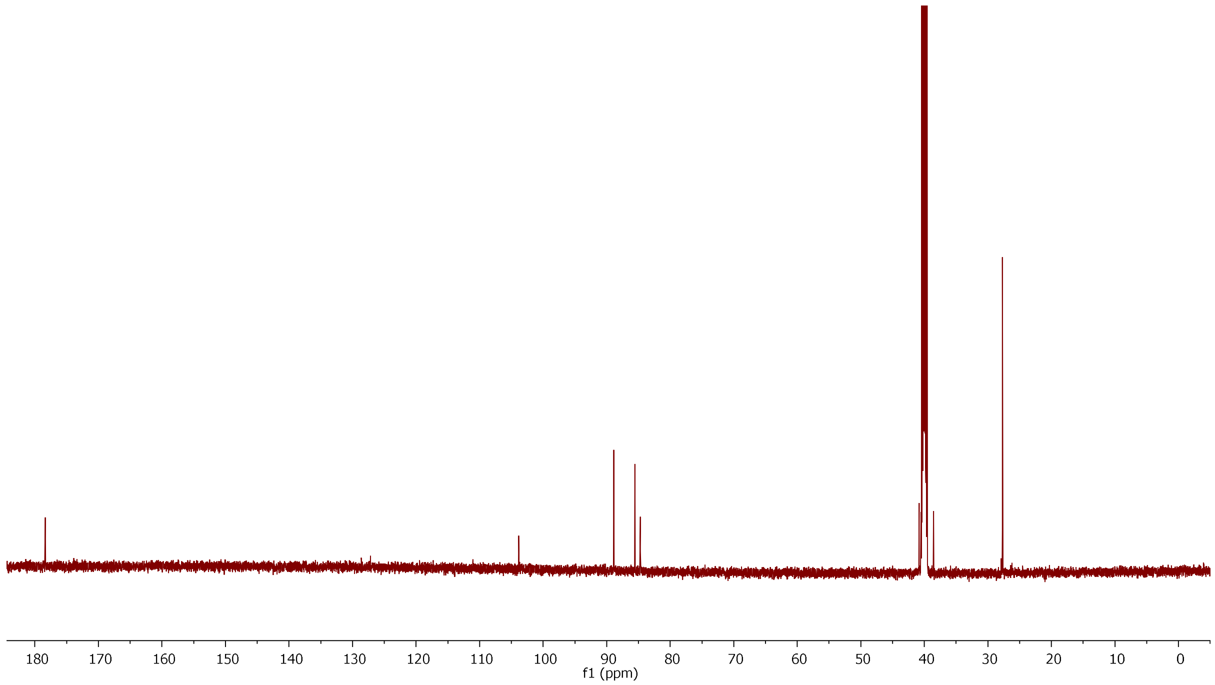


Fig S34. $^{1}$H-NMR spectrum (*upper*, DMSO-$d_6$, 700 MHz, 298 K) and $^{13}$C-NMR spectrum (*lower*, DMSO-$d_6$, 175 MHz, 298 K) of dimer **7b**

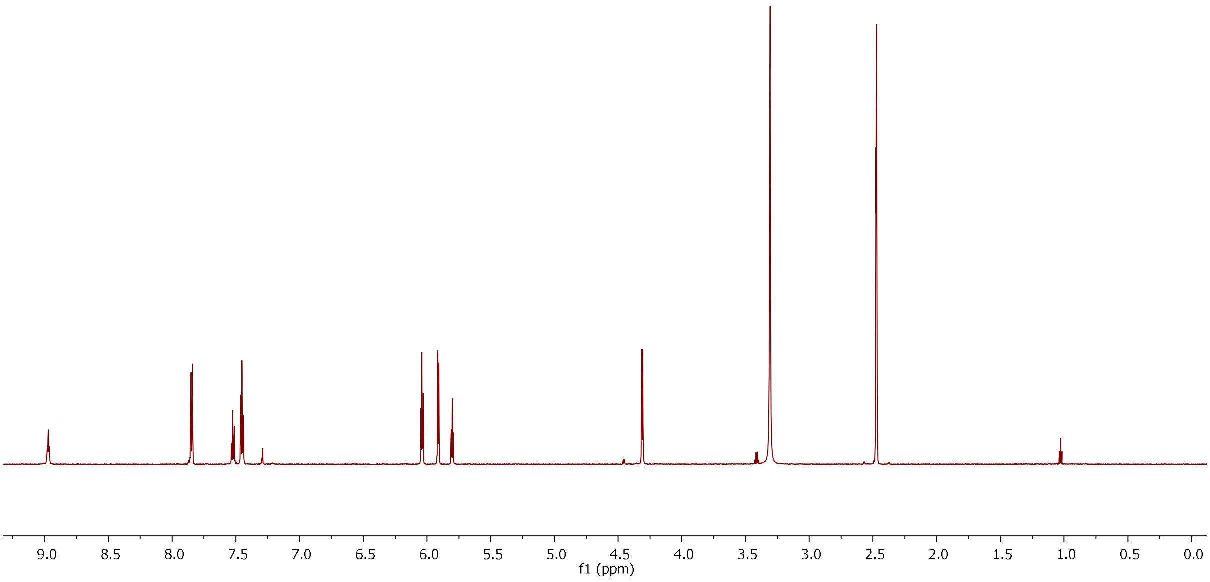


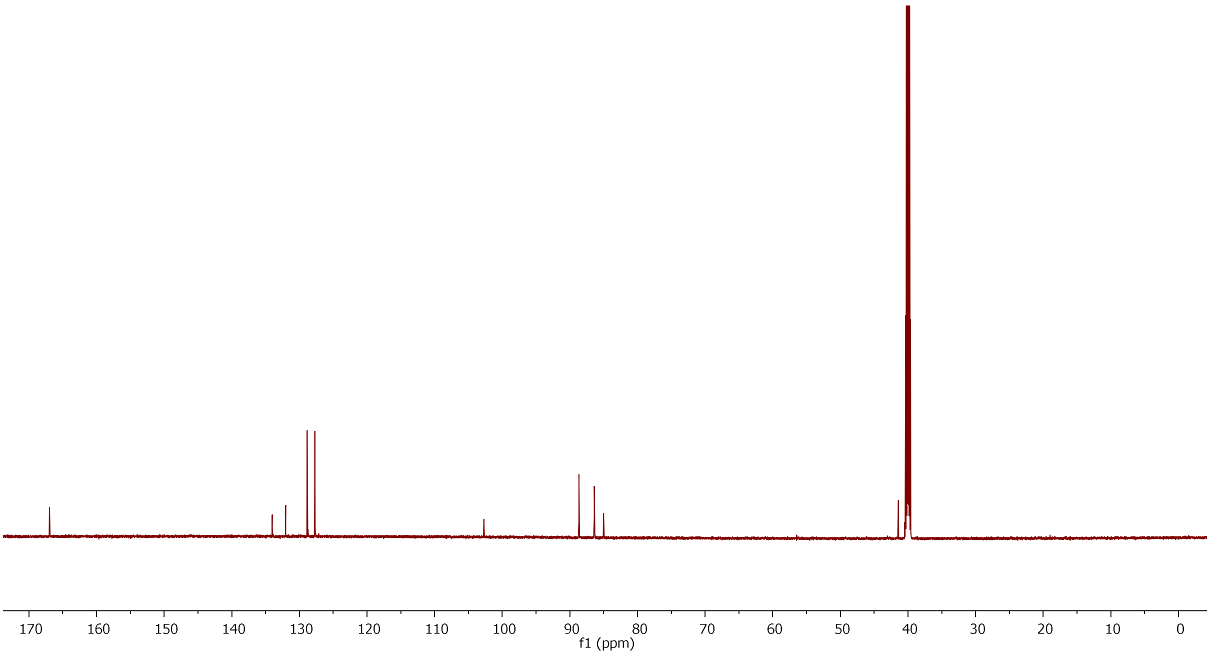


Fig S35. $^{1}$H-NMR spectrum (*upper*, DMSO-$d_6$, 700 MHz, 298 K) and $^{13}$C-NMR spectrum (*lower*, DMSO-$d_6$, 175 MHz, 298 K) of dimer **7c**

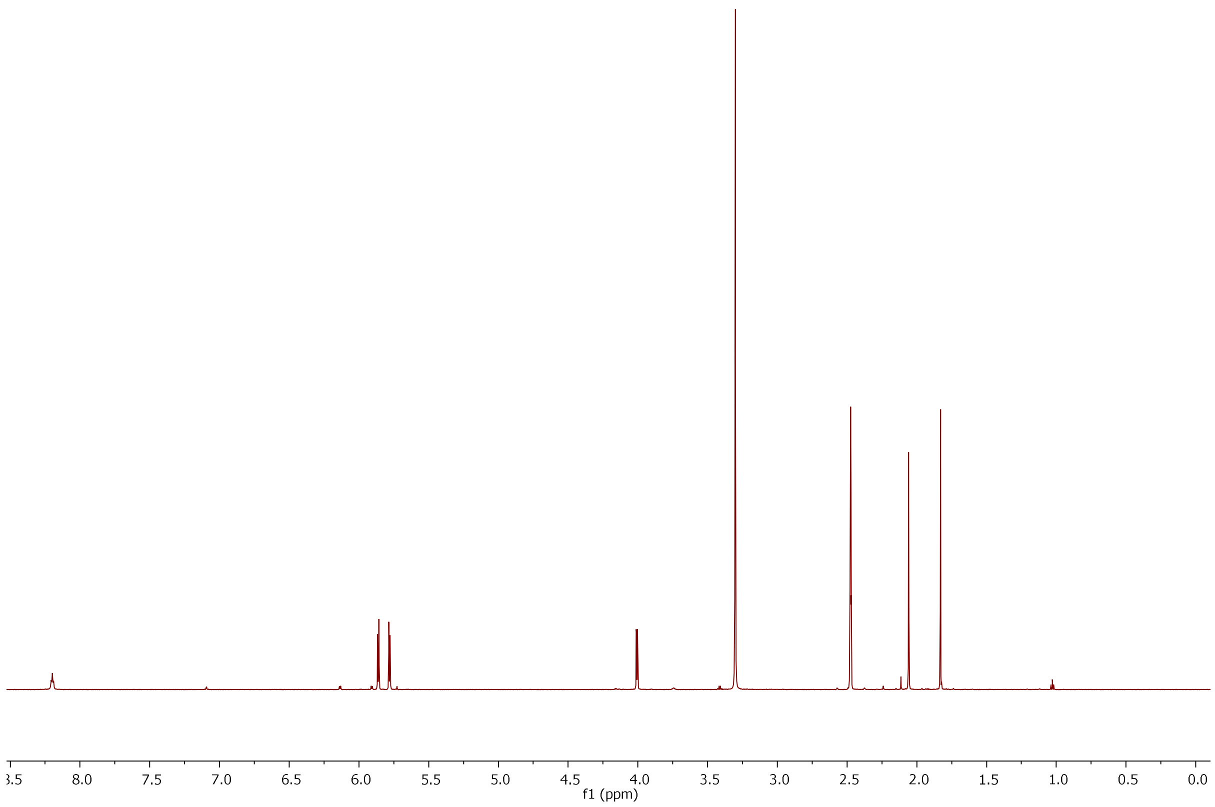

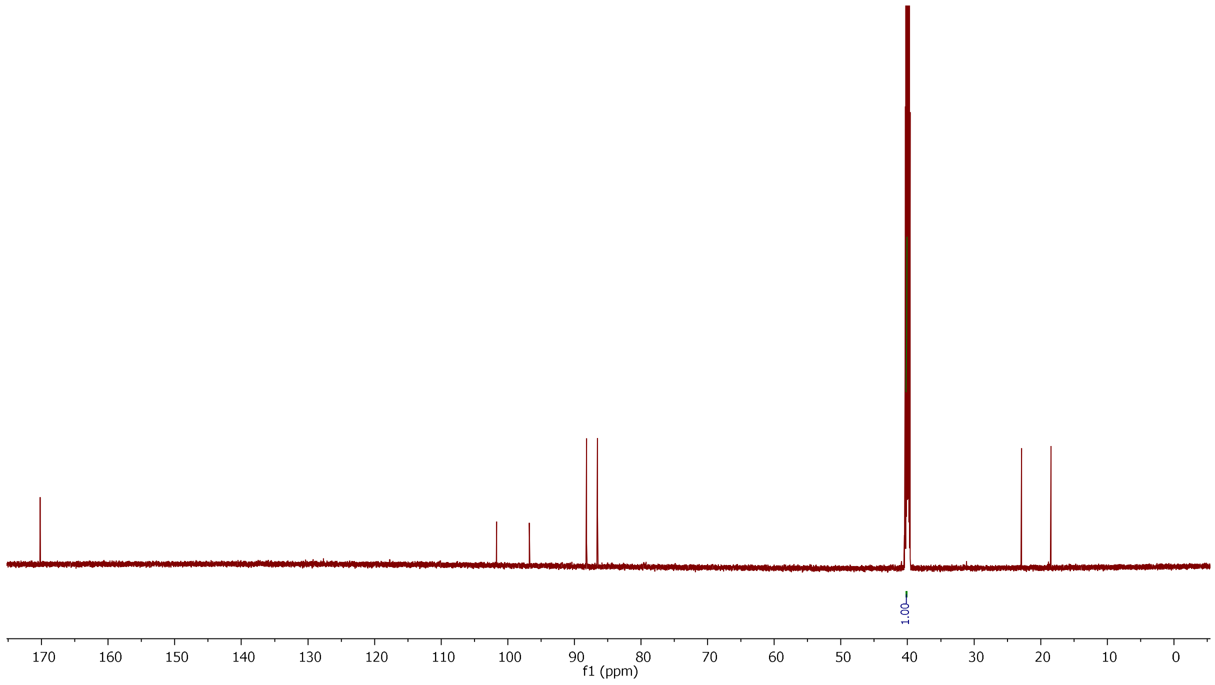

Fig S36. $^{1}$H-NMR spectrum (*upper*, DMSO-d$_6$, 700 MHz, 298 K) and $^{13}$C-NMR spectrum (*lower*, DMSO-d$_6$, 175 MHz, 298 K) of dimer **7d**

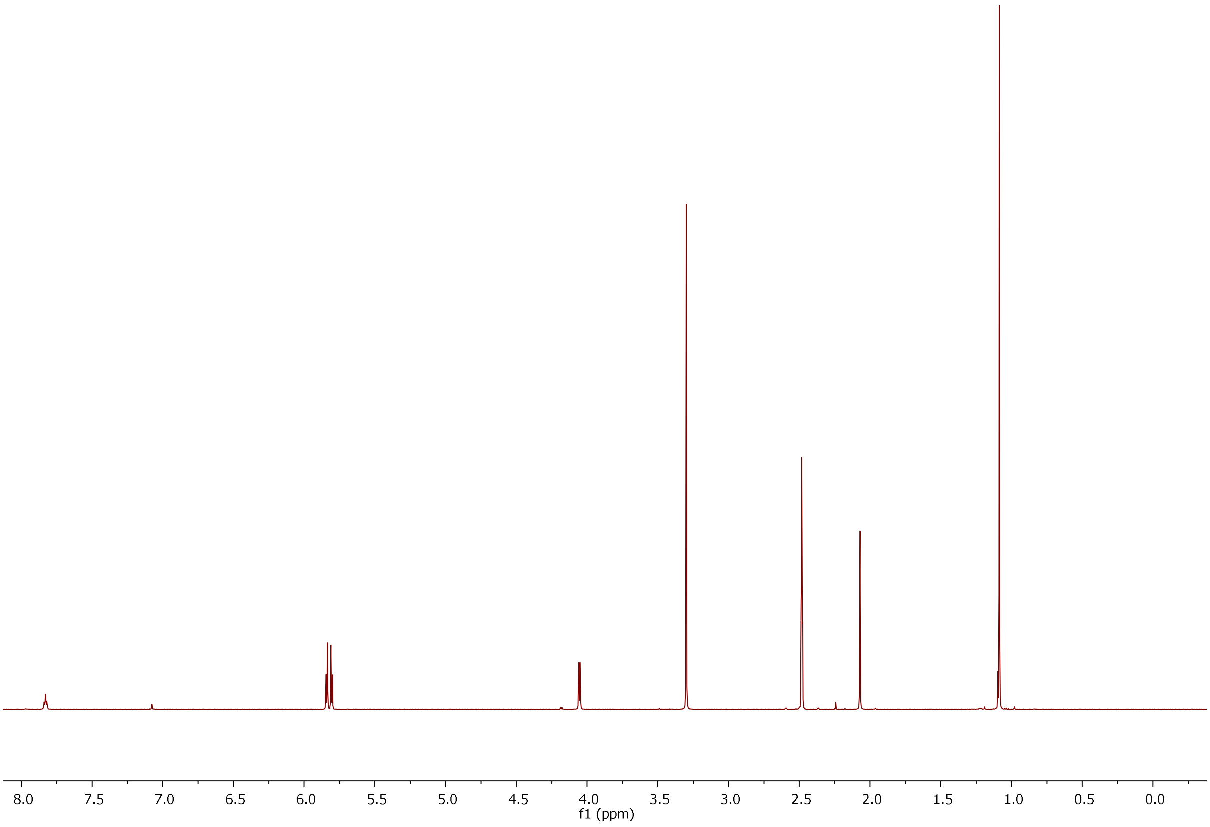


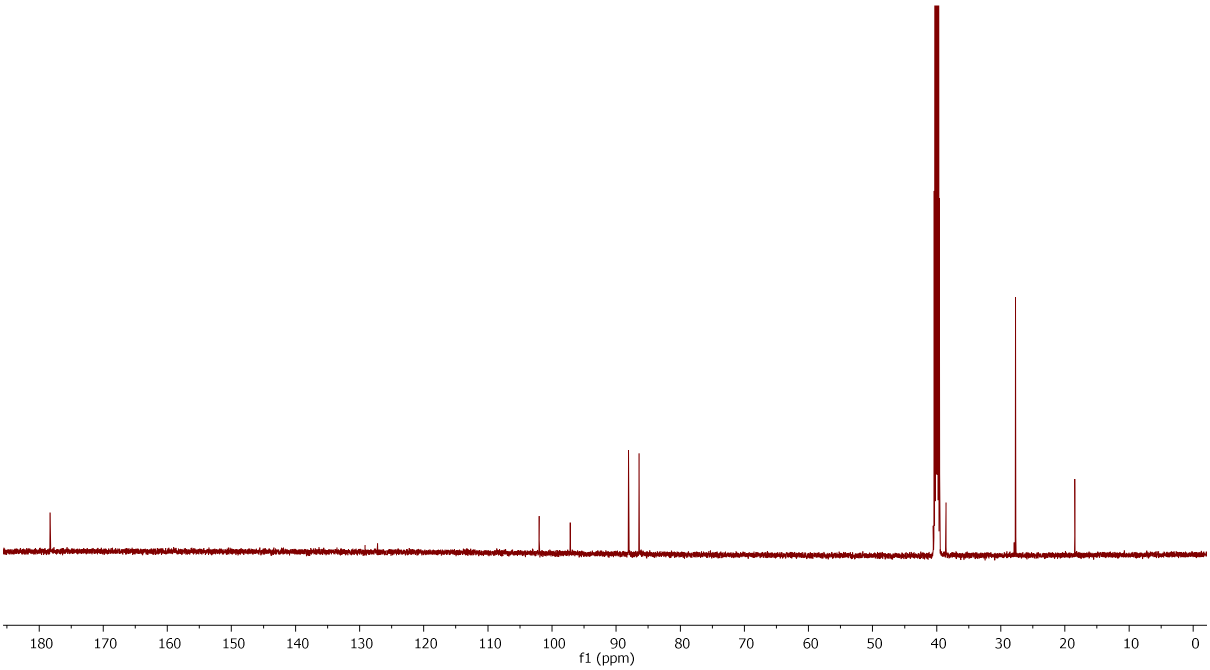


Fig S37. $^{1}$H-NMR spectrum (*upper*, DMSO-d$_6$, 700 MHz, 298 K) and $^{13}$C-NMR spectrum (*lower*, DMSO-d$_6$, 175 MHz, 298 K) of dimer **7e**

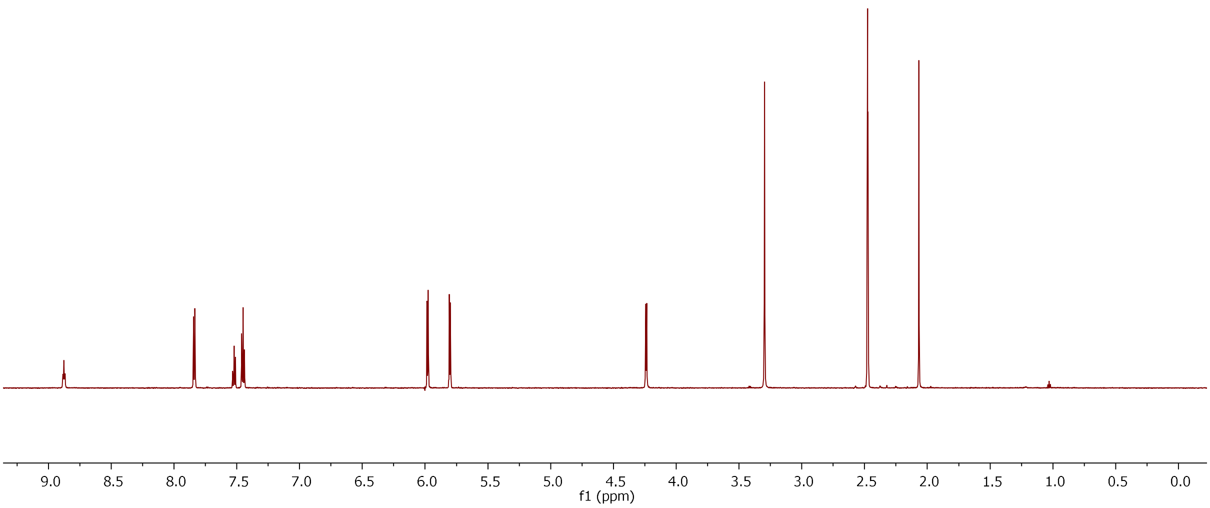


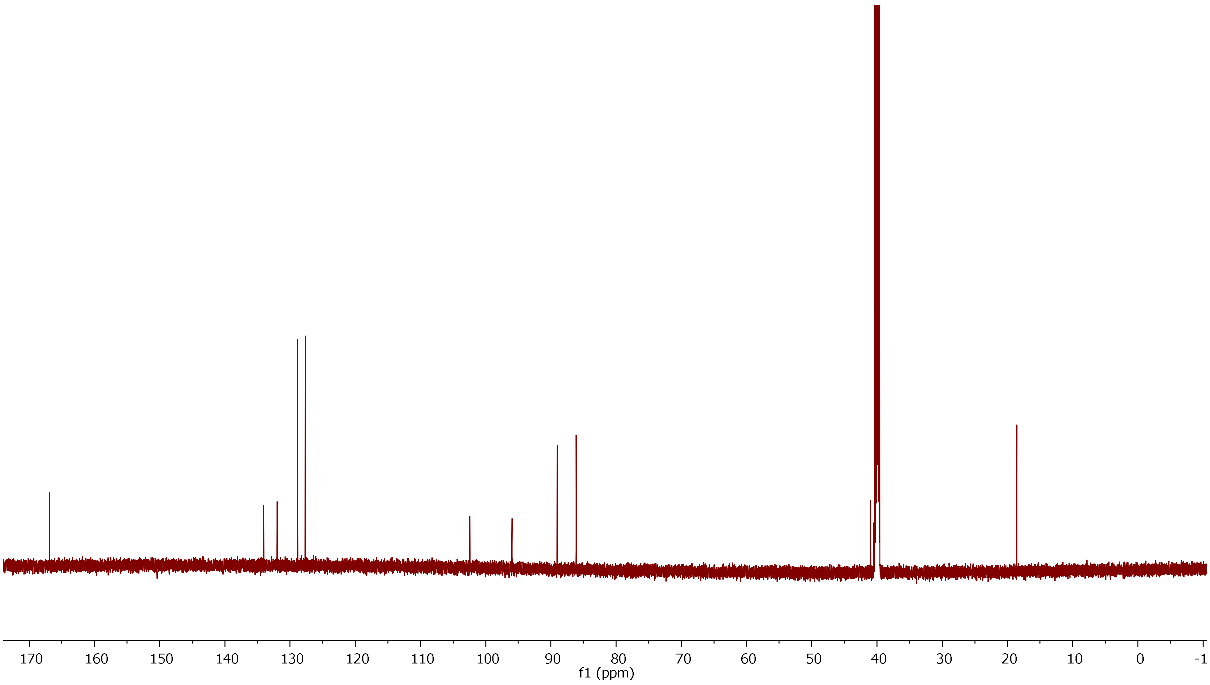


Fig S38. $^{1}$H-NMR spectrum (*upper*, DMSO-$d_6$, 700 MHz, 298 K) and $^{13}$C-NMR spectrum (*lower*, DMSO-$d_6$, 175 MHz, 298 K) of dimer **7f**

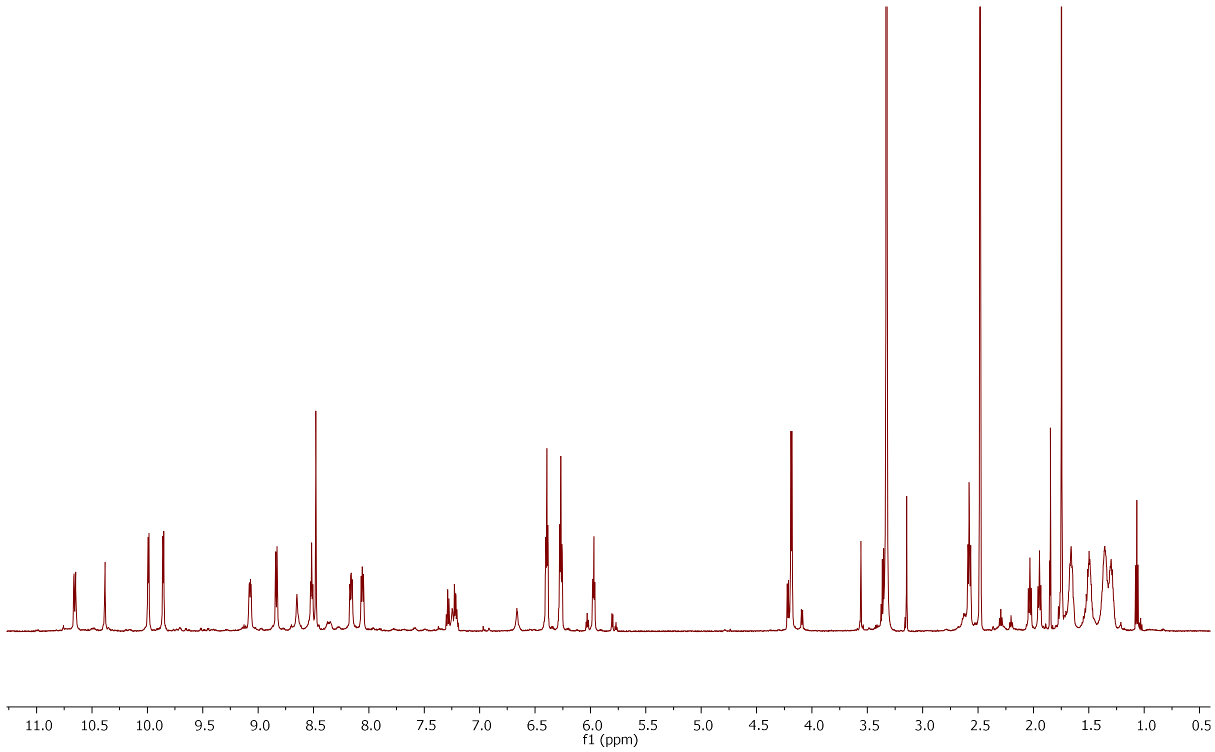


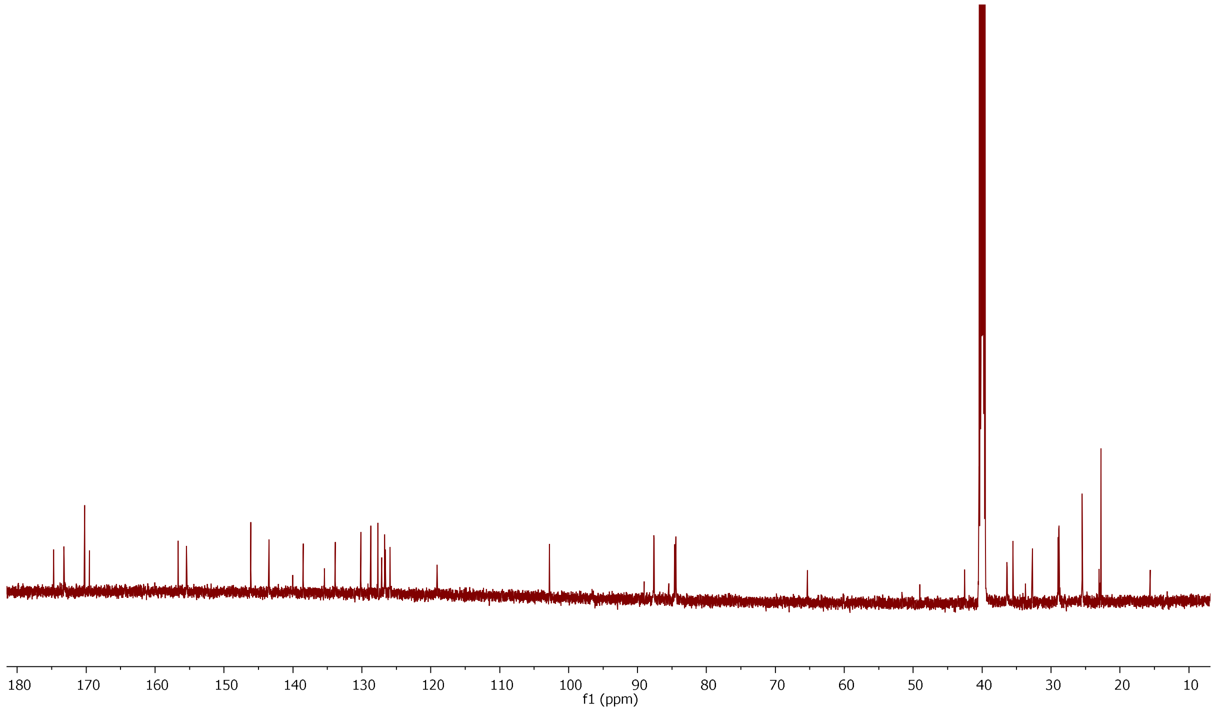


Fig S39. $^{1}$H-NMR spectrum (*upper*, DMSO-$d_6$, 700 MHz, 298 K) and $^{13}$C-NMR spectrum (*lower*, DMSO-$d_6$, 175 MHz, 298 K) of complex **8a**.

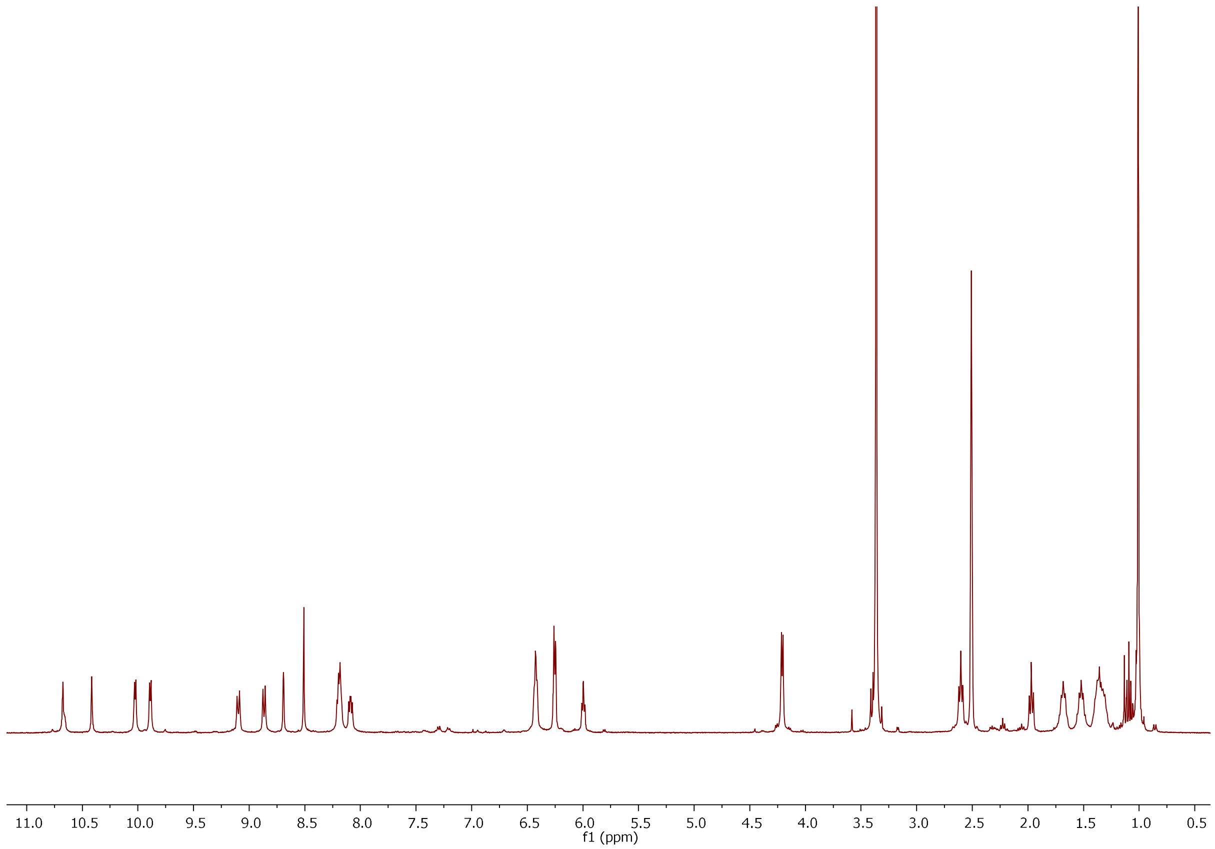


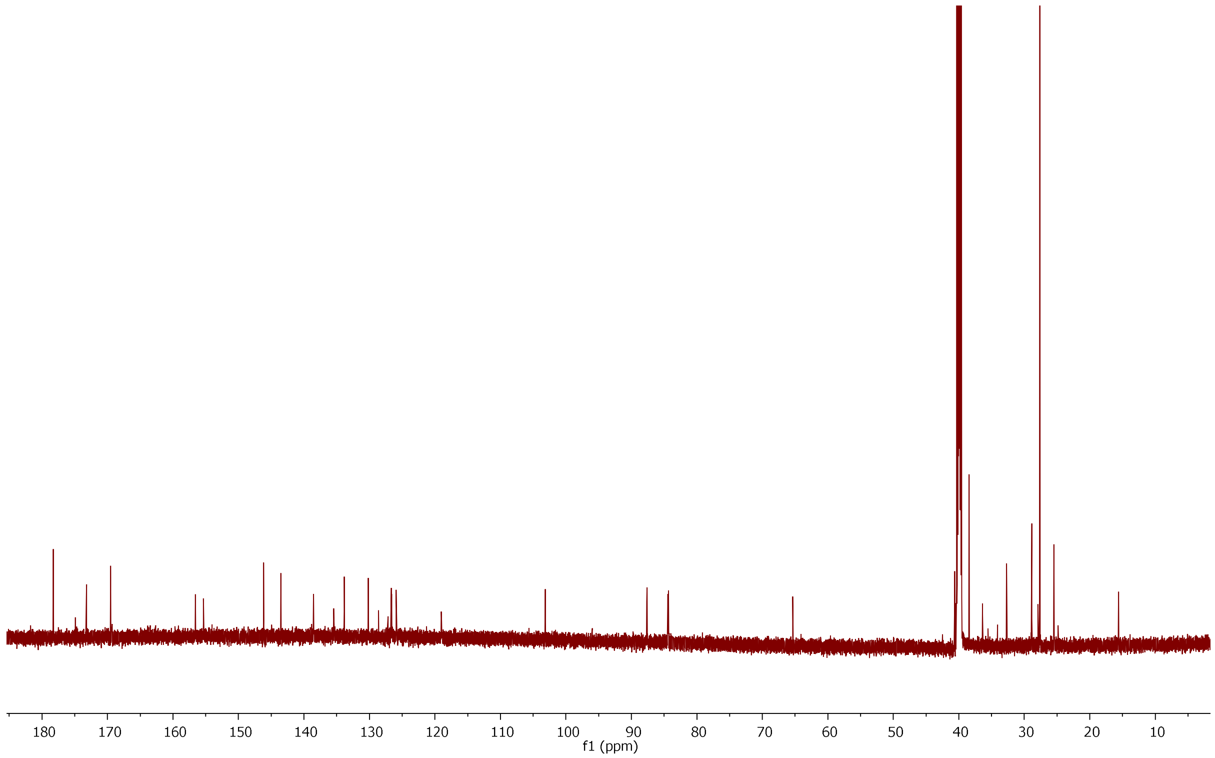


Fig S40. $^{1}$H-NMR spectrum (*upper*, DMSO-$d_6$, 700 MHz, 298 K) and $^{13}$C-NMR spectrum (*lower*, DMSO-$d_6$, 175 MHz, 298 K) of complex **8b**.

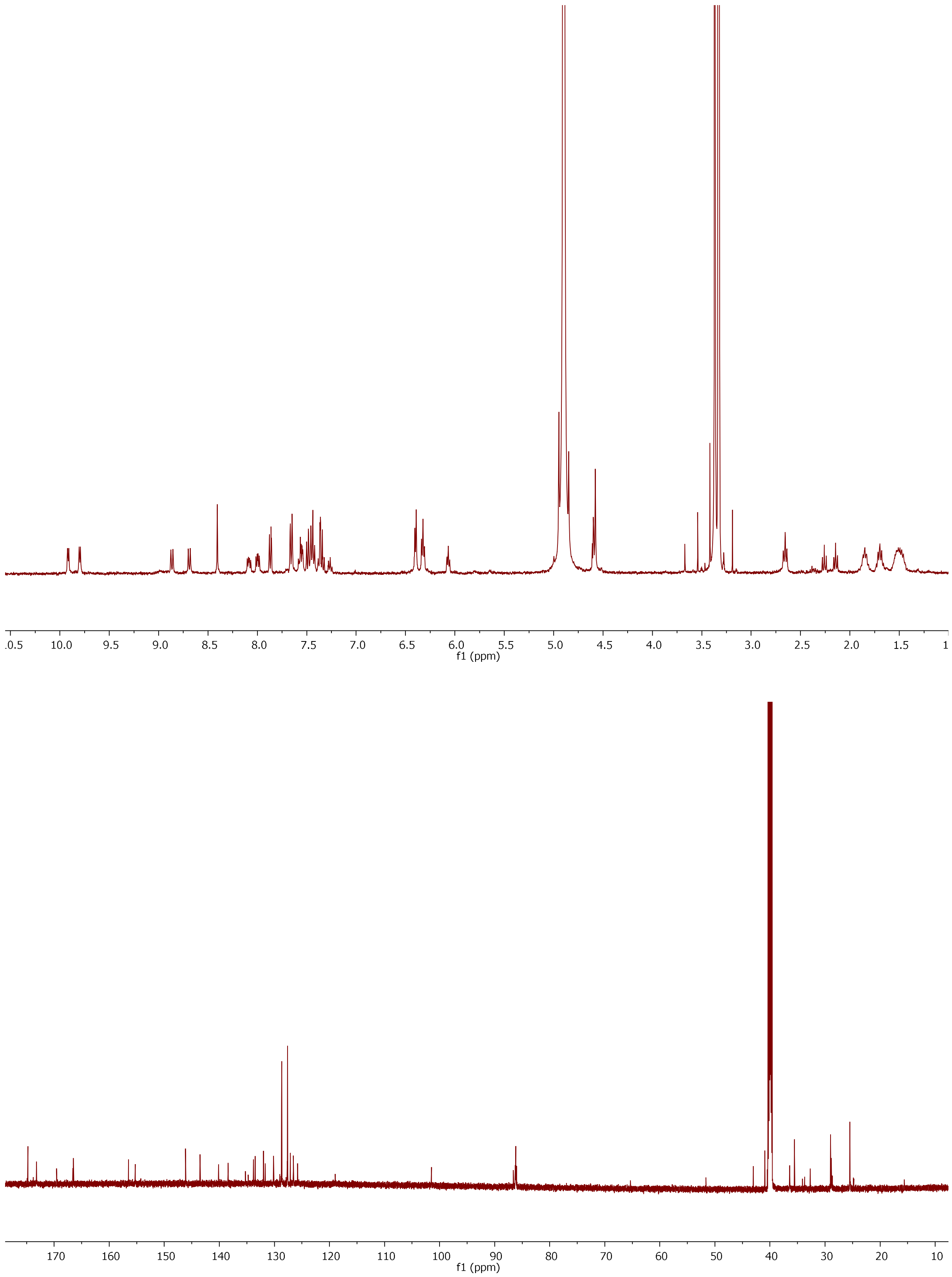


Fig S41. $^{1}$H-NMR spectrum (*upper*, DMSO-$d_6$, 700 MHz, 298 K) and $^{13}$C-NMR spectrum (*lower*, DMSO-$d_6$, 175 MHz, 298 K) of complex **8c**.

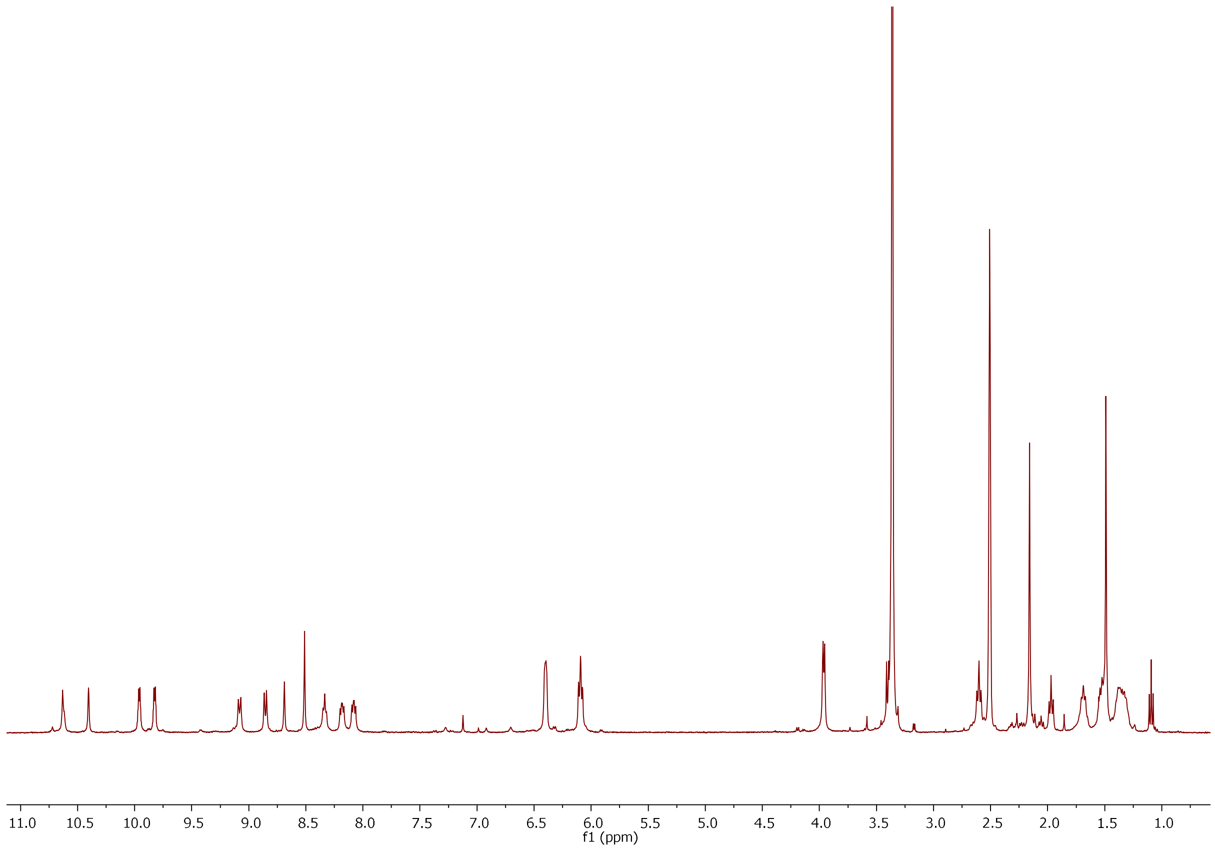


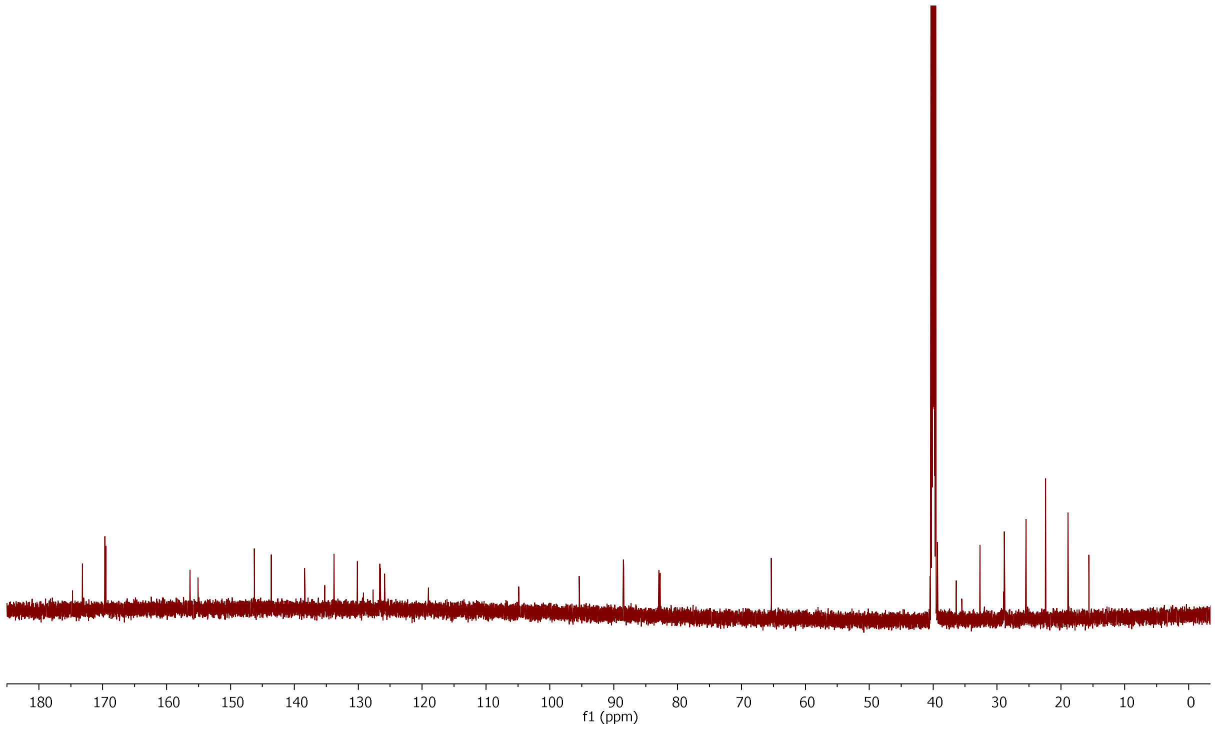


Fig S42. $^{1}$H-NMR spectrum (*upper*, DMSO-d$_6$, 700 MHz, 298 K) and $^{13}$C-NMR spectrum (*lower*, DMSO-d$_6$, 175 MHz, 298 K) of complex **8d**.

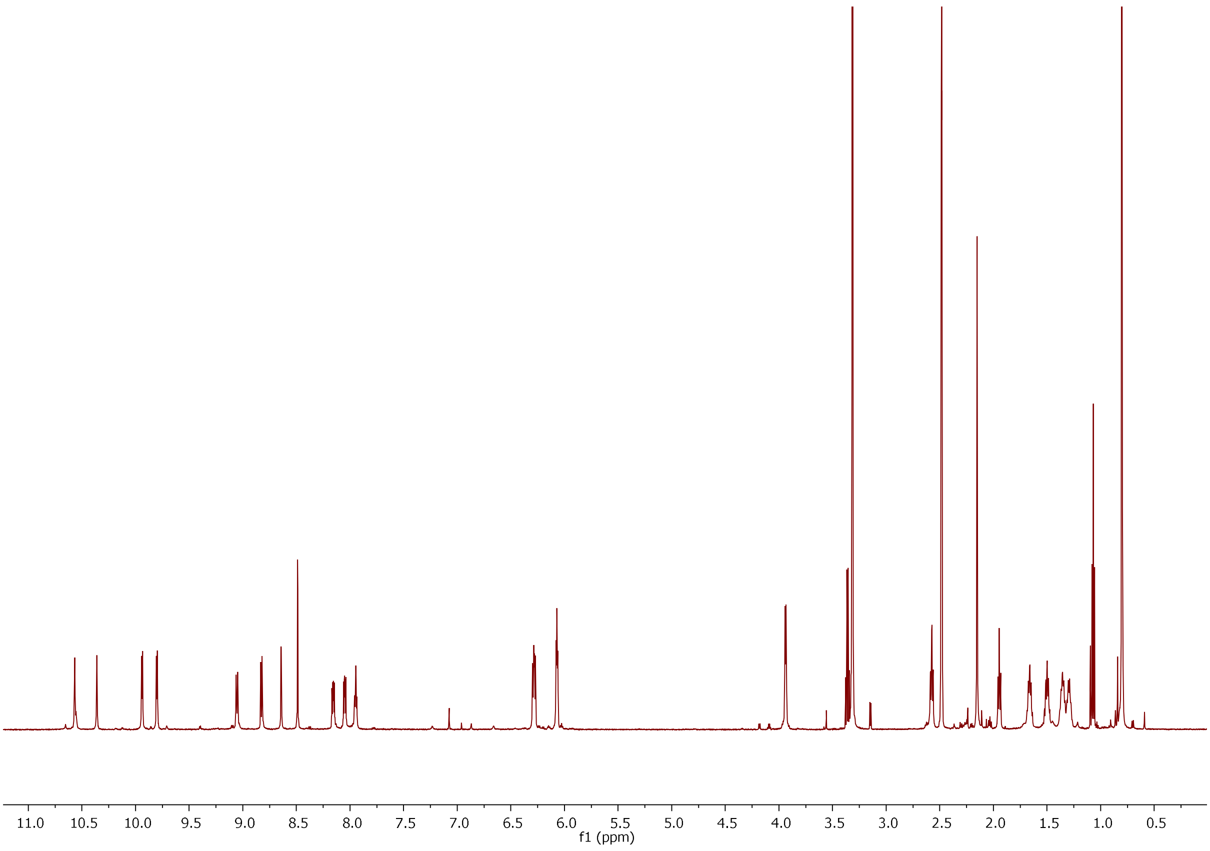


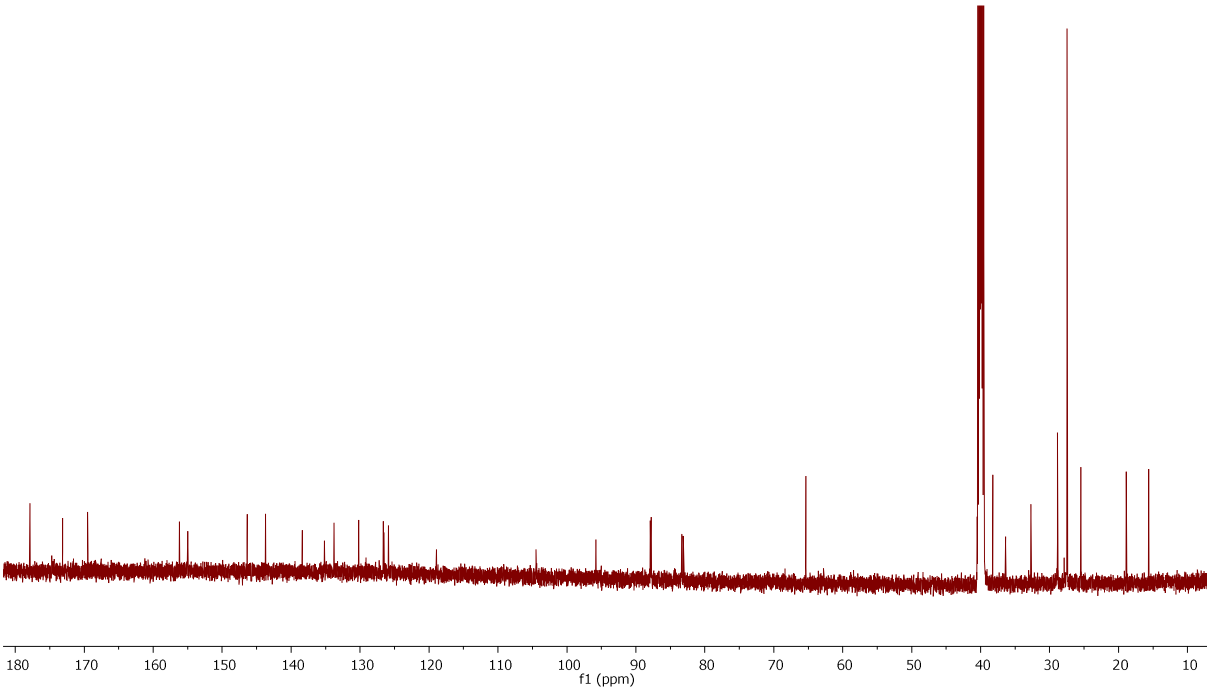


Fig S43. $^{1}$H-NMR spectrum (*upper*, DMSO-$d_6$, 700 MHz, 298 K) and $^{13}$C-NMR spectrum (*lower*, DMSO-$d_6$, 175 MHz, 298 K) of complex **8e**.

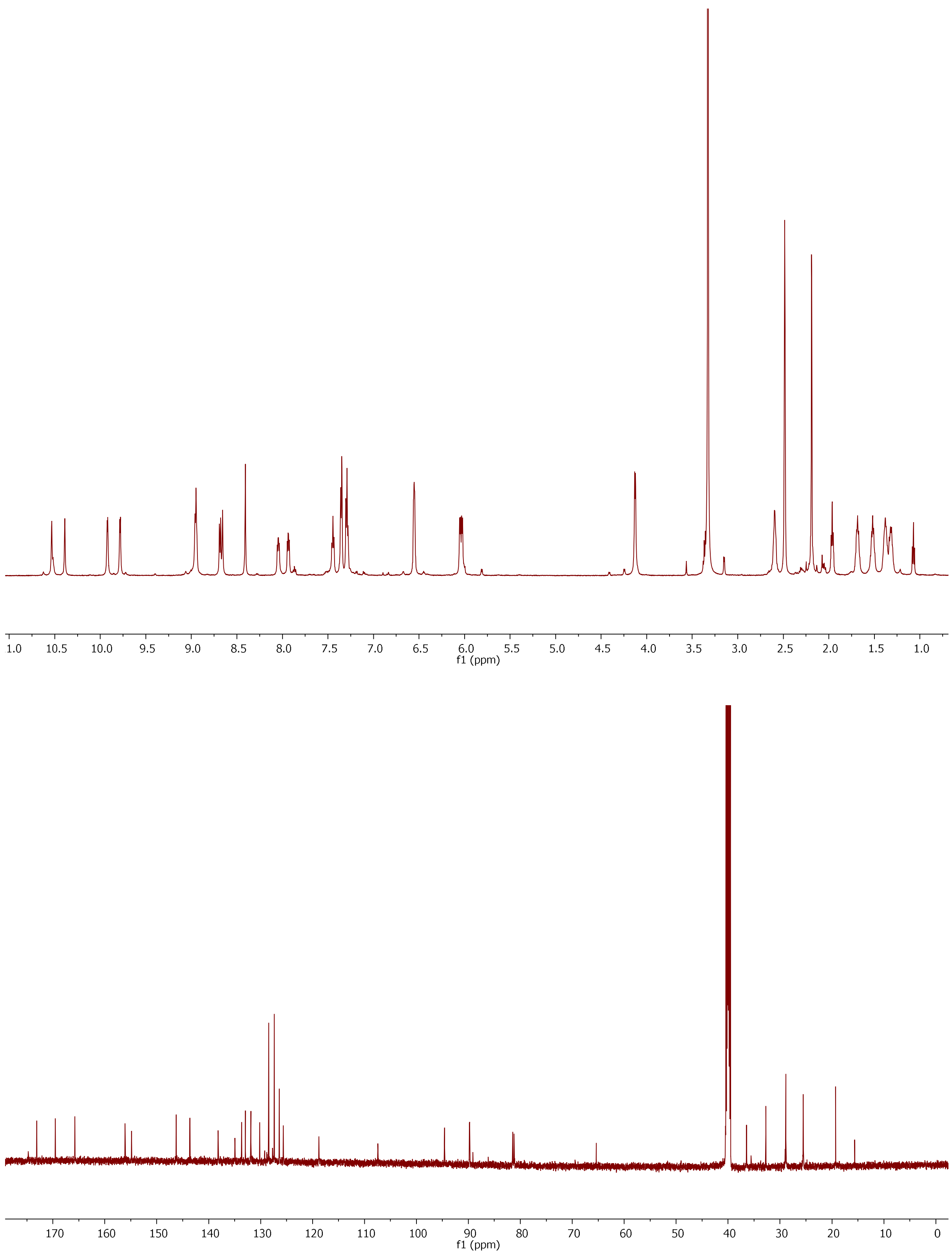


Fig S44. $^{1}$H-NMR spectrum (*upper*, DMSO-d$_6$, 700 MHz, 298 K) and $^{13}$C-NMR spectrum (*lower*, DMSO-d$_6$, 175 MHz, 298 K) of complex **8f**.

## 10.HPLC Traces

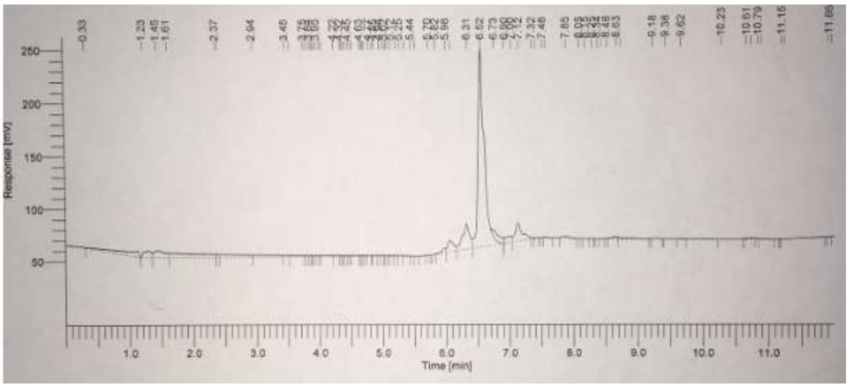


Fig S45. HPLC trace of complex **8a**

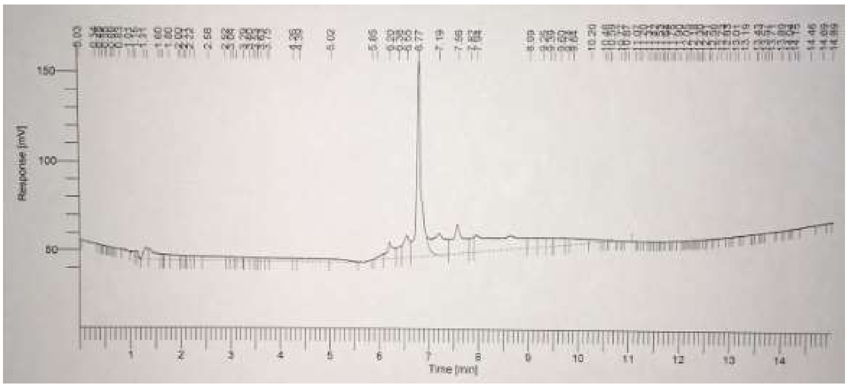


Fig S46. HPLC trace of complex **8b**

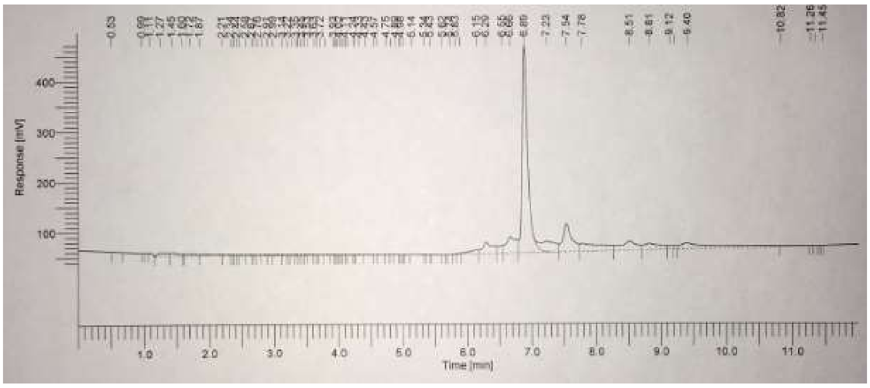


Fig S47. HPLC trace of complex **8c**

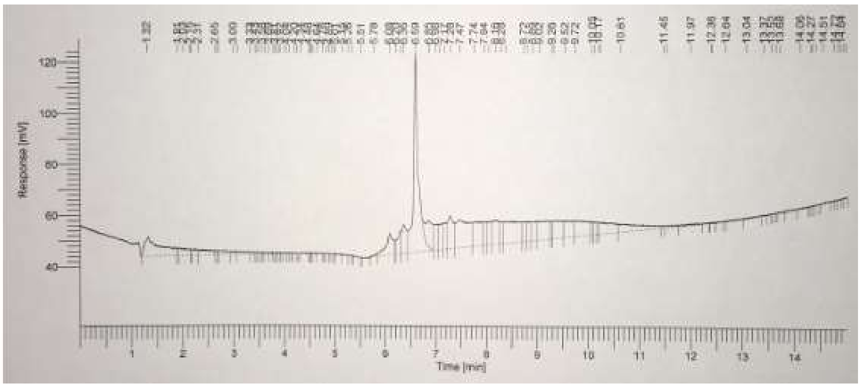


Fig S48. HPLC trace of complex **8d**

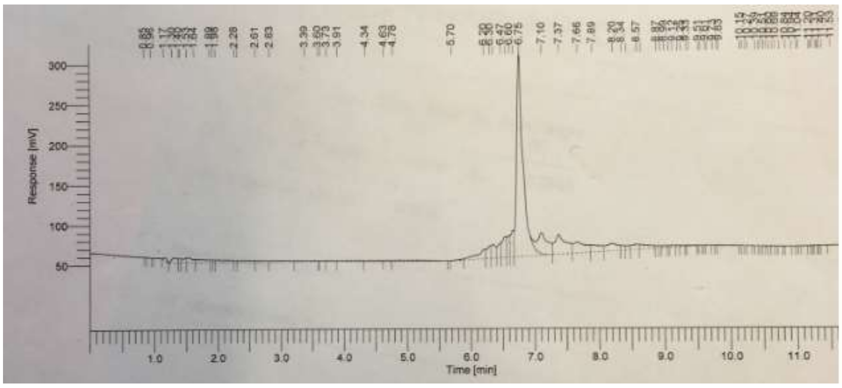


Fig S49. HPLC trace of complex **8e.**

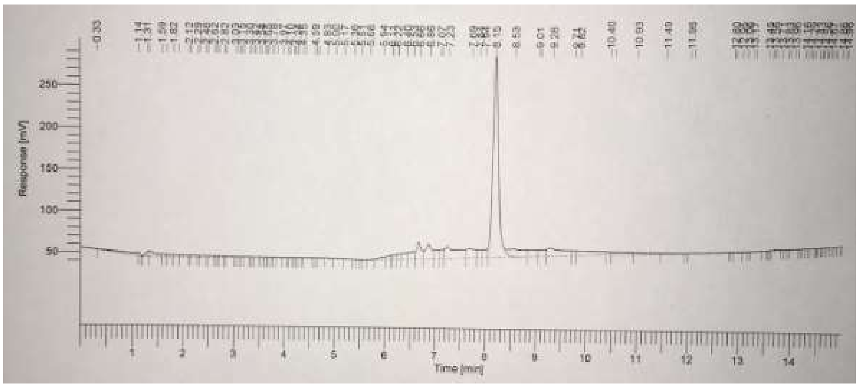


Fig S50. HPLC trace of complex **8f.**

## 11. References


1 A. T. Frawley, H. V. Linford, M. Starck, R. Pal and D. Parker, *Chem. Sci.*, 2018, **9**, 1042.

2 F. H. Allen, O. Kennard, D. G. Watson, L. Brammer, A. G. Orpen and R. Taylor, *J. Chem. Soc. Perkin Trans. 2*, 1987, S1.

3 T. Sterling and J. J. Irwin, *J. Chem. Inf. Model.*, 2015, **55**, 2324.

4 G. Jones, P. Willett, R. C. Glen, A. R. Leach and R. Taylor, *J. Mol. Biol.*, 1997, **267**, 727.

5 E. F. Pettersen, T. D. Goddard, C. C. Huang, G. S. Couch, D. M. Greenblatt, E. C. Meng and T. E. Ferrin, *J. Comput. Chem.*, 2004, **25**, 1605.
